# Determination of reference intervals for common chemistry and immunoassay tests for Kenyan adults based on an internationally harmonized protocol and up-to-date statistical methods

**DOI:** 10.1371/journal.pone.0235234

**Published:** 2020-07-09

**Authors:** Geoffrey Omuse, Kiyoshi Ichihara, Daniel Maina, Mariza Hoffman, Elizabeth Kagotho, Alice Kanyua, Jane Mwangi, Caroline Wambua, Angela Amayo, Peter Ojwang, Zul Premji, Rajiv Erasmus

**Affiliations:** 1 Department of Pathology, Aga Khan University, Nairobi, Kenya; 2 Division of Chemical Pathology, Department of Pathology, Stellenbosch University, Cape Town, South Africa; 3 Faculty of Health Sciences, Yamaguchi University Graduate School of Medicine, Ube, Japan; 4 Chemical Pathology, Pathcare, Cape Town, South Africa; 5 Nairobi Hospital, Nairobi, Kenya; 6 Pathcare, Nairobi, Kenya; 7 Department of Pathology, University of Nairobi, Nairobi, Kenya; 8 Department of Pathology, Maseno University, Maseno, Kenya; 9 Formerly of Muhimbili University of Health and Allied Sciences, Dar es Salaam, Tanzania; Copenhagen University Hospital Holbæk, DENMARK

## Abstract

**Background:**

Due to a lack of reliable reference intervals (RIs) for Kenya, we set out to determine RIs for 40 common chemistry and immunoassay tests as part of the IFCC global RI project.

**Methods:**

Apparently healthy adults aged 18–65 years were recruited according to a harmonized protocol and samples analyzed using Beckman-Coulter analyzers. Value assigned serum panels were measured to standardize chemistry results. The need for partitioning reference values by sex and age was based on between-subgroup differences expressed as standard deviation ratio (SDR) or bias in lower or upper limits (LLs and ULs) of the RI. RIs were derived using a parametric method with/without latent abnormal value exclusion (LAVE).

**Results:**

Sex-specific RIs were required for uric acid, creatinine, total bilirubin (TBil), total cholesterol (TC), ALT, AST, CK, GGT, transferrin, transferrin saturation (TfSat) and immunoglobulin-M. Age-specific RIs were required for glucose and triglyceride for both sexes, and for urea, magnesium, TC, HDL-cholesterol ratio, ALP, and ferritin for females. LAVE was effective in optimizing RIs for AST, ALT, GGT iron-markers and CRP by reducing influence of latent anemia and metabolic diseases. Thyroid profile RIs were derived after excluding volunteers with anti-thyroid antibodies. Kenyan RIs were comparable to those of other countries participating in the global study with a few exceptions such as higher ULs for TBil and CRP.

**Conclusions:**

Kenyan RIs for major analytes were established using harmonized protocol from well-defined reference individuals. Standardized RIs for chemistry analytes can be shared across sub-Saharan African laboratories with similar ethnic and life-style profile.

## Introduction

Reference intervals (RIs) are an integral part of laboratory reports as they assist clinicians in interpretation of results. RIs should be population specific to ensure appropriate interpretation. Unfortunately, many clinical laboratories in sub-Saharan Africa (SSA) adopt RIs provided by manufacturers of laboratory reagents/equipment without verifying them as recommended by the Clinical Laboratory Standards Institute (CLSI) [1]. This could result in inaccurate interpretation of quantitative laboratory results leading to medical errors. Saathoff *et al* carried out a study in the Mbeya region, south-western Tanzania and found marked differences in RIs from the United States (US), Tanzania and other SSA countries. Overall, only 80.9% of reference values (RVs) for clinical chemistry tests from healthy individuals in Tanzania would have been classified as normal as per the US RIs published by Kratz *et al* [2].

The International Federation of Clinical Chemistry (IFCC) under its Committee on Reference Intervals and Decision Limits (C-RIDL) has been carrying out a global RI study using a protocol that harmonizes the pre-analytical, analytical and post-analytical study processes to ensure ease of comparison of derived RIs across different regions, countries and ethnicities [3].

An interim report of the global RI study comprising data from 12 countries identified between ethnic group differences in both males and females for serum total protein (TP), albumin (Alb), total bilirubin (TBil), high density lipoprotein cholesterol (HDL-C), magnesium (Mg), C-reactive protein (CRP), IgG, complement 3 (C3), vitamin B12, and folate. Females were found to generally have more pronounced age-related changes in RVs. Ethnic differences in BMI-related changes was also demonstrated. The only African country whose data were included in the interim report was South Africa where comparisons between black South Africans and Caucasian / mixed race showed much higher levels of CRP in the black South Africans [4].

The Kenyan study was undertaken to explore sources of variation of RVs, to derive country specific RIs and to standardize the RIs by use of a value-assigned panel of sera [4] intended for nationwide use and international comparison.

## Materials and methods

The methodology used in recruiting participants for the study, sample collection, handling and analysis has previously been published [5]. The study was approved by the Aga Khan University Hospital Nairobi (2014/REC-46) and Stellenbosch University (S16/10/219) Health Research Ethics Committees. The study was conducted in conformity with the Declaration of Helsinki.

### Study population

Recruitment of study participants in Kenya was carried out between January and October 2015 in several counties. Majority were urban dwellers from the capital city Nairobi, Kiambu county in central Kenya, Kisii County in western Kenya, and Nakuru County based in the Great Rift Valley.

### Inclusion and exclusion criteria

Inclusion of participants was limited to healthy adults aged 18–65 years stratified into 4 age groups: 18–29, 30–39, 40–49 and 50–65 years. Exclusion criteria included individuals with a body mass index (BMI) >35 kg/m^2^, consumption of ethanol ≥70 g per day, smoking >20 cigarettes per day, taking regular medication for a chronic disease (diabetes mellitus, hypertension, hyperlipidemia, allergic disorders, depression), recent (< 15 days) recovery from acute illness, injury or surgery requiring hospitalization, carrier of HBV, HCV or HIV, pregnant or within 1 year after delivery. Written informed consent was obtained after written/verbal explanation of the study. Those with any chronic disease were excluded except for individuals aged 50–65 years who had well controlled hypertension taking up to 2 drugs. A single measurement of blood pressure, abdominal circumference and BMI was done after filling the study questionnaire.

### Blood collection and handling

Blood samples were collected by trained phlebotomists into a serum separator tube for all analytes tested in serum, lithium heparin tube for troponin I, sodium fluoride tube for plasma glucose. Serum and plasma samples requiring centrifugation were spun 2–4 hours after collection and stored at −80°C at the Aga Khan University Hospital, Nairobi (AKUHN). Centrifugation was done at 2000g, for 10 mins in a non-refrigerated centrifuge (Beckman coulter, Allegra X-30, Brea, California, US) These were subsequently shipped frozen to the PathCare reference laboratory in Cape Town, South Africa for analysis. We also drew 2mL of whole blood for testing hematology parameters using Beckman-Coulter ACT5-DIFF-CP analyser (Brea, California, US), tested in PathCare Nairobi. The test results were primarily used for establishing RIs for hematology parameters [5], but they were referred to in this study for secondary exclusion of individuals with latent anemia or inflammation.

### Measurements

The analysis of all serum specimens was performed in batches on the Beckman Coulter AU 5800 (Brea, California, US) for chemistry assays and DXI (Brea, California, US) for immunoassays as summarized in Table 1.

**Table 1:** Summary of tests, equipment, assay methods and analytical performance.

Analyte	Abbreviation	Method	Units	Between Run CV
Sodium	Na	Ion selective electrode / diluted (indirect)	mmol/L	1.3
Potassium	K	Ion selective electrode / diluted (indirect)	mmol/L	3.8
Chloride	Cl	Ion selective electrode / diluted (indirect)	mmol/L	1.4
Urea	Urea	Urease	mmol/L	2.4
Creatinine	Cre	Modified kinetic Jaffè	μmol/L	1.6
Total Protein	TP	Biuret	g/L	4.6
Albumin	Alb	Bromocresol Green dye binding	g/L	2.8
Total Bilirubin	TBil	Diazonium salt	μmol/L	7.7
Gamma-glutamyl transferase	GGT	Gamma-glutamyl-3-carboxy-4-nitroanilide	IU/L	3.3
Alkaline phosphatase	ALP	P-nitro-phenylphosphate hydrolysis	IU/L	4.0
Lactate dehydrogenase	LDH	Lactate to Pyruvate	IU/L	3.2
Calcium	Ca	Arsenazo III dye	mmol/L	1.4
Magnesium	Mg	Xylidyl blue	mmol/L	1.9
Phosphate	IP	Molybdate hydolysis	mmol/L	1.8
Lipase	Lip	1, 2-Diglyceride hydrolysis	U/L	8.0
Total cholesterol	TC	Cholesterol oxidase	mmol/L	1.2
Triglycerides	TG	Glycerol phosphate oxidase	mmol/L	4.1
High density lipoprotein cholesterol	HDL-C	Two phase selective accelerator detergent	mmol/L	2.0
Low density lipoprotein cholesterol	LDL-C	Two phase selective accelerator detergent	mmol/L	1.6
Uric acid	UA	Modified Trinder reaction with Uricase	mmol/L	1.2
High sensitive c reactive protein	CRP	Turbidimetry	mg/L	1.8
Amylase	AMY	2-chloro-4-nitrophenyl-α-D-maltotrioside	U/L	8.1
Immunoglobulin A	IgA	Turbidimetry	g/L	1.3
Immunoglobulin G	IgG	Turbidimetry	g/L	1.1
Immunoglobulin M	IgM	Turbidimetry	g/L	7.0
Alanine aminotransferase	ALT	NADH (without P-5′-P)	U/L	28.1
Aspartate aminotransferase	AST	NADH (without P-5′-P)	U/L	5.6
Creatinine kinase	CK	Creatine phosphate dephosphorilysation	U/L	6.5
Iron	Fe	2, 4, 6-Tri-(2-pyridyl)-5-triazine chromogen	μmol/L	4.2
Transferrin	Tf	Turbidimetry	g/L	2.6
Anti-thyroglobulin	TgAb	Two-site immune—enzymatic immunoassay	IU/ml	4.8
Anti-thyroid peroxidase	TPOAb	Two-site immune—enzymatic immunoassay	IU/ml	28.8
Thyroid stimulating hormone	TSH	Two-site immune—enzymatic immunoassay	mIU/L	13.0
Free thyroxine	FT4	Two-site immune—enzymatic immunoassay	pmol/L	8.1
Free tri-iodothyronine	FT3	Two-site immune—enzymatic immunoassay	pmol/L	4.8
Ferritin	Fer	Turbidimetry	μg/L	8.2
Prostate specific antigen	PSA	Two-site immune—enzymatic immunoassay	μg/L	2.3

As calculated parameters, globulin (Glb) was computed as TP—Alb; non-HDL-C as TC—HDL-C; HDL-C ratio (HDLrat) as TC/HDL-C.

### Quality control

The PathCare reference laboratory is accredited by the South African National Accreditation System. For purposes of the global RI study, all participating laboratories received a panel of sera produced by the C-RIDL in 2014 that had assigned values [4]. This panel was measured by participating laboratories to enable recalibration of RVs using linear regression analysis. It also allowed for alignment of RVs across different countries by all-pairwise comparison of test results.

### Statistical analysis

In order to assess sex, age and BMI as sources of variation, we adopted the standard deviation ratio (SDR), which represents a ratio of between-subgroup SD (variation of the subgroup means from grand mean) to between-individual SD (approximately 0.25 the width of RI). For calculating SDRs, we first performed 2-level nested ANOVA to compute between-sex SD and between-age SD after partitioning age at 30, 40, and 50 years. With the results, the SDR for between-sex SD (SDRsex) and between-age SD (SDRage) were calculated as a ratio to the residual SD that corresponds to roughly between-individual SD or SD comprizing RI (SD_RI_. Since between-age variation changes by sex, we also computed SDRage for each sex by one way ANOVA. We considered SDR≥0.40 as a primary criterion for judging the need for partitioning RVs by sex and/or age [5].

**Fig 1:**
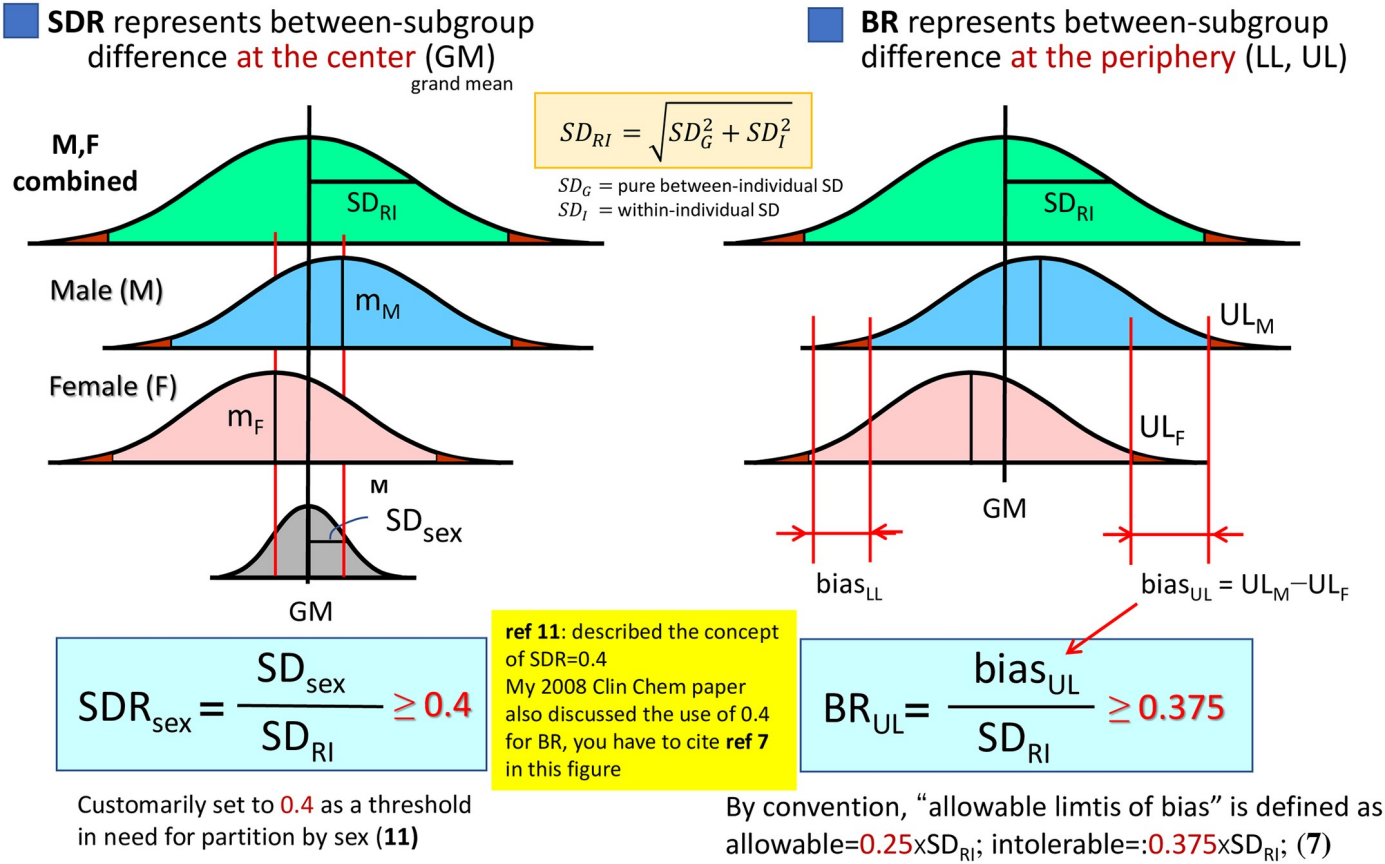
SD ratio (SDR) vs. bias ratio (BR) as a measure of between-subgroup differences. **SDR** represents between-subgroup differences at the center of distributions, while **BR** represents between-subgroup differences at the periphery (LL or UL) of the distributions. The numerator of SDR is between-subgroup SD (or SDsex, if sub-grouped by sex), while that of BR is a difference of LLs or ULs.

However, SDR represents between-subgroup difference at the center of the RV distribution, which may not reflect the difference (bias) at LL or UL (ΔLL or ΔUL). Therefore, we also evaluated ΔLL or ΔUL as its ratio to SD comprising RI (SD_RI_) [= |UL−LL|/3.92] and expressed it as bias ratio (BR) at LL or UL (BR_LL_ or BR_UL_). For example, the formula for determining BR for sex was:

$${\text{BR}}_{\text{LL}}=\frac{\left|{\text{LL}}_{M}-{\text{LL}}_{F}\right|}{\left({\text{UL}}_{MF}-LL_{MF}\right)/3.92},{\;\text{BR}}_{\text{UL}}=\frac{\left|{\text{UL}}_{M}-{\text{UL}}_{F}\right|}{\left({\text{UL}}_{MF}-LL_{MF}\right)/3.92}$$

where subscripts of MF, M, and F attached to LL or UL indicate the RI without partition by sex (for male+female) and the RIs after partition by male and female, respectively [6]. The same calculation was done for judging the need for age-specific RIs by setting LL, UL with/without partitioning by age. The distinction of the concepts between SDR and BR is illustrated in Fig 1.

**Fig 2:**
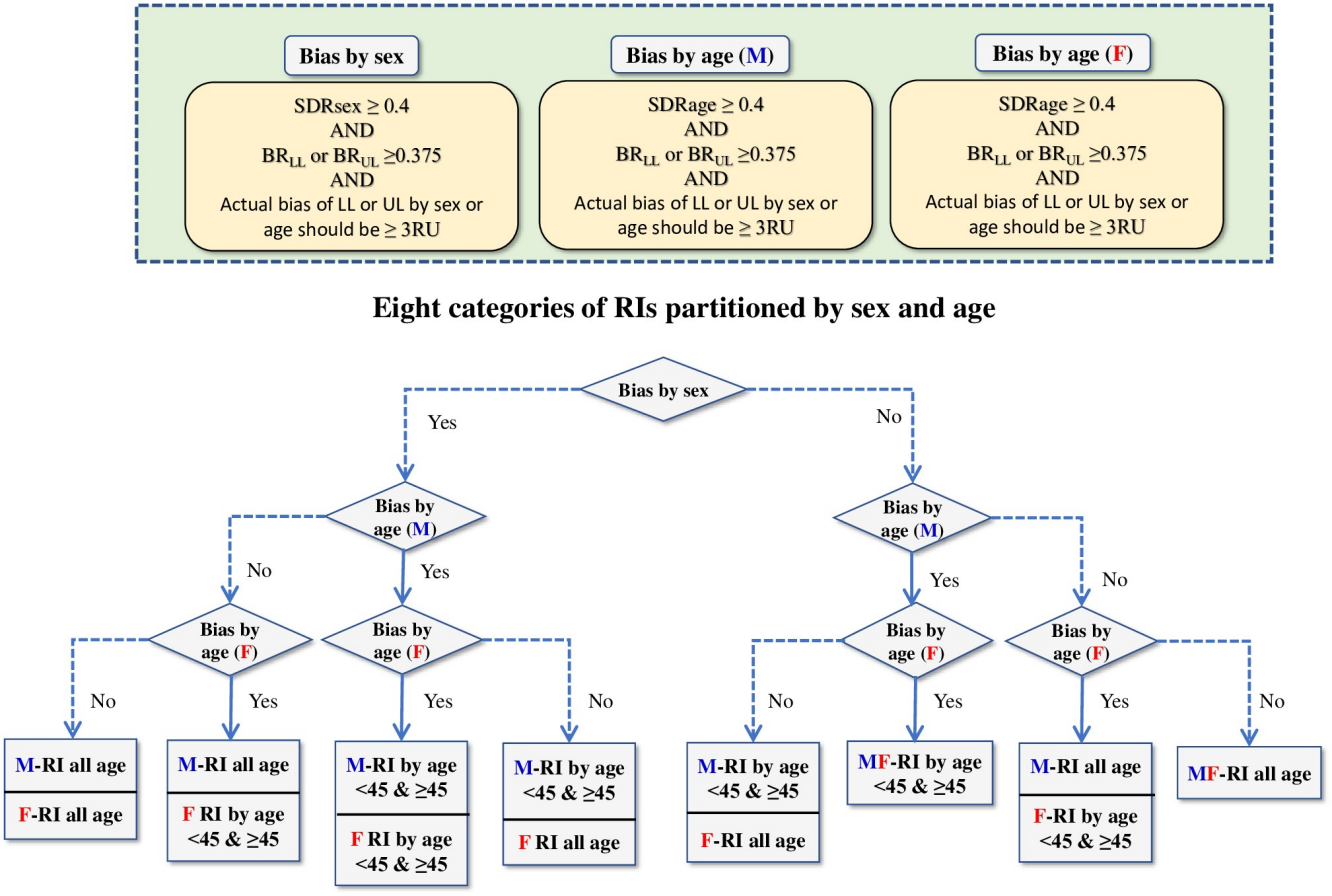
Scheme for partitioning RVs by sex and age. We adopted this flow-chart in judging the needs for partitioning RVs by sex and age. We defined between-sex (or -age) subgroup bias in reference to the three points: 1) SDR>0.4 that represents the between-subgroup bias at the center of RV distribution, 2) BR>0.375 that represents the between-subgroup bias at the limits (LL or UL) of RV distribution, and 3) the actual difference (bias) ≤ three reporting unit (RP). There were eight possible choices for the partitioning.

In setting the threshold for the bias ratios, we followed the convention of allowable limits of bias in measurements: $\sqrt{{SD}_{I}^{2}+{SD}_{G}^{2}}\leq 0.375$, where SD_I_ and SD_G_ represent within-individual and between-individual SD [7]. Since SD_RI_ or the denominator of BR is composed of both SD_I_ and SD_G_ and equal to $\sqrt{{SD}_{I}^{2}+{SD}_{G}^{2}}$, we set 0.375 as a threshold for BR. This scheme was adopted in recent papers [8,9]. On the other hand, both SDR and BR depend on their common denominator, SD_RI_. For example, when the RI is narrow, both ratios can be inflated. Conversely, when the RI is wide, both ratios are suppressed. In order to cope with such situations, we set a pragmatic third criterion that between-subgroup bias at LL or UL (ΔLL or ΔUL) should be equal to or more than 3 times the “reporting unit (RU)” to allow partitioning of RVs. RU represents a unit of value for reporting test results. If the number of digits below the decimal point in reporting test results is 2, 1, or 0, RU is 0.01, 0.1, or 1, respectively. The flow of logic in deciding the need for partitioning by sex or age is shown in Fig 2.

RIs were determined using both parametric and non-parametric methods before and after applying the latent abnormal values exclusion (LAVE) method [10,11]. For the non-parametric method, the RVs coinciding with the 2.5^th^ and 97.5^th^ percentiles after sorting the data in ascending order were used as the lower and upper limits (LL, UL) of the RI. For the parametric method, the RVs were transformed into a Gaussian form by the Box-Cox power transformation formula, and then mean±1.96 SD was computed as the central 95% limits (LL−UL) under the transformed scale, which were then reverse transformed to get the LL and UL in the original scale [11].

As a measure for secondary exclusion of abnormal values, we tried to apply LAVE in deriving the RI both by parametric and nonparametric methods [11]. For LAVE, we primarily used the following set of reference tests: Alb, Glb, UA, Glu, non-HDL, TG, ALT, AST, LDH, CK, GGT, and CRP, which were meant for excluding individuals with inappropriate values in the nutritional, inflammatory, or muscular damage markers. As an exception, for iron related analytes (Fe, Ferr, Tf, and TfSat), we set hemoglobin (Hb), hematocrit (Hct), and mid-corpuscular volume (MCV) in addition to the four iron markers as the reference tests. For proteins (TP, Alb, Glb, IgG/A/M, CRP), we set all the seven tests plus white blood cell and platelet counts as the reference tests.

**Fig 3:**
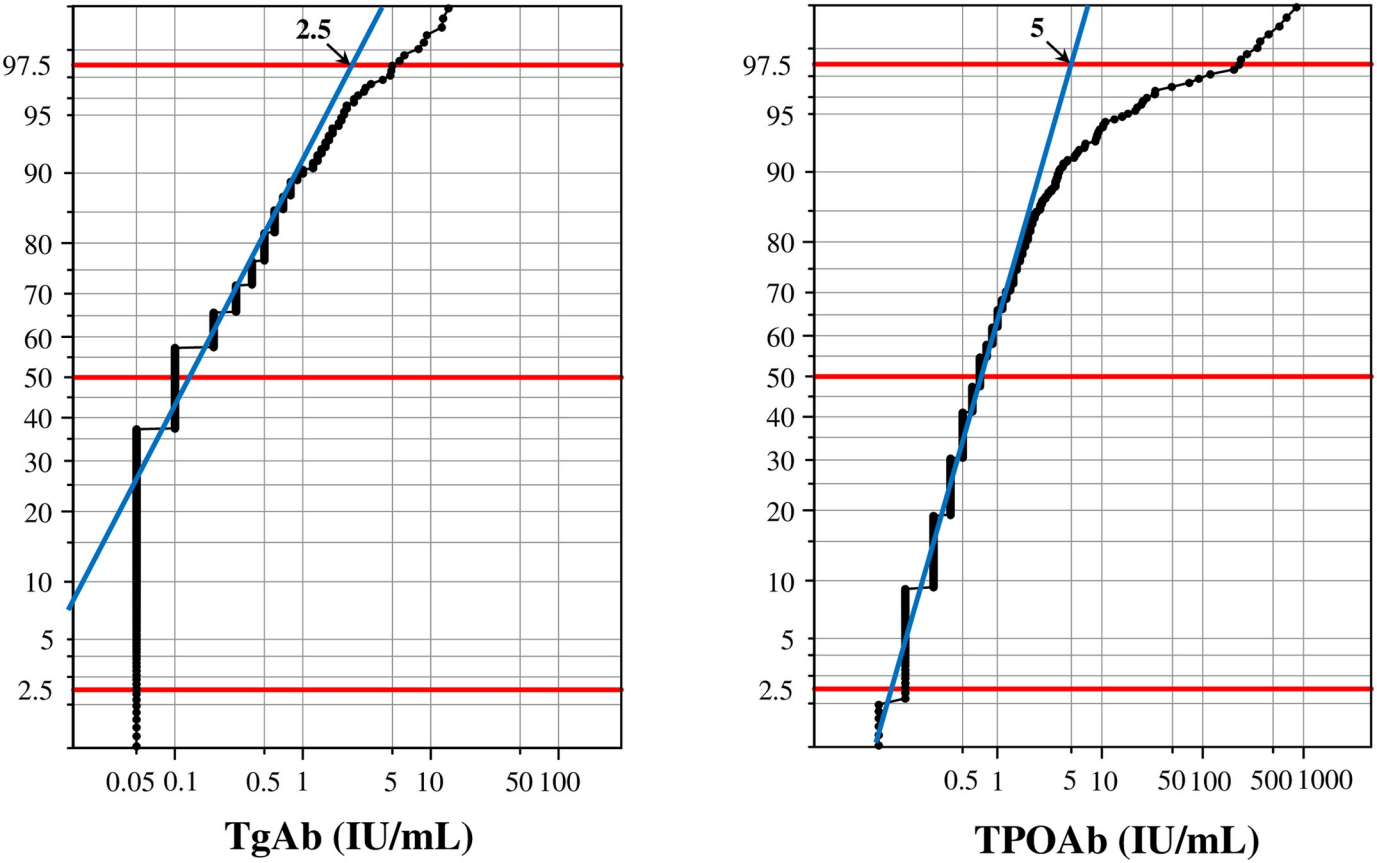
Determination of cutoff values for TgAb and TPOAb based on the probability paper plot. Cutoff values for thyroglobulin antibody (TgAb), and thyroid peroxidase antibody (TPOAb) were empirically determined by use of the probability paper plot. Y-axis represents cumulative frequencies of RV distribution, while x-axis was drawn in logarithmic scale. We assumed that both antibodies are specific to autoimmune thyroiditis (AIT), and thus extreme values in the tail constitutes a group of values from AIT cases. Therefore, we determined the cutoff value as an intersection of central linear part with the horizontal line at 97.5%.

On the other hand, for determination of RIs for the thyroid panel, the LAVE procedure was not applied because reference tests associated with the thyroid panel were not available. Rather, we first estimated the cutoff values for anti-thyroglobulin antibody (TgAb), and anti-thyroid peroxidase antibody (TPOAb) from the probability paper plot drawn with x-axis in logarithmic scale, as an intersection of the central linear part with the horizontal line at 97.5 percentile as shown in Fig 3.

For judging the need for adopting LAVE, we computed BR_LL_ and BR_UL_ by setting LLs and ULs of RIs with/without LAVE: i.e., SD_RI_ in the denominator was set to the RI by the LAVE procedure. The 90% confidence intervals (CIs) of LL and UL were derived by the bootstrap method with resampling of the final set of RVs and repeated computations of LL and UL for 50 iterations. Accordingly, final RIs were determined as the averages of LL and UL thus derived.

## Results

### Profile of the subjects

**Fig 4:**
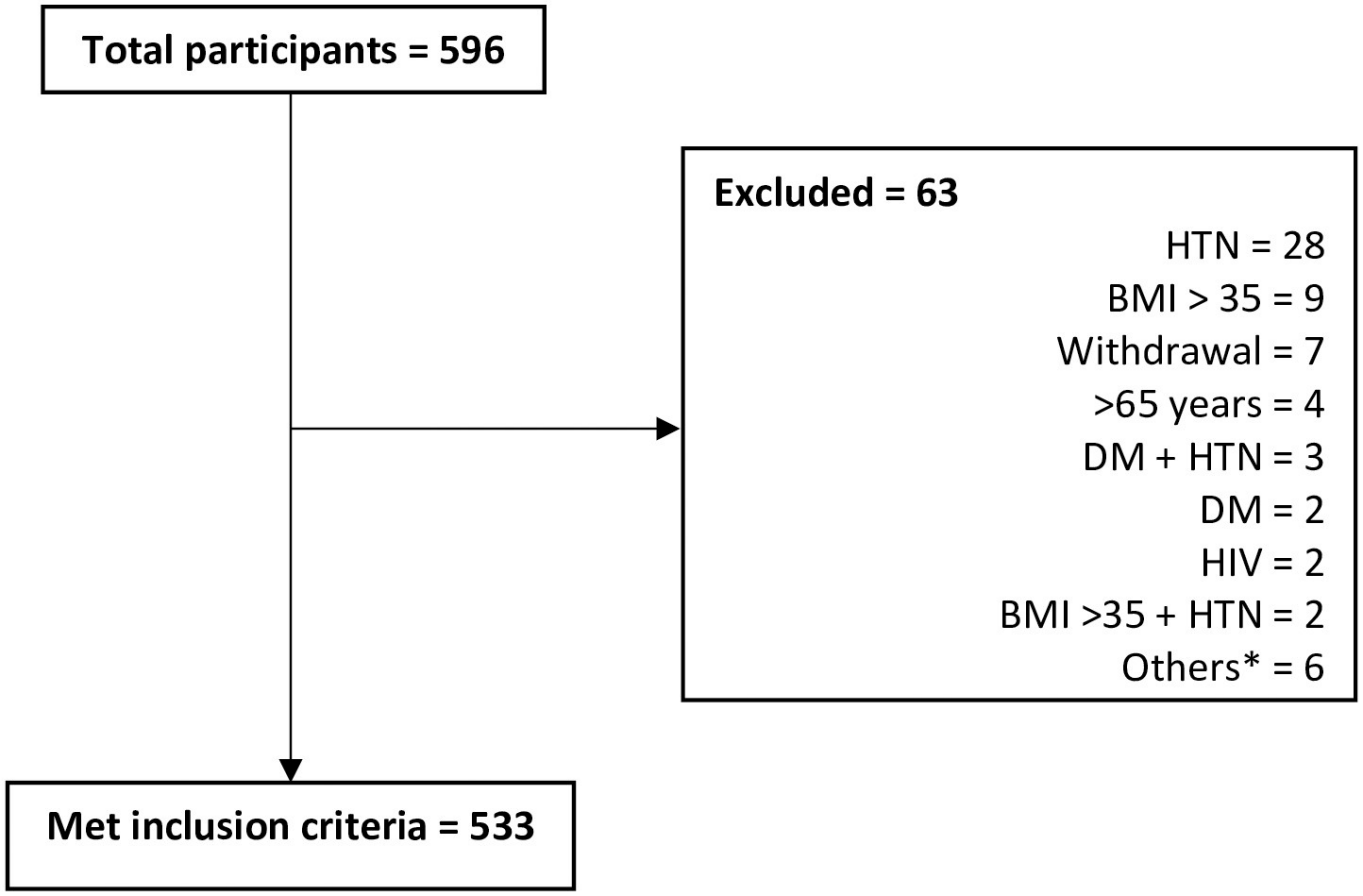
Study flowchart. BMI: body mass index, CBC: complete blood count, DM: diabetes mellitus, HIV: Human Immunodeficiency Virus, HTN: hypertension. *On antibiotics, blood donation in past 3 months, on treatment for hypothyroidism, > 65 years, > 65 years and hypertensive, rheumatic heart disease, prostate cancer.

Out of 596 volunteers, 533 met the inclusion criteria: 260 (48.8%) males and 273 (51.2%) females. The main reason for exclusion was hypertension as shown in Fig 4.

The median age was 39 years with a range of 18–65 years. The participant characteristics are summarized in Table 2.

**Table 2:** Descriptive characteristics of participants.

	Male (n = 260)		Female (n = 273)		Total (n = 533)	
	Median (IQR)	Min-Max	Median (IQR)	Min-Max	Median (IQR)	Min-Max
Age (years)	39 (18)	20–65	39 (21)	18–64	39 (20)	18–65
Height (cms)	172 (8)	156–191	160 (9)	143–191	167 (13)	143–191
Weight (kg)	74 (19)	46–116	68 (16)	38–109	70 (18)	38–116
BMI (kg/m^2^)	24.9 (5.6)	16.3–34.9	26.1 (6.3)	17.1–38.1	25.5 (5.9)	16.3–38.1
BSA (m^2^)	1.88 (0.24)	1.44–2.36	1.72 (0.20)	1.27–2.24	1.78 (0.22)	1.27–2.36
AC (cm)	91 (15)	65–124	86 (16)	64–115	89 (17)	64–124
BP Systolic (mmHg)	127 (18)	84–179	128 (18)	118 (20)	124 (21)	77–194
BP Diastolic (mmHg)	81 (12)	56–101	79 (14)	57–112	80 (14)	56–112

AC: abdominal circumference; BMI: body mass index; BSA: body surface area; IQR: interquartile range

### Sources of variation

Sex, age and BMI as sources of variation were explored with SDR≥0.4 regarded as being significant as shown in Table 3.

**Table 3:** Standard deviation ratios for between sex and age.

	SDRsex	SDRage				SDRsex	SDRage		
	M+F	M+F	M	F		M+F	M+F	M	F
TP	0.00	0.36	0.38	0.35	ALT	**0.56**	**0.42**	0.31	**0.52**
Alb	**0.50**	**0.54**	**0.56**	**0.53**	AST	**0.48**	0.26	0.09	0.34
Glb	0.32	0.00	0.00	0.00	ALP	0.00	**0.47**	0.16	**0.66**
Urea	0.30	0.32	0.21	**0.42**	AMY	0.05	0.00	0.00	0.00
eGFR	0.00	0.32	0.34	0.29	LDH	0.00	0.22	0.00	0.31
UA	**0.89**	0.38	**0.41**	0.33	CK	**0.50**	0.08	0.02	0.12
Cre	**1.19**	0.24	0.30	0.14	GGT	**0.51**	0.28	0.35	0.14
TBil	**0.48**	0.27	0.21	0.32	Iron	0.39	0.09	0.10	0.08
Na	0.00	0.38	0.14	**0.47**	Ferr	**0.78**	0.26	0.22	**0.45**
K	0.00	0.23	0.22	0.25	TF	**0.43**	0.18	0.18	0.18
Cl	**0.44**	0.30	0.38	0.18	TFSat	**0.50**	0.00	0.00	0.00
Ca	0.16	0.29	0.31	0.26	CRP	0.10	0.31	0.37	0.25
IP	0.21	0.20	0.22	0.17	IgA	0.00	0.24	0.15	0.31
Mg	0.00	**0.46**	0.38	**0.52**	IgG	0.10	0.12	0.17	0.07
Glu	0.00	**0.41**	**0.44**	0.39	IgM	**0.48**	0.12	0.15	0.08
TC	0.00	**0.41**	**0.41**	**0.42**	FT3	0.12	0.04	0.00	0.09
TG	0.22	**0.58**	**0.56**	**0.60**	FT4	0.00	0.36	0.35	0.37
HDL-C	0.33	0.00	0.00	0.00	TSH	0.00	0.00	0.00	0.03
HDLrat	0.33	0.38	0.39	0.37	TgAb	0.00	0.28	0.27	0.28
nonHDL	0.00	**0.45**	**0.44**	**0.46**	TPOAb	0.09	0.04	0.06	0.03
LDL-C	0.00	**0.42**	0.40	**0.43**	Trop I	0.00	0.08	0.00	0.09
Lip	0.07	0.16	0.17	0.15	PSA			0.39	

SDR≥0.4 shown in bold; SDR≥0.5 orange background

**Fig 5:**
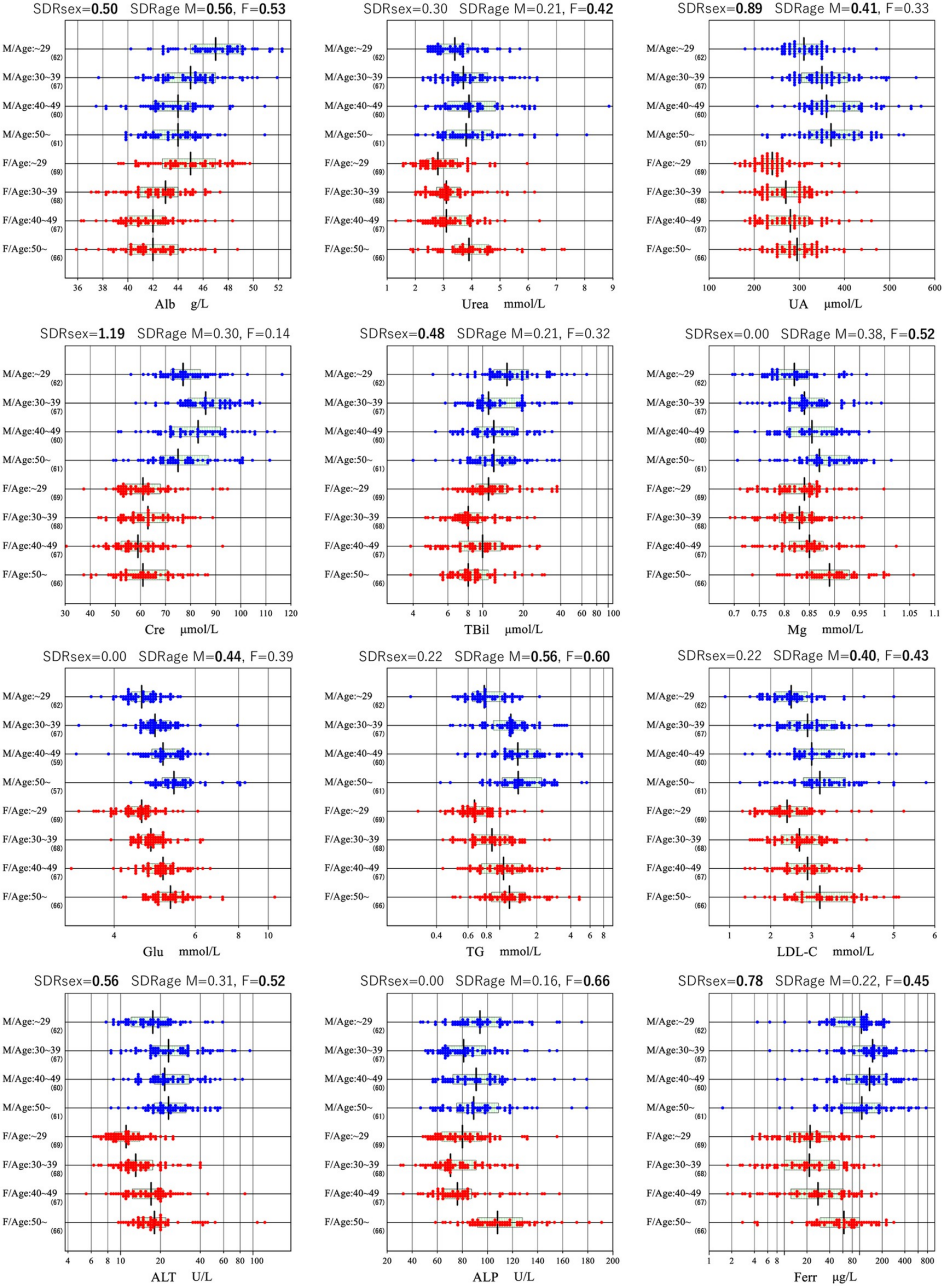
Sex and age-related changes for 12 analytes. The distributions of reference values are shown based on age and sex stratification for 12 analytes. The SDRsex and SDRage for each sex are shown at the top of each analyte chart. No secondary exclusion was performed in plotting the data. The box in each scattergram represents central 50% range and the vertical bar in the middle represents a median point.

Between-sex differences exceeded that level in Alb, UA, Cre, TBil, Cl, ALT, AST, CK, GGT, Ferr, Tf, TfSat, and IgM. Similarly, between-age subgroup differences were significant for Alb, Cl, Glu, and TG in males, and for Alb, urea, Na, Mg, Glu, TC, TG, HDLrat, LDL-C, ALT, ALP, and Ferr in females. BMI was an independent source of variation for UA, Glu, TC, HDLrat, nonHDL-C, LDL-C, ALT, AST, LDH, and GGT in males, and for CRP only in females. Graphical representations of reference value distribution sub-grouped by sex and age are shown for 12 representative analytes in Fig 5 and for all analytes in S1 Fig.

### Reference intervals

Generally, the parametric method resulted in similar or lower RI ULs, and narrower 90% CIs for the LL and UL of the RIs compared to the non-parametric method as shown in S2 Fig. Besides, the accuracy of Gaussian transformation by the parametric method was confirmed as shown in S3 Fig. Therefore, we adopted the RIs derived by the parametric method exclusively. In addition, we could not calculate a RI for Trop I because 94.7% of RVs were below detection limit. For TgAb and TPOAb, we determined cutoff values by use of the probability paper plot as 2.5 IU/ml and 5.0 IU/mL respectively as shown in Fig 3. We regarded individuals who had antibody values exceeding either of the cutoffs (7.7% of males and 13.6% of females) as possible autoimmune thyroiditis (AIT), and excluded them when calculating RIs for thyroid function tests.

The LAVE method resulted in significant differences in LL or UL (BR_UL_ or BR_LL_ > 0.375) for some analytes as shown in S1 Table. For example, UL lowered for AST, ALT, CRP; LL raised for Fe.

Based on the decision flow chart shown in Fig 2, RIs for TP, Alb, Glb, Na, K, Cl, Ca, IP, Glu, TG, HDL-C, nonHDL, Lip, AMY, LDH, CRP, Fe, IgG and IgA were not partitioned by sex as shown in Table 4. For age partitions, although the mean age of menopause for females is about 50 years, we chose 45 years as the borderline because there were limited number of subjects above the age of 50.

**Table 4:** Proposed reference intervals.

			M + F			M			F		
Item	Units	Age	n	LL	UL	n	LL	UL	n	LL	UL
TP	g/L	All	526	67	83						
Alb	g/L	~45	349	39	50						
		45~	150	39	47						
Glb	g/L	All	524	24	38						
Urea	mmol/L	All				258	2.4	6.6			
		~45							145	1.8	5.2
		45~							83	1.8	6.4
UA	mmol/L	All				259	238	532	272	178	417
Cre	μmol/L	All				260	58	109	271	45	86
TBil	μmol/L	All				257	6	43	271	5	27
Na	mmol/L	All	531	134	142						
K	mmol/L	All	531	3.4	4.8						
Cl	mmol/L	All	501	100	110						
Ca	mmol/L	All	531	2.19	2.57						
IP	mmol/L	All	533	0.78	1.42						
Mg	mmol/L	All				260	0.73	0.98			
		~45							174	0.71	0.93
		45~							99	0.76	1.01
Glu	mmol/L	~45	345	3.9	5.8						
		45~	172	4.4	7.3						
TC	mmol/L	All				260	3.1	6.9			
		~45							174	3.1	6.2
		45~							99	3.2	7.1
TG	mmol/L	~45	289	0.47	2.60						
		45~	154	0.55	3.70						
HDL-C	mmol/L	All	530	0.7	1.7						
HDLrat		All				259	2.6	7.6			
		~45							174	2.4	5.9
		45~							98	2.7	6.7
nonHDL	mmol/L	All	532	2.0	5.6						
LDL-C	mmol/L	All				260	1.6	4.8			
		~45							174	1.5	4.2
		45~							99	1.7	4.9
Lip	U/L	All	527	9	68						
ALT	IU/L	All				202	10	55	229	7	30
AST	IU/L	All				206	17	40	227	15	29
ALP	IU/L	All				260	53	153			
		~45							172	47	130
		45~							99	55	174
AMY	IU/L	All	530	47	164						
LDH	IU/L	All	530	138	257						
CK	IU/L	All				259	72	460	268	53	260
GGT	IU/L	All				199	13	90	269	10	50
CRP	mg/L	All	425	0.21	14.67						
Fe	μmol/L	All	412	8.8	28.9						
Ferr	μg/L	All				253	10	475			
		~45							173	2	150
		45~							99	0	222
Tf	g/L	All				254	1.9	3.2	206	2.0	3.5
TfSat	%	All				208	15	49	211	10	44
IgG	g/L	All	525	11.1	20.0						
IgA	g/L	All	526	1.05	4.63						
IgM	g/L	All				254	0.38	2.29	272	0.51	2.77
FT3	pmol/L	All	467	3.9	6.3						
FT4	pmol/L	All	468	7.8	14.1						
TSH	mIU/L	All	461	0.61	4.86						
PSA	μg/L	All				243	0.29	2.92			

A comparison of our RIs with those recommended by Beckman coulter and those derived from IFCC studies conducted in India, Saudi Arabia and Turkey found much higher RIs for TBil as shown in Table 5. A similar comparison that also includes studies carried out in other African countries is shown in S2 Table.

**Table 5:** Comparison of reference intervals.

		PRESENT STUDY KENYA							BECKMAN AU [22]							SAUDI ARABIA [12]							TURKEY [13]							INDIA [15]						
			M+F		M		F			M+F		M		F			M+F		M		F			M+F		M		F			M+F		M		F	
Test	Units	Age	LL	UL	LL	UL	LL	UL	Age	LL	UL	LL	UL	LL	UL	Age	LL	UL	LL	UL	LL	UL	Age	LL	UL	LL	UL	LL	UL	Age	LL	UL	LL	UL	LL	UL
TP	g/L	18–65	67	83					NA	66	83					18–65	66	83					20–79	66	82					18–65	68	86				
Alb	g/L	18–65							NA	35	52					18–65	39	50					20–79	41	49					18–65					36	47
		18–44			40	51	38	49																												
		45–65			40	47	38	46																												
Urea	mmol/L	18–65			2.4	6.6			NA	2.8	7.2					18–65			2.8	7.3	2.1	6.4								18–65			2.2	6.0		
		18–44					1.8	5.3															20–49			2.95	7.20	2.21	6.12	18–45					1.9	5.1
		45–65					1.9	6.2															50–79					2.85	7.96	46–65					2.4	6.7
UA	μmol/L	18–65			243	507	178	417	NA			208.3	428.4	154.7	357	18–65			223	444	148	321	20–79			226	458	166	345	18–65			248	509	159	404
Cre	μmol/L	18–65			58	109	45	86	NA			59	104	45	84	18–65			66	111	50	74	20–79			59	92	50	71	18–65			58	95	35	74
TBil	μmol/L	18–65			6	43	5	27	NA	5	21					18–65			3.6	22.4	2.2	15.5	20–79			3.8	24.1	2.7	15.9	18–65			6.2	23.7	4	17.3
Na	mmol/L	18–65	134	142					NA	136	146					18–65	135	144					20–79	137	144					18–65	135	146				
K	mmol/L	18–65	3.4	4.8					NA	3.5	5.1					18–65	3.7	4.9					20–79	3.7	4.9					18–65	3.8	5				
Cl	mmol/L	18–65	100	110					NA	101	109					18–65	101	111					20–79	99	107					18–65	102	113				
Ca	mmol/L	18–65	2.19	2.57					NA	2.2	2.65					18–65	2.11	2.56					20–79	2.15	2.47					18–65	2.1	2.44				
IP	mmol/L	18–65	0.78	1.42					NA	0.81	1.45					18–65	0.81	1.44					20–79	0.8	1.4					18–65	0.8	1.43				
Mg	mmol/L	18–65			0.73	0.98			NA			0.73	1.06	0.77	1.03	18–65	0.71	0.96					20–79	0.77	1.06					18–65	0.77	1.07				
		18–44					0.71	0.93																												
		45–65					0.76	1.01																												
Glu	mmol/L	18–65							NA	4.1	5.9					18–65	4	5.9					20–79	3.96	5.88					18–65						
		18–44	3.9	5.8																										18–45	4.1	5.5				
		45–65	4.4	7.3																										46–65	4.3	6.0				
TC	mmol/L	18–65							NA		5.2					18–65	3.5	6.36												18–65					2.9	6.6
		18–45	3.10	6.10																			20–49	3.22	6.45	3.20	6.42	3.20	6.38	18–45			3.1	6.2		
		46–65	3.20	7.20																			50–79					3.93	7.92	46–65			2.5	6.7		
TG	mmol/L	18–65							NA		1.7					18–65			0.5	3.58	0.39	1.6								18–65			0.6	2.7	0.5	2.1
		18–45			0.50	2.88	0.45	2.03															20–49			0.53	3.39	0.46	2.52	18–45						
		46–65			0.53	3.87	0.55	3.24															50–79					0.64	3.55	46–65						
HDL-C	mmol/L	18–65	0.70	1.70					NA	1.03	1.55					18–65			0.74	1.76	0.98	2.19				0.85	1.52	0.95	1.56	18–65			0.7	1.5	0.8	1.8
LDL-C	mmol/L	18–65			1.60	4.80			NA		2.60					18–65	1.8	4.34												18–65	1.7	4.4				
		18–44					1.50	4.20															20–49	1.47	3.92	1.60	4.01	1.32	3.92	18–45						
		45–65					1.70	4.90															50–79					1.78	4.91	46–65						
Lip	U/L	18–65			8	75	10	63	NA		67																			18–65						
ALT	IU/L	18–65			10	55	7	30	NA				50		35	18–65			7	39	5	18				9	57	7	28	18–65			15	74	10	37
AST	IU/L	18–65			17	40	15	29	NA				50		35	18–65			11	28	10	24				13	30	11	25	18–65			20	53	17	39
ALP	IU/L	18–65			53	153			NA	30	120						39	114												18–65			41	111		
		18–44					47	130															20–49	38	112	43	116	34	97	18–45					35	100
		45–65					55	174															50–79					47	133	46–65					43	117
AMY	IU/L	18–65	47	164					NA	22	80					18–65	31	117						34	119					18–65	36	135				
LDH	IU/L	18–65	138	257					NA				248		247	18–65	10	238						126	220					18–65	104	206				
CK	IU/L	18–65			72	460	53	260	NA				171		145	18–65			54	266	27	138				48	227	34	131	18–65			48	304	36	184
GGT	IU/L	18–65			13	90	10	47	NA				55		38	18–65			11	65	7	21				11	69	7	33	18–65			14	62	11	40
Fe	μmol/L	18–65	8.8	28.9					NA			12.5	32.2	10.7	32.2	18–65			7.9	29.6	3.7	26				5.9	31.6	3.5	27.8	18–65			7	33	4	26
Ferr	μg/L	18–65			20	457			NA			20	250	10	120								18–79					4.7	136	18–65						
		18–44					5	150															18–44			13	276	4.3	91							
		45–65					12	232															45–79					5.9	175							
Tf	g/L	18–65			1.9	3.2	2.0	3.5	NA	2	3.6					18–65			2	3.2	2.1	3.9	18–79	1.8	3.3	1.8	3.3	1.9	3.5	18–65	2.2	4				
TfSat	%	18–65	13	47																										18–65						
CRP	mg/L	18–65	0.21	14.7					NA		1					18–65	0.2	11.8												18–65			0.33	7.34	0.35	11.9
IgA	g/L	18–65	1.05	4.63					NA	0.7	4																			18–65	0.94	4.35				
IgG	g/L	18–65	11.1	20.0					NA	7	16																			18–65	9.1	20.4				
IgM	g/L	18–65			0.38	2.29	0.51	2.77	NA	0.4	2.3																			18–65			0.4	2.54	0.51	3.1
FT3	pmol/L	18–65	3.9	6.3					NA	3.8	6																			18–65						
FT4	pmol/L	18–65	7.8	14.1					NA	7.86	14.41																			18–65						
TSH	mIU/L	18–65	0.61	4.86					NA	0.38	5.33																			18–65						
PSA	μg/L	18–65			0.25	2.95																								18–65						

### Standardization of the RIs

Since this study utilized a serum panel provided by C-RIDL which comprised 50 samples with values assigned to 25 chemistry analytes, we confirmed the standardized status of the assays as shown in S4 Fig. For the method comparison, major-axis regression was used to express the structural relationship between our test results and the panel assigned values by calculating BR_LL_ or BR_UL_ as a difference of LL or UL before and after the recalibration using the regression coefficients. As a result, we found it necessary to recalibrate our RIs for HDL-C and LDH. For Na, we could not get a good linear relationship because of poor precision of the assay with the narrow reference interval.

## Discussion

There have been controversies over the statistical methods used in deriving RIs. They include: selection between parametric and nonparametric methods, how and when to exclude RVs secondarily, and when to partition RVs into subgroups by sex and age. In this study we sought optimal options for each. We found the nonparametric method of limited use with its wider 90% CI for RIs and frequent bias in UL (S1 Fig), while the parametric method was found to be more reliable after successful Gaussian transformation (S3 Fig). For secondary exclusion, we found LAVE procedure effective for reducing the influence of over nutrition for those analytes with high association with BMI such as TG, ALT, AST, and CRP. It was also effective in reducing the influence of latent inflammation and anemia on Fe, Tf and TfSat.

In order to decide the need for partitioning RVs into subgroups by sex or age, we primarily used SDR, but it tended to provide an inflated value when the width of the RI was narrow. Another problem of SDR is that it reflects between-subgroup difference at the central part which may not reflect between-subgroup bias at the LL or UL, hence the use of BR_LL_ or BR_UL_. Furthermore, we found it necessary to confirm the appropriateness of BR_LL_ or BR_UL_ by quantitating the actual difference at LL or UL by use of the reporting unit (RU). We chose to adopt partitioned RIs only when the difference at LL or UL was ≥3RU. We believe this three-way consideration ensured optimal judgement in partitioning RIs by sex and age as shown in S1 Table.

RIs can vary appreciably across different populations as demonstrated in the interim report of the global study analyzing RVs of 12 countries [4]. They identified ethnic differences in many analytes such as Alb, urea, TBil, HDL-C, CRP, IgG, C3, and PTH. In reference to the RV profiles, we noted certain unique features of Kenyan RIs. For instance, our urea RIs are lower than those from Turkey and Saudi Arabia. Although most of our RIs for liver function tests were similar to published reports from Saudi Arabia and Turkey [12,13], our TBil RI of 6–43 μmol/L for males and 5–27 μmol/L for females is almost double what has been reported in published studies from outside the African continent as shown in S2 Table [2,12–15]. We hypothesize that Gilbert’s syndrome, the commonest genetic cause of asymptomatic unconjugated hyperbilirubinaemia, could be quite prevalent in our population. Genetic studies would thus be useful in elucidating the cause of hyperbilirubinaemia observed in our study.

Our electrolytes didn’t differ much from other published IFCC studies except that Mg levels increased with age in females. It has been documented that reduced levels of oestrogen are associated with increased Mg levels in post-menopausal women as well as in the follicular phase of the menstrual cycle in women of reproductive age [16,17].

Our TC and TG RIs were higher than those reported by Kibaya *et al* who carried out a similar study in a rural Kenyan population [18]. Our study population was primarily composed of urban dwellers of whom 25.6% had metabolic syndrome [19]. According to the third report of the National Cholesterol Education Program (NCEP), the desirable LDL-C level for low risk adults is <4.1 mmol/L [20]. Our UL for LDL-C in males (4.8 mmol/L) and in females (4.2 and 4.9 mmol/L) are higher than the NCEP targets. However, the NCEP clinical decision limits (CDLs) are used for diagnosis of hyperlipidaemia and serve as treatment targets for reducing cardiovascular risk. Aside from the risk of over-nutrition, the RI ULs for TC and LDL-C are important in diagnosing cholestatic conditions and hypothyroidism. These are relatively short-term conditions unrelated to occurrence of atherosclerosis hence the RIs are more relevant than CDLs in their diagnosis. On the other hand, determination of LLs for TC or LDL-C RIs are essential for diagnosing malnutrition or thyrotoxicosis. Therefore, we are not replacing the derived RIs with those CDLs, rather providing CDLs in the footnote of test result report sheet.

We obtained fasting plasma glucose (FPG) RIs of 3.9−5.8 and 4.4−7.3 mmol/L for those aged <45 and ≥45 years respectively. The former RI is comparable to what was obtained in Turkey (3.96−5.88 mmol/L) and Saudi Arabia (4.0−5.9 mmol/L) [12,13]. The American Diabetes Association (ADA) uses FPG ≥ 5.6 and 7.0 mmol/L to define pre-diabetes and diabetes respectively. Based on the ADA criteria, a total of 63 out of 528 participants would have been classified as having elevated values compared to 20 if the ULs of the derived RIs were used.

Our RIs for immunoglobulins are higher than those derived in the US but very similar to those derived in India [2,15]. Karita *et al* also found high levels of IgG in several SSA countries [21]. We hypothesize that this may be due to inflammation caused by increased exposure to either infectious disease agents or environmental allergens. The increased inflammation is further evidenced by the very high RI UL for CRP of 14.7 mg/L compared to 1 mg/L recommended by Beckman Coulter [22]. Ichihara *et al* in a similar study carried out in Asia found that the closer the country or region was to the equator, the higher the serum concentrations of positive inflammatory markers (IgG, C3, CRP) a phenomenon they ascribed to increased exposure to infectious agents [23]. Similar to the IFCC study in India, IgM was significantly higher in female participants requiring the determination of sex specific RIs [15]. We hypothesize that this could be linked to the role that estrogen plays in enhancing humoral immunity [24].

Ichihara *et al* has demonstrated that BMI is a major source of variation in RVs for many analytes and that the magnitude of this association varies across different populations [25]. For example, a similar change in BMI resulted in a greater decline in HDL cholesterol amongst the Japanese (r = −0.39) compared to people from Pakistan (r = −0.05). On the other hand, an increase in BMI was associated with a greater increase in ALT values (r = 0.48) amongst non-black South Africans compared to black South Africans (r = 0.02) [25]. BMI was a source of variation for several analytes in males especially those known to be associated with the presence of metabolic syndrome such as Glu, LDL-C, and ALT. We did not partition our RIs by BMI because the influence of BMI was suppressed by use of the LAVE method.

For iron markers, we also applied LAVE to reduce the influence of latent anemia. Although it was very effective in raising their LLs, the female LL for ferritin was still lower than the WHO cutoff value of <15 μg/L for iron deficiency [26].

The strengths of our study include the deliberate recruitment of healthy individuals using a harmonized protocol to ensure pre-analytical confounders were minimized, centralized analysis of samples in an accredited laboratory with excellent internal quality control, standardization of the RIs by use of a value-assigned panel of sera and use of the LAVE procedure to reduce the influence of sub-clinical disease on the derived RIs.

The weaknesses include failure to perform infectious serology to rule out chronic infections such as HIV, HBV or HCV, and over representation of an urban population hence limiting the generalizability of our findings to a rural population.

## Conclusion

According to the harmonized IFCC-C-RIDL protocol, we established RIs for 40 major chemistry and immunoassay tests from well-defined healthy Kenyan volunteers by use of the up-to-date statistical methods for the first time in Africa. The LAVE method was effective in reducing the influence on RIs of latent anemia and metabolic disorders. Based on SD ratio and bias ratios, we developed a flow chart to judge the need for partitioning RVs by sex and age subgroups, which we believe is helpful for the future RI study. RIs for chemistry analytes were standardized by use of a value assigned serum panel, and thus could be shared across sub-Saharan African laboratories with similar ethnic and life-style profile. As a whole, Kenyan RIs were comparable to those of other countries participating in the global study with a few exceptions such as higher ULs for TBil and CRP.

## Supporting information

S1 Fig Sex and age-related changes. The distributions of reference values are shown based on age and sex stratification for all analytes. The SDRsex and SDRage for each sex are shown at the top of each analyte chart. No secondary exclusion was performed in plotting the data. The box in each scattergram represents central 50% range and the vertical bar in the middle represents a median point.
(PDF)

S2 Fig Bar-chart comparison of reference interval calculation methods. The RIs of all analytes were derived in four ways by parametric (P) or nonparametric (NP) method with/without the LAVE procedure, separately in males+Females (MF), males, and females. Each horizontal bar represents the range of the RI, and the vertical line in the center corresponds to the mid-point. The shades on both ends of the bar represent 90%CI for the limits of the RI predicted by the bootstrap method.
(PDF)

S3 Fig Results of Gaussian transformation by the parametric method. The accuracy of Gaussian transformation by Box-Cox formula can be assessed from theoretical Gaussian curves in two histograms shown on left top (before and after the transformation) of each panel. Accuracy can be also seen from the linearity in probability paper plot on the right. The limits of the RI by nonparametric method corresponds to the points where red zigzag line intersect with horizontal 2.5 and 97.5% red lines of cumulative frequencies.
(PDF)

S4 Fig Panel test results for assessment of standardized status of assays. The panel of sera from 50 healthy volunteers, each of which were value assigned for 40 chemistry analytes were measured. Our measured values were plotted on Y-axis and assigned values on X-axis. Major axis linear regression was used as a structural relationship for the method comparison. The Y = X line is shown as a diagonal broken line.
(PDF)

S1 Table Reference intervals and determination of method, sex and age bias. (PDF)

S2 Table Comparison of reference intervals. (PDF)
