# Correction: Determination of reference intervals for common chemistry and immunoassay tests for Kenyan adults based on an internationally harmonized protocol and up-to-date statistical methods

**DOI:** 10.1371/journal.pone.0315969

**Published:** 2024-12-12

**Authors:** Geoffrey Omuse, Kiyoshi Ichihara, Daniel Maina, Mariza Hoffman, Elizabeth Kagotho, Alice Kanyua, Jane Mwangi, Caroline Wambua, Angela Amayo, Peter Ojwang, Zul Premji, Rajiv Erasmus

Fig 1 is incorrect. The yellow box with a comment should be removed. The authors have provided a corrected version here.

**Fig 1:**
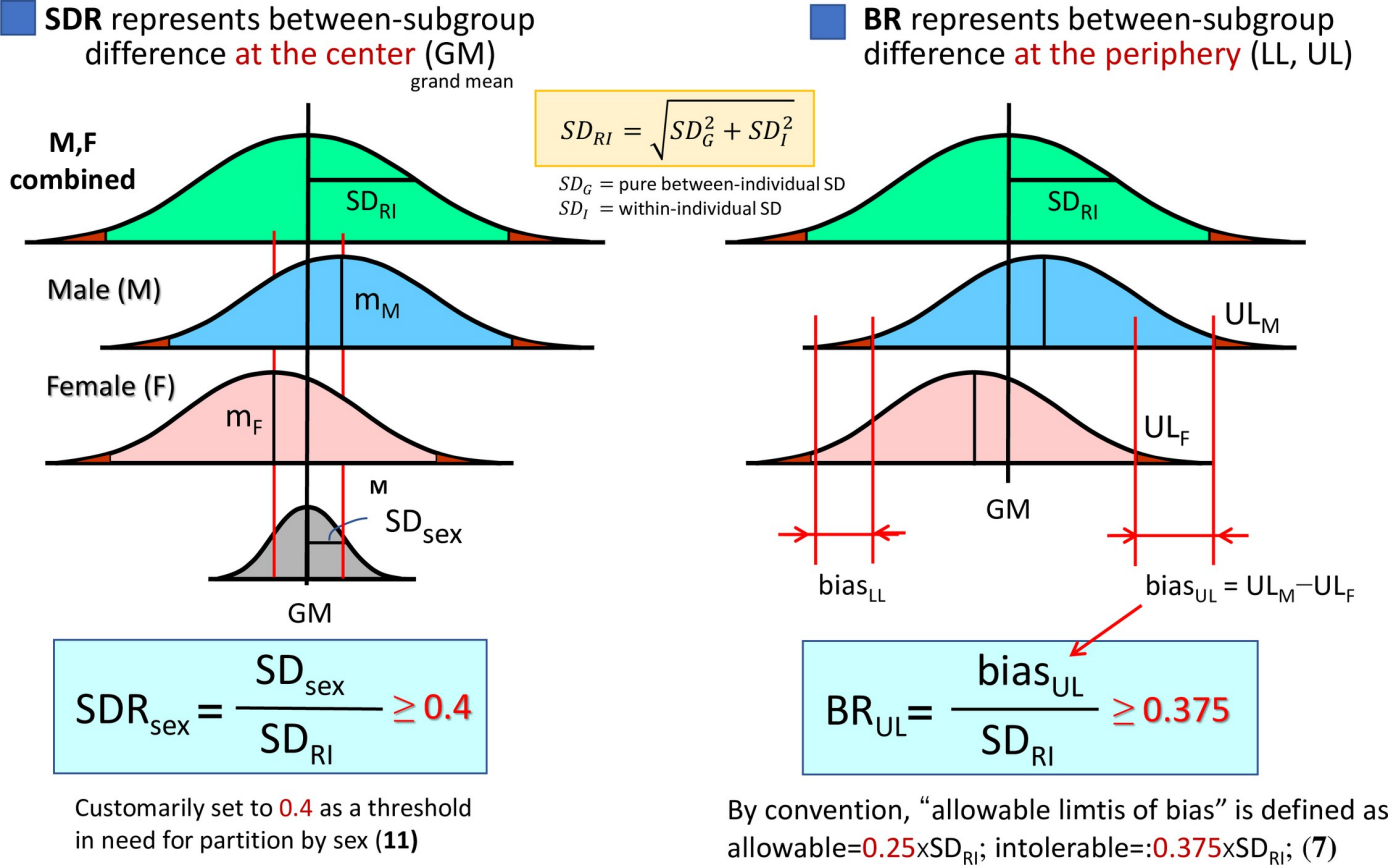
SD ratio (SDR) vs. bias ratio (BR) as a measure of between-subgroup differences. **SDR** represents between-subgroup differences at the center of distributions, while **BR** represents between-subgroup differences at the periphery (LL or UL) of the distributions. The numerator of SDR is between-subgroup SD (or SDsex, if sub-grouped by sex), while that of BR is a difference of LLs or ULs.
