# Supplementary material for: Determination of reference intervals for common chemistry and immunoassay tests for Kenyan adults based on an internationally harmonized protocol and up-to-date statistical methods
Source: PLoS One. 2020 Jul 9;15(7):e0235234. doi: 10.1371/journal.pone.0235234 (PMC7347104; doi:10.1371/journal.pone.0235234)
Supplement: S1 Fig — The distributions of reference values are shown based on age and sex stratification for all analytes. The SDRsex and SDRage for each sex are shown at the top of each analyte chart. No secondary exclusion was performed in plotting the data. The box in each scattergram represents central 50% range and the vertical bar in the middle represents a median point. (PDF) [file pone.0235234.s001.pdf]

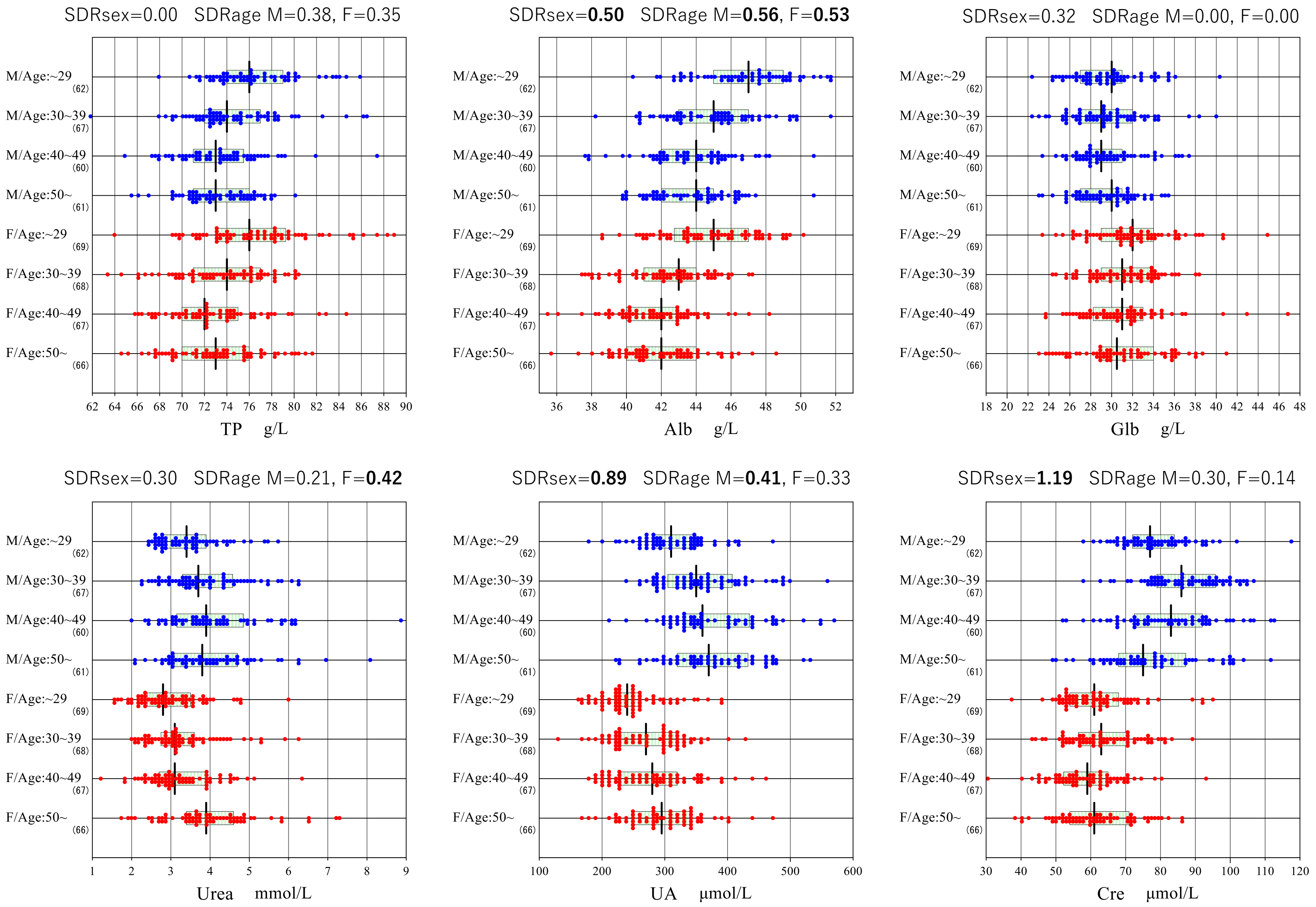

SDRsex=0.00 SDRage M=0.38, F=0.35
SDRsex=0.50 SDRage M=0.56, F=0.53
SDRsex=0.32 SDRage M=0.00, F=0.00
M/Age:~29 (62)
M/Age:30~39 (67)
M/Age:40~49 (60)
M/Age:50~ (61)
F/Age:~29 (69)
F/Age:30~39 (68)
F/Age:40~49 (67)
F/Age:50~ (66)
62 64 66 68 70 72 74 76 78 80 82 84 86 88 90
TP g/L
36 38 40 42 44 46 48 50 52
Alb g/L
18 20 22 24 26 28 30 32 34 36 38 40 42 44 46 48
Glb g/L
SDRsex=0.30 SDRage M=0.21, F=0.42
SDRsex=0.89 SDRage M=0.41, F=0.33
SDRsex=1.19 SDRage M=0.30, F=0.14
1 2 3 4 5 6 7 8 9
Urea mmol/L
100 200 300 400 500 600
UA μmol/L
30 40 50 60 70 80 90 100 110 120
Cre μmol/L

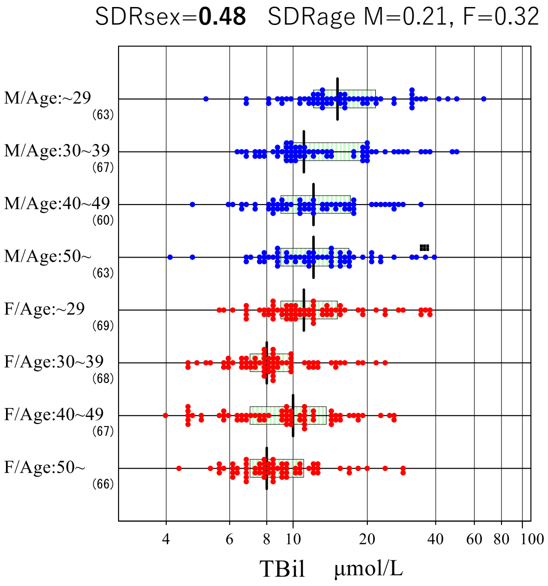
SDRsex=0.48 SDRage M=0.21, F=0.32
M/Age:~29 (63)
M/Age:30~39 (67)
M/Age:40~49 (60)
M/Age:50~ (63)
F/Age:~29 (69)
F/Age:30~39 (68)
F/Age:40~49 (67)
F/Age:50~ (66)
4 6 8 10 20 40 60 80 100
TBil μmol/L

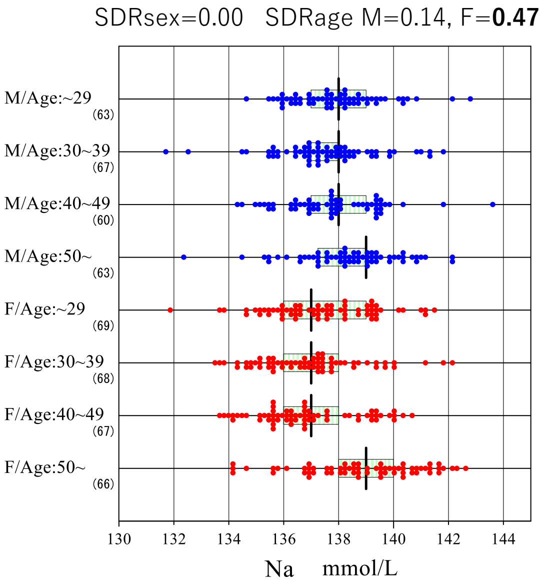
SDRsex=0.00 SDRage M=0.14, F=0.47
M/Age:~29 (63)
M/Age:30~39 (67)
M/Age:40~49 (60)
M/Age:50~ (63)
F/Age:~29 (69)
F/Age:30~39 (68)
F/Age:40~49 (67)
F/Age:50~ (66)
130 132 134 136 138 140 142 144
Na mmol/L

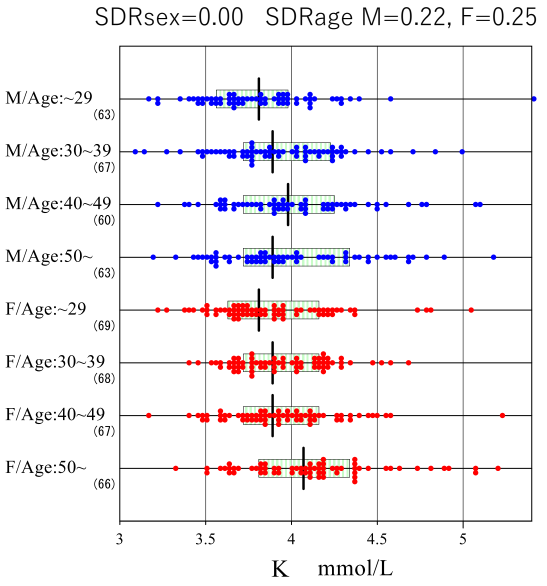
SDRsex=0.00 SDRage M=0.22, F=0.25
M/Age:~29 (63)
M/Age:30~39 (67)
M/Age:40~49 (60)
M/Age:50~ (63)
F/Age:~29 (69)
F/Age:30~39 (68)
F/Age:40~49 (67)
F/Age:50~ (66)
3 3.5 4 4.5 5
K mmol/L

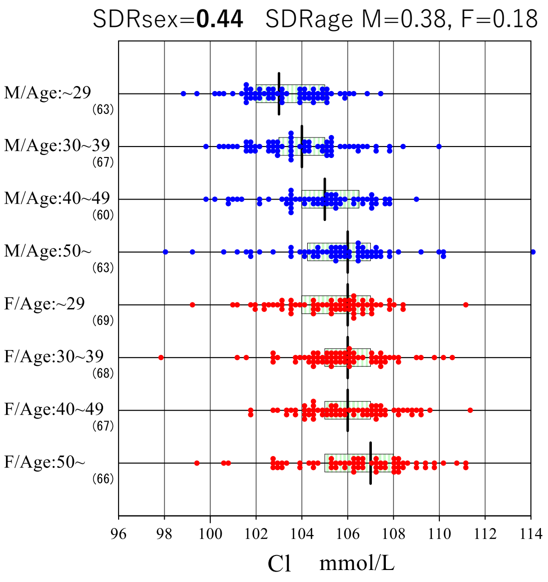
SDRsex=0.44 SDRage M=0.38, F=0.18
M/Age:~29 (63)
M/Age:30~39 (67)
M/Age:40~49 (60)
M/Age:50~ (63)
F/Age:~29 (69)
F/Age:30~39 (68)
F/Age:40~49 (67)
F/Age:50~ (66)
96 98 100 102 104 106 108 110 112 114
Cl mmol/L

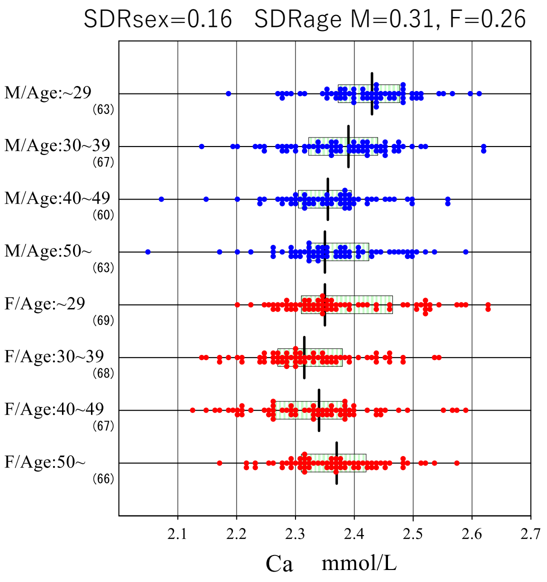
SDRsex=0.16 SDRage M=0.31, F=0.26
M/Age:~29 (63)
M/Age:30~39 (67)
M/Age:40~49 (60)
M/Age:50~ (63)
F/Age:~29 (69)
F/Age:30~39 (68)
F/Age:40~49 (67)
F/Age:50~ (66)
2.1 2.2 2.3 2.4 2.5 2.6 2.7
Ca mmol/L

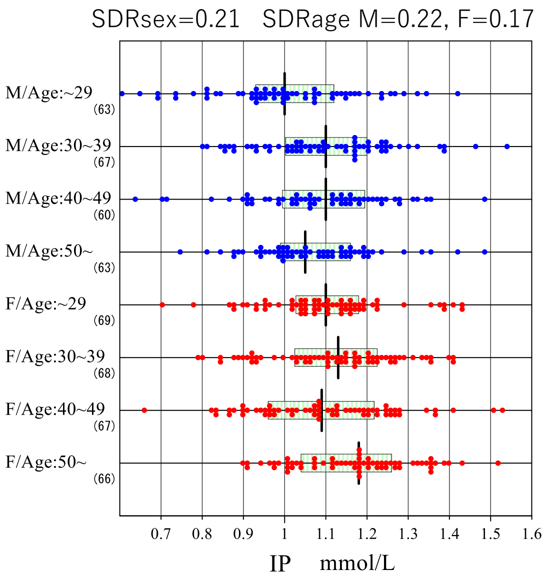
SDRsex=0.21 SDRage M=0.22, F=0.17
M/Age:~29 (63)
M/Age:30~39 (67)
M/Age:40~49 (60)
M/Age:50~ (63)
F/Age:~29 (69)
F/Age:30~39 (68)
F/Age:40~49 (67)
F/Age:50~ (66)
0.7 0.8 0.9 1 1.1 1.2 1.3 1.4 1.5 1.6
IP mmol/L

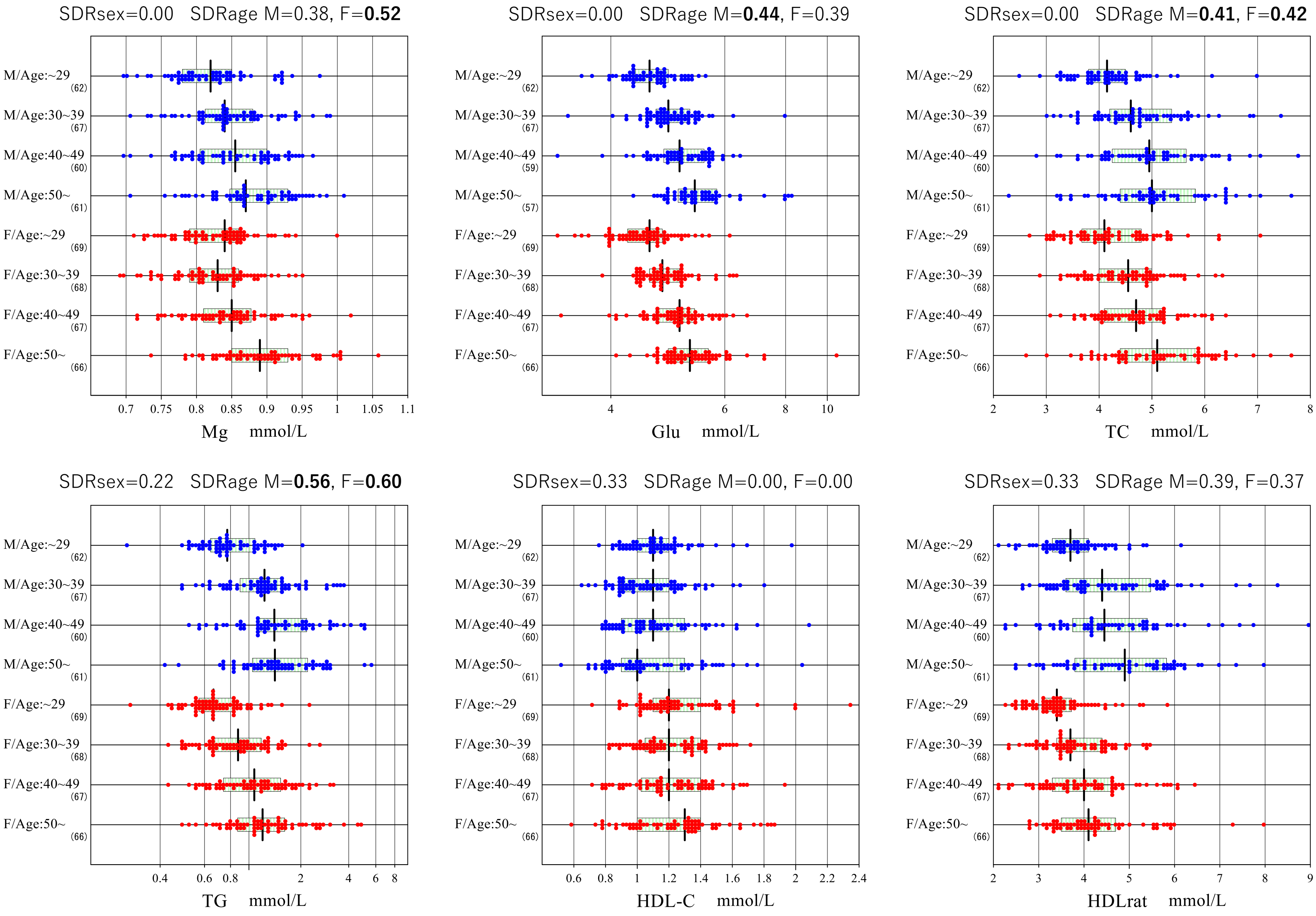
SDRsex=0.00 SDRage M=0.38, F=**0.52**
M/Age:~29 (62)
M/Age:30~39 (67)
M/Age:40~49 (60)
M/Age:50~ (61)
F/Age:~29 (69)
F/Age:30~39 (68)
F/Age:40~49 (67)
F/Age:50~ (66)
0.7 0.75 0.8 0.85 0.9 0.95 1 1.05 1.1
Mg mmol/L
SDRsex=0.00 SDRage M=**0.44**, F=0.39
M/Age:~29 (62)
M/Age:30~39 (67)
M/Age:40~49 (59)
M/Age:50~ (57)
F/Age:~29 (69)
F/Age:30~39 (68)
F/Age:40~49 (67)
F/Age:50~ (66)
4 6 8 10
Glu mmol/L
SDRsex=0.00 SDRage M=**0.41**, F=**0.42**
M/Age:~29 (62)
M/Age:30~39 (67)
M/Age:40~49 (60)
M/Age:50~ (61)
F/Age:~29 (69)
F/Age:30~39 (68)
F/Age:40~49 (67)
F/Age:50~ (66)
2 3 4 5 6 7 8
TC mmol/L
SDRsex=0.22 SDRage M=**0.56**, F=**0.60**
M/Age:~29 (62)
M/Age:30~39 (67)
M/Age:40~49 (60)
M/Age:50~ (61)
F/Age:~29 (69)
F/Age:30~39 (68)
F/Age:40~49 (67)
F/Age:50~ (66)
0.4 0.6 0.8 2 4 6 8
TG mmol/L
SDRsex=0.33 SDRage M=0.00, F=0.00
M/Age:~29 (62)
M/Age:30~39 (67)
M/Age:40~49 (60)
M/Age:50~ (61)
F/Age:~29 (69)
F/Age:30~39 (68)
F/Age:40~49 (67)
F/Age:50~ (66)
0.6 0.8 1 1.2 1.4 1.6 1.8 2 2.2 2.4
HDL-C mmol/L
SDRsex=0.33 SDRage M=0.39, F=0.37
M/Age:~29 (62)
M/Age:30~39 (67)
M/Age:40~49 (60)
M/Age:50~ (61)
F/Age:~29 (69)
F/Age:30~39 (68)
F/Age:40~49 (67)
F/Age:50~ (66)
2 3 4 5 6 7 8 9
HDLrat mmol/L

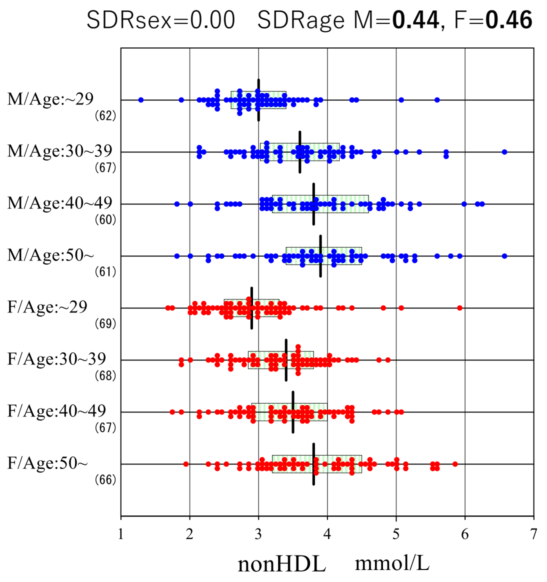
SDRsex=0.00 SDRage M=**0.44**, F=**0.46**
M/Age:~29 (62)
M/Age:30~39 (67)
M/Age:40~49 (60)
M/Age:50~ (61)
F/Age:~29 (69)
F/Age:30~39 (68)
F/Age:40~49 (67)
F/Age:50~ (66)
1 2 3 4 5 6 7
nonHDL mmol/L

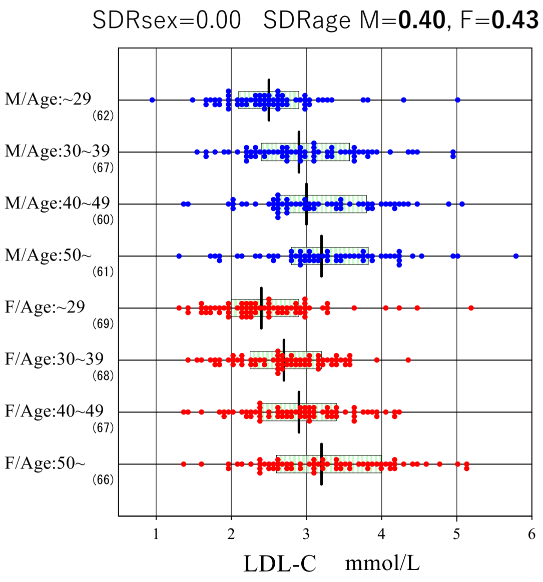
SDRsex=0.00 SDRage M=**0.40**, F=**0.43**
M/Age:~29 (62)
M/Age:30~39 (67)
M/Age:40~49 (60)
M/Age:50~ (61)
F/Age:~29 (69)
F/Age:30~39 (68)
F/Age:40~49 (67)
F/Age:50~ (66)
1 2 3 4 5 6
LDL-C mmol/L

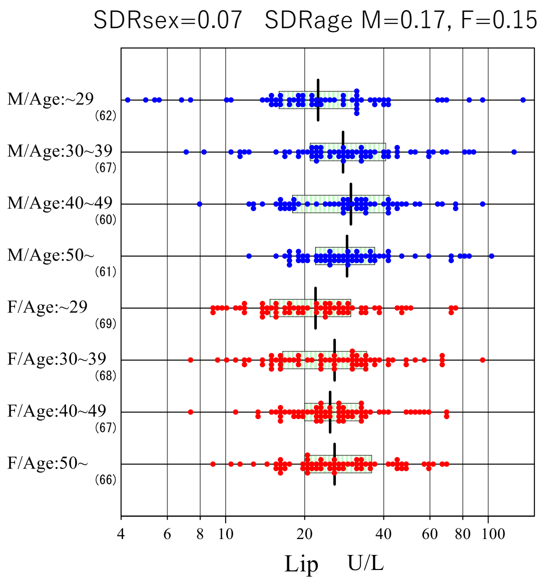
SDRsex=0.07 SDRage M=0.17, F=0.15
M/Age:~29 (62)
M/Age:30~39 (67)
M/Age:40~49 (60)
M/Age:50~ (61)
F/Age:~29 (69)
F/Age:30~39 (68)
F/Age:40~49 (67)
F/Age:50~ (66)
4 6 8 10 20 40 60 80 100
Lip U/L

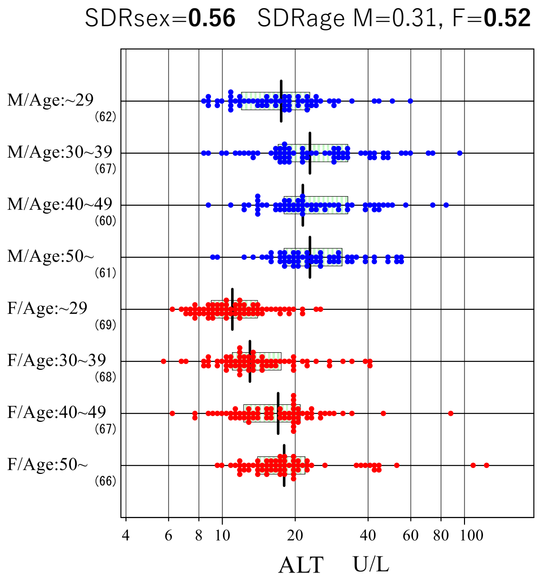
SDRsex=**0.56** SDRage M=0.31, F=**0.52**
M/Age:~29 (62)
M/Age:30~39 (67)
M/Age:40~49 (60)
M/Age:50~ (61)
F/Age:~29 (69)
F/Age:30~39 (68)
F/Age:40~49 (67)
F/Age:50~ (66)
4 6 8 10 20 40 60 80 100
ALT U/L

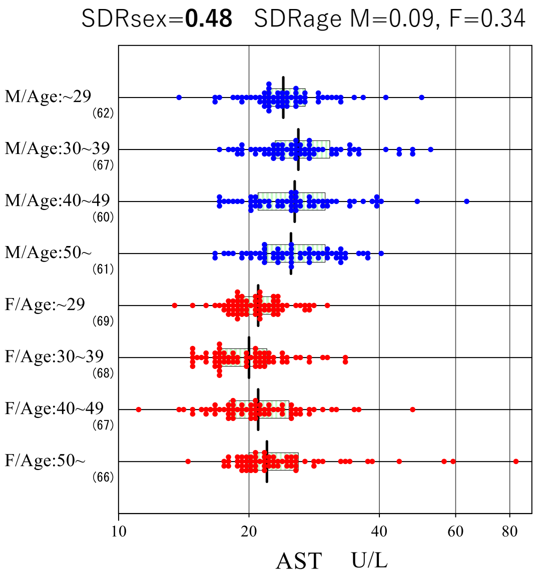
SDRsex=**0.48** SDRage M=0.09, F=0.34
M/Age:~29 (62)
M/Age:30~39 (67)
M/Age:40~49 (60)
M/Age:50~ (61)
F/Age:~29 (69)
F/Age:30~39 (68)
F/Age:40~49 (67)
F/Age:50~ (66)
10 20 40 60 80
AST U/L

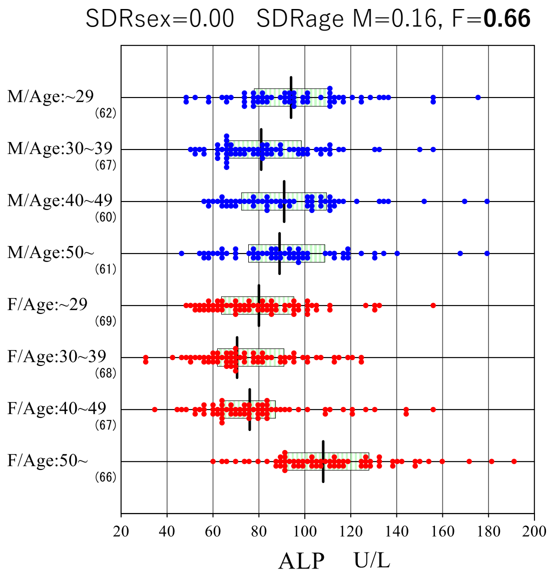
SDRsex=0.00 SDRage M=0.16, F=**0.66**
M/Age:~29 (62)
M/Age:30~39 (67)
M/Age:40~49 (60)
M/Age:50~ (61)
F/Age:~29 (69)
F/Age:30~39 (68)
F/Age:40~49 (67)
F/Age:50~ (66)
20 40 60 80 100 120 140 160 180 200
ALP U/L

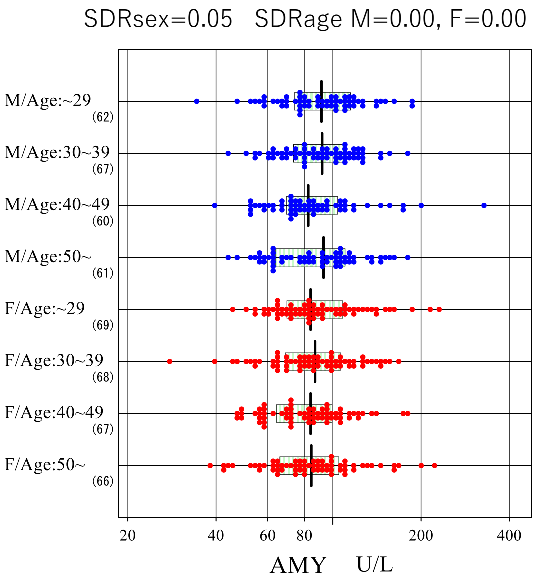
SDRsex=0.05 SDRage M=0.00, F=0.00
M/Age:~29 (62)
M/Age:30~39 (67)
M/Age:40~49 (60)
M/Age:50~ (61)
F/Age:~29 (69)
F/Age:30~39 (68)
F/Age:40~49 (67)
F/Age:50~ (66)
20 40 60 80 200 400
AMY U/L

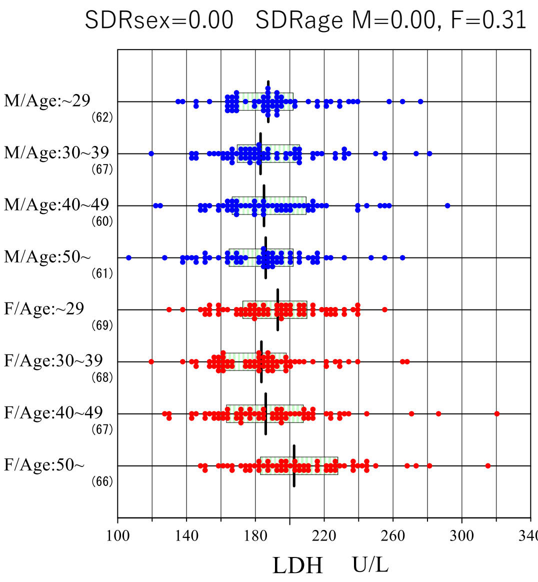
SDRsex=0.00 SDRage M=0.00, F=0.31
M/Age:~29 (62)
M/Age:30~39 (67)
M/Age:40~49 (60)
M/Age:50~ (61)
F/Age:~29 (69)
F/Age:30~39 (68)
F/Age:40~49 (67)
F/Age:50~ (66)
100 140 180 220 260 300 340
LDH U/L

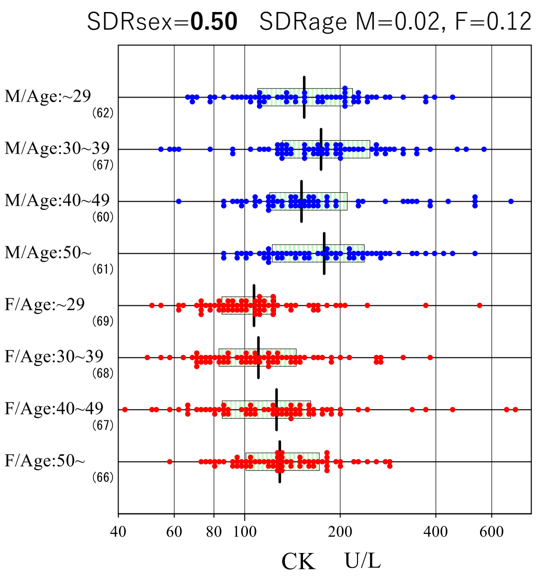
SDRsex=0.50 SDRage M=0.02, F=0.12
M/Age:~29 (62)
M/Age:30~39 (67)
M/Age:40~49 (60)
M/Age:50~ (61)
F/Age:~29 (69)
F/Age:30~39 (68)
F/Age:40~49 (67)
F/Age:50~ (66)
40 60 80 100 200 400 600
CK U/L

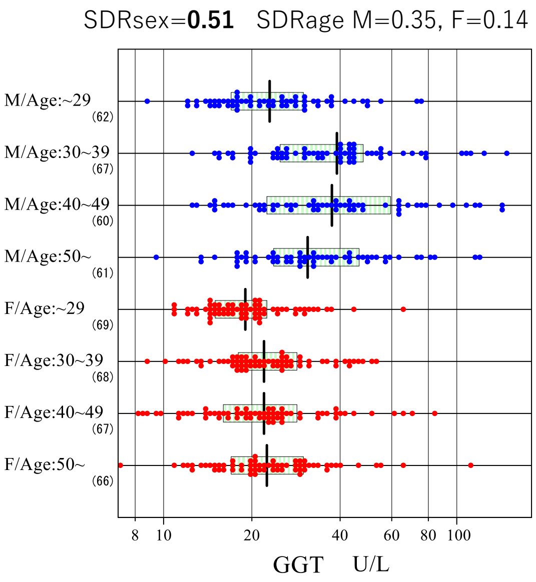
SDRsex=0.51 SDRage M=0.35, F=0.14
M/Age:~29 (62)
M/Age:30~39 (67)
M/Age:40~49 (60)
M/Age:50~ (61)
F/Age:~29 (69)
F/Age:30~39 (68)
F/Age:40~49 (67)
F/Age:50~ (66)
8 10 20 40 60 80 100
GGT U/L

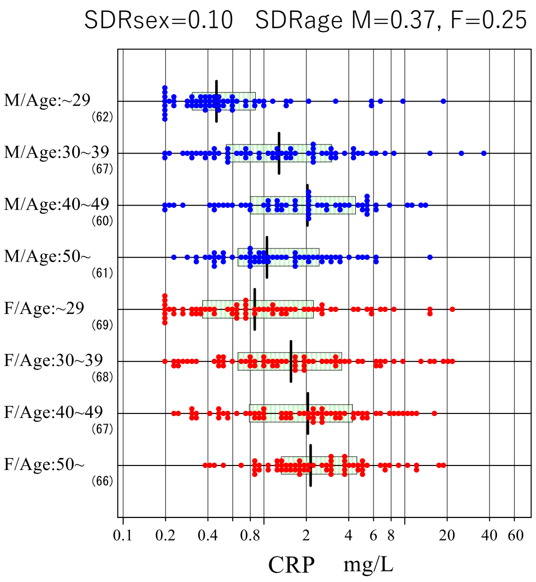
SDRsex=0.10 SDRage M=0.37, F=0.25
M/Age:~29 (62)
M/Age:30~39 (67)
M/Age:40~49 (60)
M/Age:50~ (61)
F/Age:~29 (69)
F/Age:30~39 (68)
F/Age:40~49 (67)
F/Age:50~ (66)
0.1 0.2 0.4 0.8 2 4 6 8 20 40 60
CRP mg/L

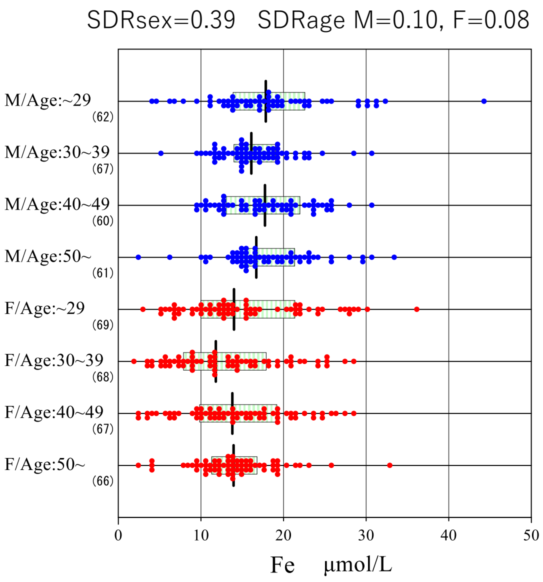
SDRsex=0.39 SDRage M=0.10, F=0.08
M/Age:~29 (62)
M/Age:30~39 (67)
M/Age:40~49 (60)
M/Age:50~ (61)
F/Age:~29 (69)
F/Age:30~39 (68)
F/Age:40~49 (67)
F/Age:50~ (66)
0 10 20 30 40 50
Fe μmol/L

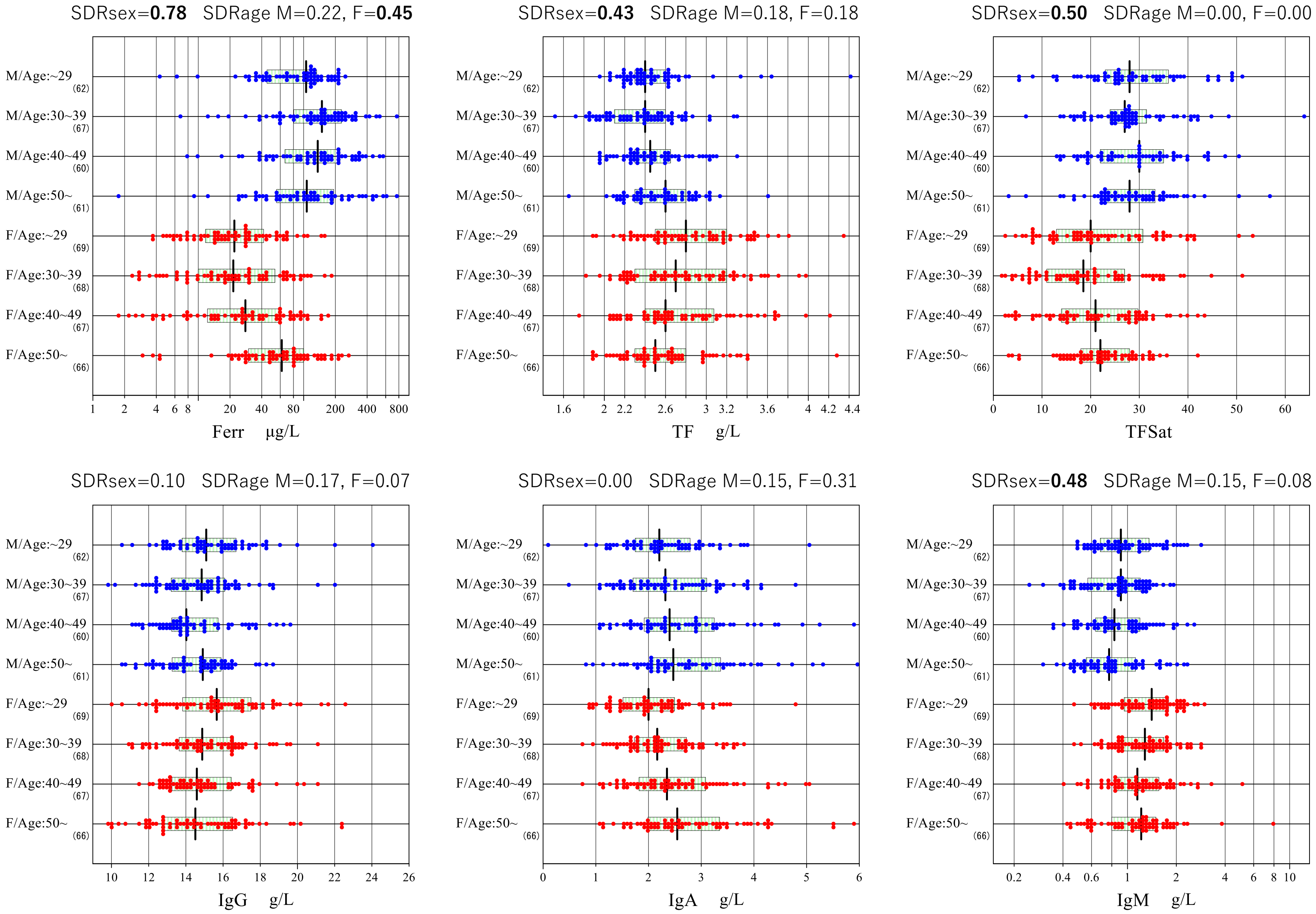
SDRsex=0.78 SDRage M=0.22, F=0.45
SDRsex=0.43 SDRage M=0.18, F=0.18
SDRsex=0.50 SDRage M=0.00, F=0.00
M/Age:~29 (62)
M/Age:30~39 (67)
M/Age:40~49 (60)
M/Age:50~ (61)
F/Age:~29 (69)
F/Age:30~39 (68)
F/Age:40~49 (67)
F/Age:50~ (66)
1 2 4 6 8 20 40 80 200 400 800
Ferr µg/L
1.6 2 2.2 2.6 3 3.2 3.6 4 4.2 4.4
TF g/L
0 10 20 30 40 50 60
TFSat
SDRsex=0.10 SDRage M=0.17, F=0.07
SDRsex=0.00 SDRage M=0.15, F=0.31
SDRsex=0.48 SDRage M=0.15, F=0.08
10 12 14 16 18 20 22 24 26
IgG g/L
0 1 2 3 4 5 6
IgA g/L
0.2 0.4 0.6 1 2 4 6 8 10
IgM g/L

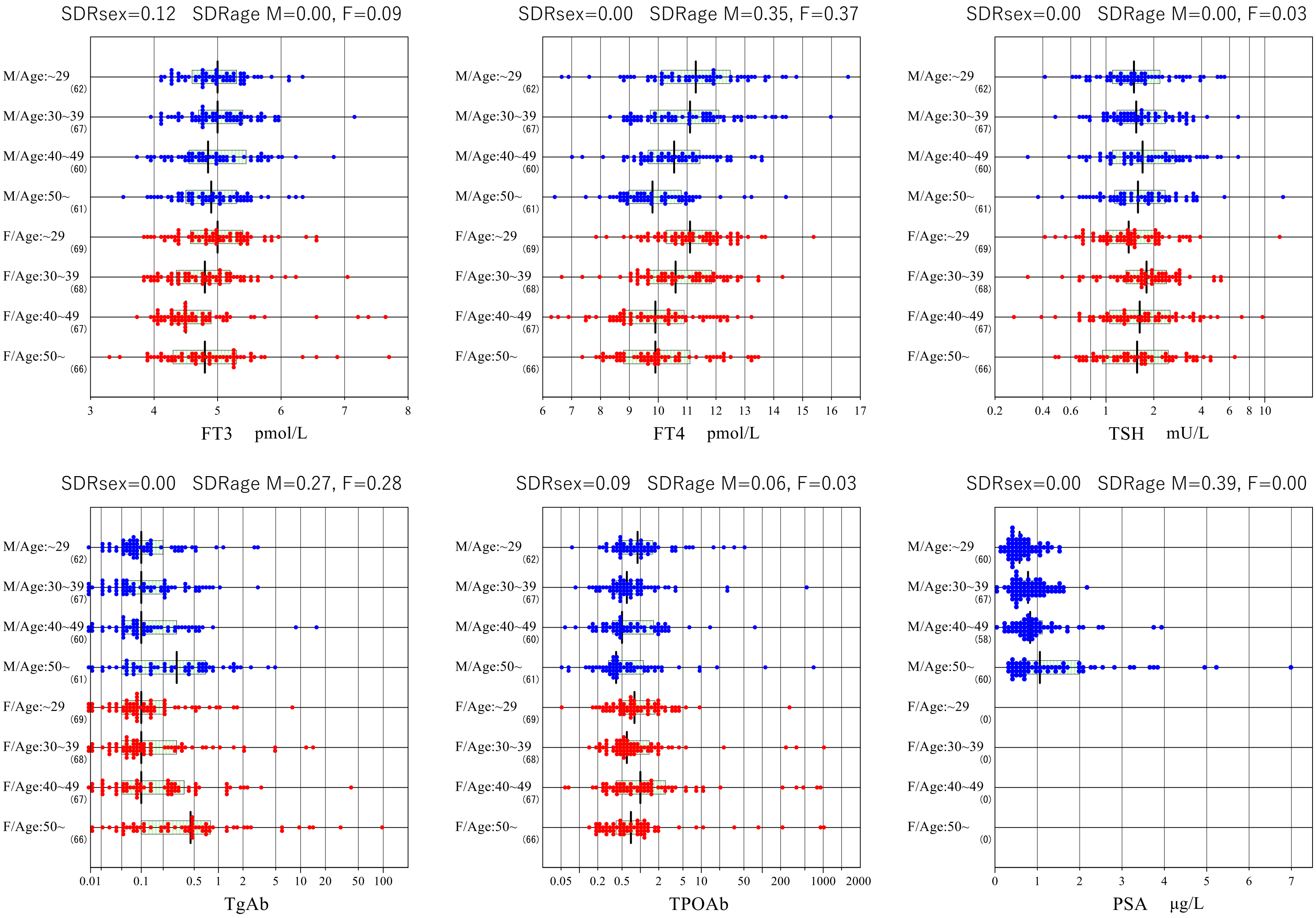

SDRsex=0.12 SDRage M=0.00, F=0.09
SDRsex=0.00 SDRage M=0.35, F=0.37
SDRsex=0.00 SDRage M=0.00, F=0.03
M/Age:~29 (62)
M/Age:30~39 (67)
M/Age:40~49 (60)
M/Age:50~ (61)
F/Age:~29 (69)
F/Age:30~39 (68)
F/Age:40~49 (67)
F/Age:50~ (66)
3 4 5 6 7 8
FT3 pmol/L
6 7 8 9 10 11 12 13 14 15 16 17
FT4 pmol/L
0.2 0.4 0.6 1 2 4 6 8 10
TSH mU/L
SDRsex=0.00 SDRage M=0.27, F=0.28
SDRsex=0.09 SDRage M=0.06, F=0.03
SDRsex=0.00 SDRage M=0.39, F=0.00
0.01 0.1 0.5 1 2 5 10 20 50 100
TgAb
0.05 0.2 0.5 2 5 10 50 200 1000 2000
TPOAb
M/Age:~29 (60)
M/Age:30~39 (67)
M/Age:40~49 (58)
M/Age:50~ (60)
F/Age:~29 (0)
F/Age:30~39 (0)
F/Age:40~49 (0)
F/Age:50~ (0)
0 1 2 3 4 5 6 7
PSA μg/L

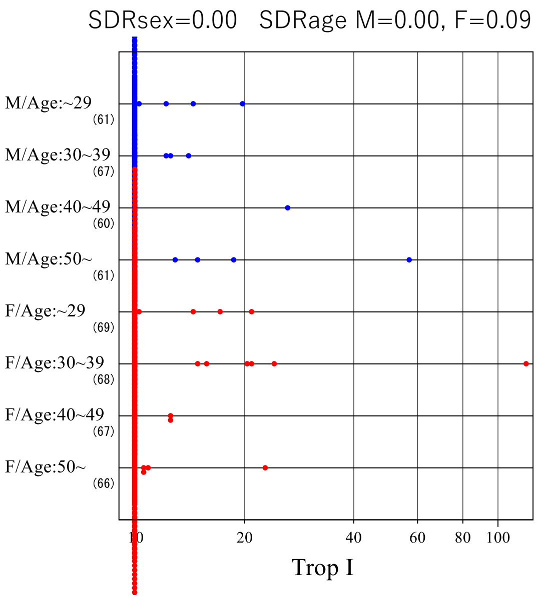

SDRsex=0.00 SDRage M=0.00, F=0.09
M/Age:~29
(61)
M/Age:30~39
(67)
M/Age:40~49
(60)
M/Age:50~
(61)
F/Age:~29
(69)
F/Age:30~39
(68)
F/Age:40~49
(67)
F/Age:50~
(66)
10
20
40
60
80
100
Trop I
