# Supplementary figures and images for: Determination of reference intervals for common chemistry and immunoassay tests for Kenyan adults based on an internationally harmonized protocol and up-to-date statistical methods
Source: PLoS One. 2020 Jul 9;15(7):e0235234. doi: 10.1371/journal.pone.0235234 (PMC7347104; doi:10.1371/journal.pone.0235234)

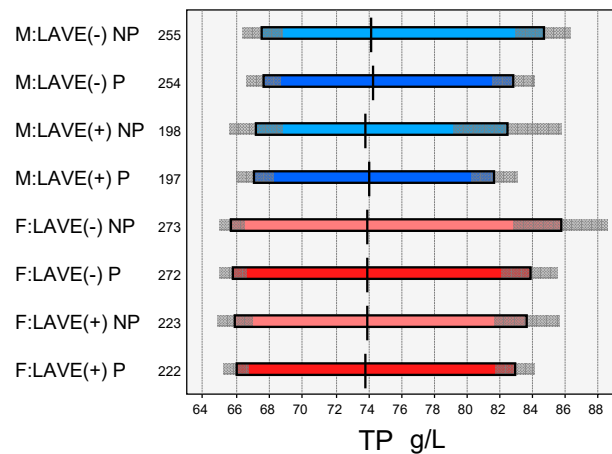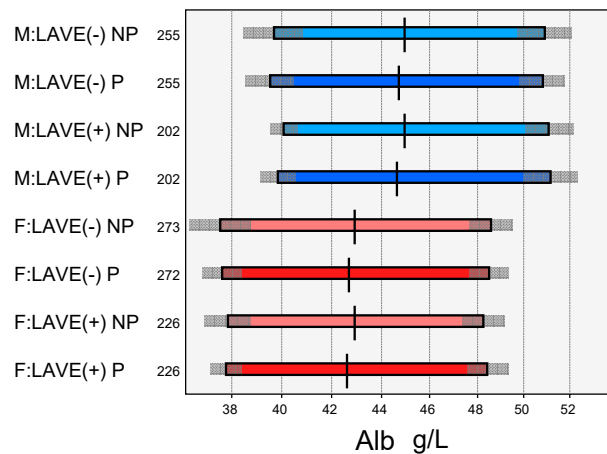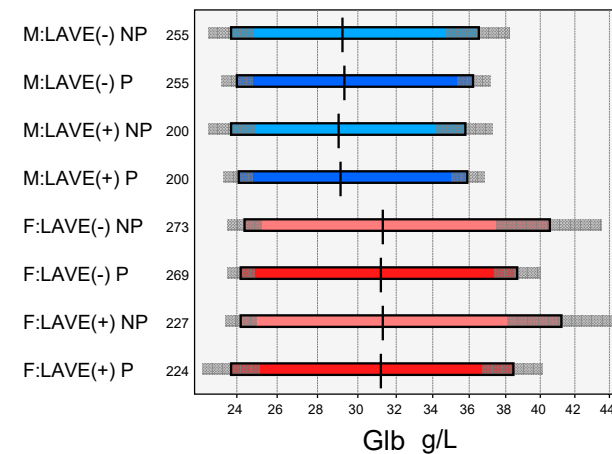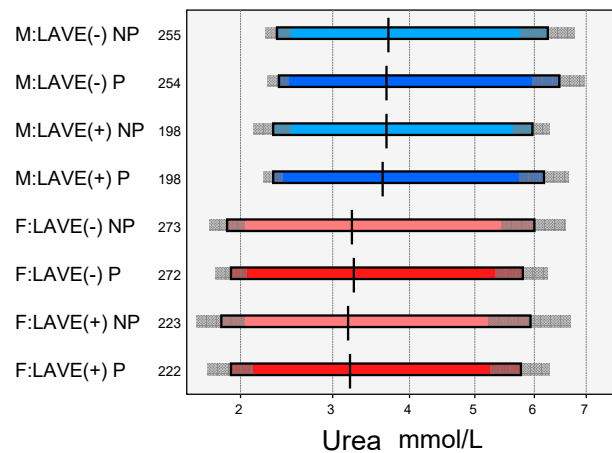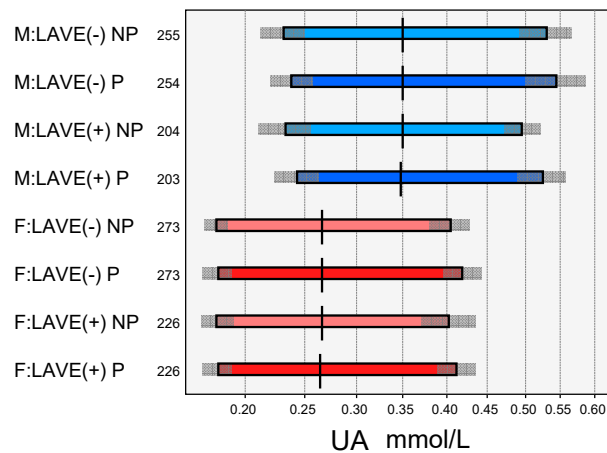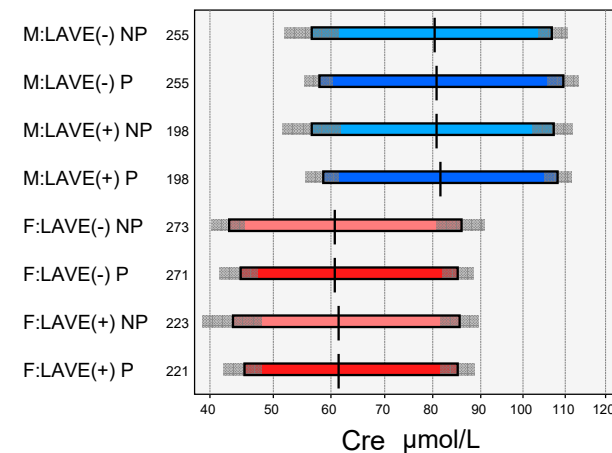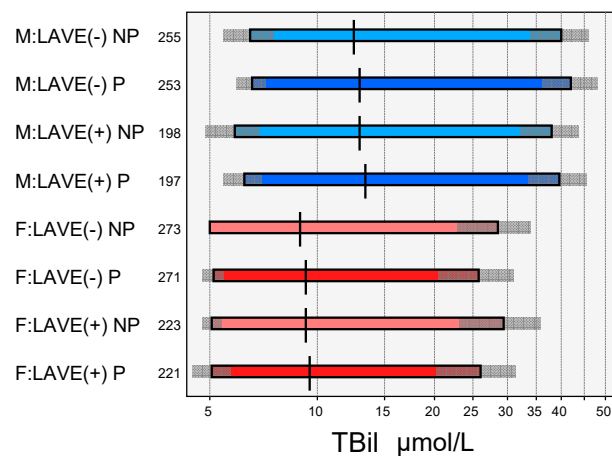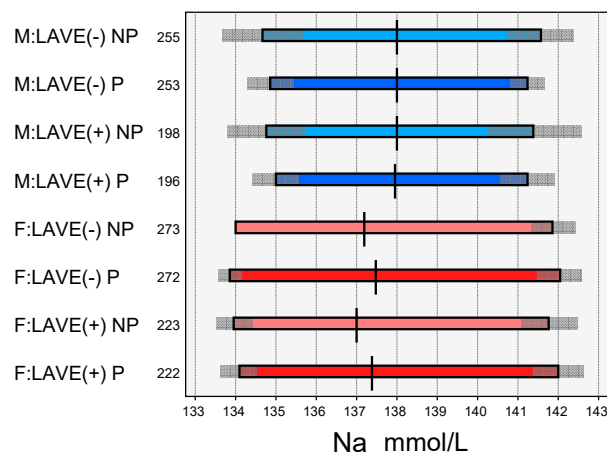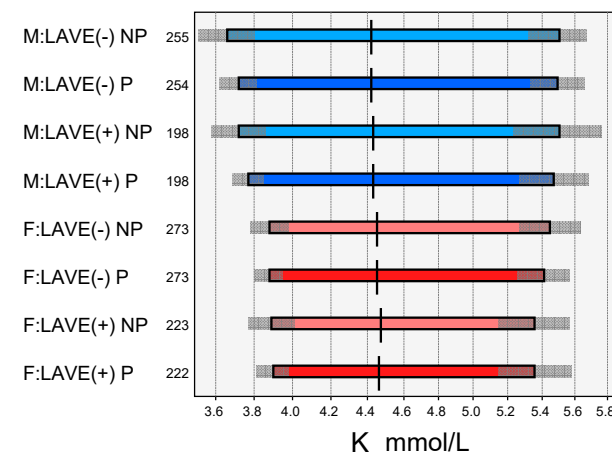

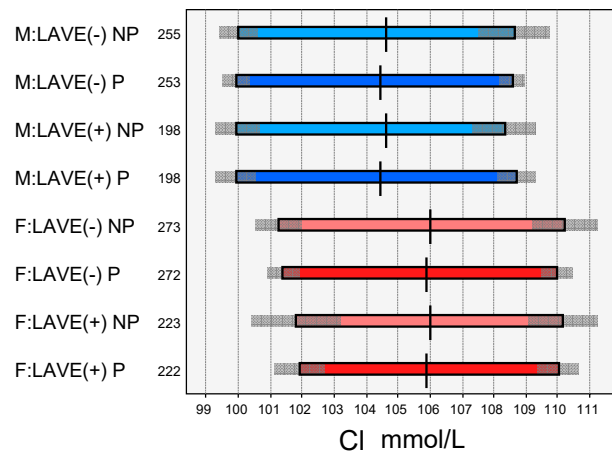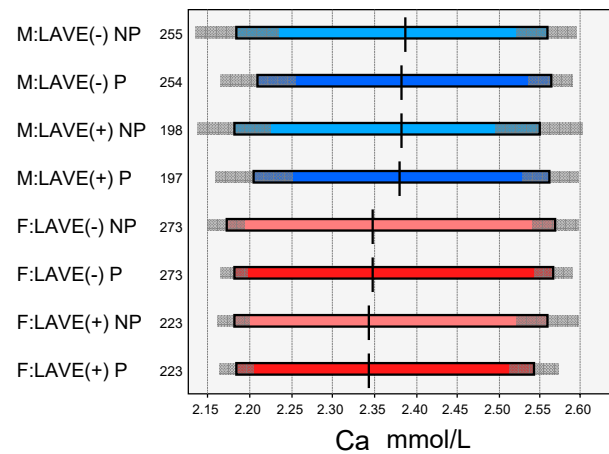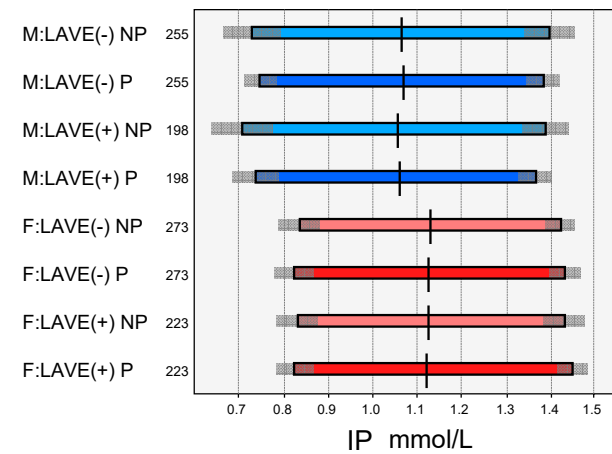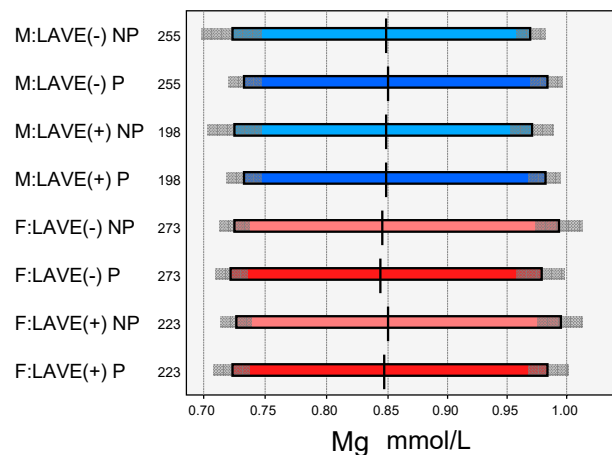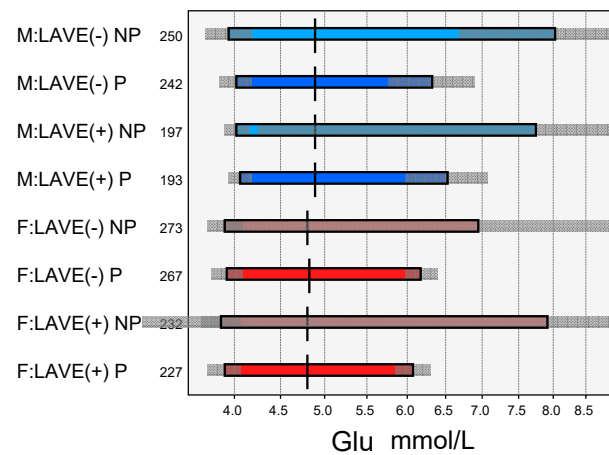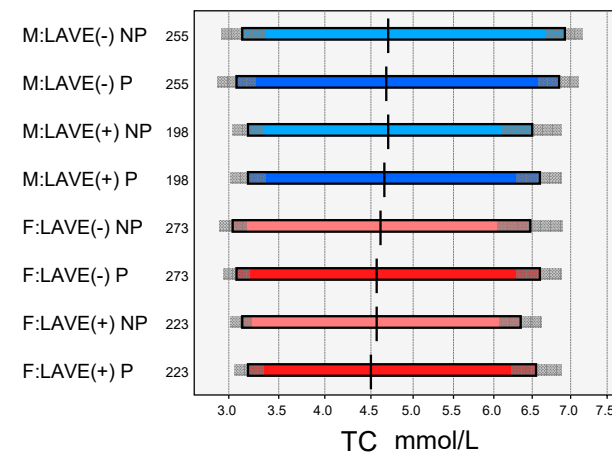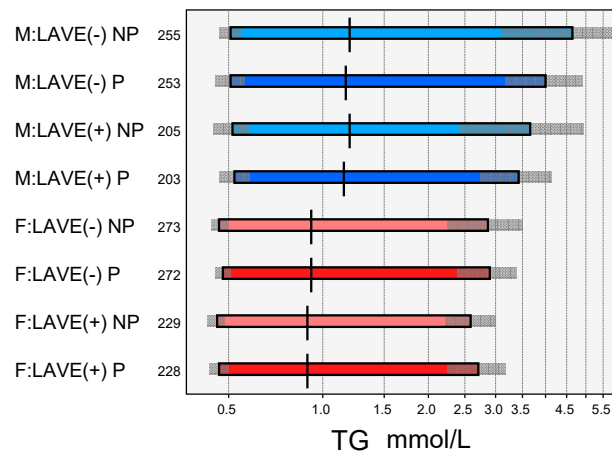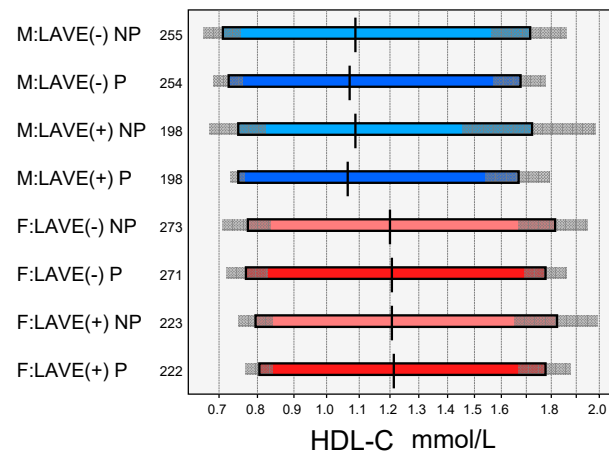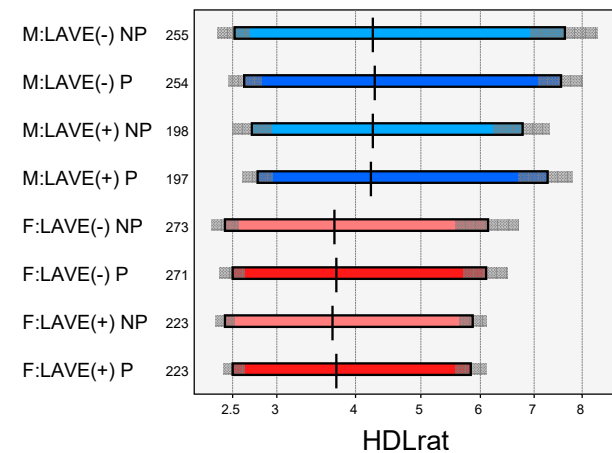

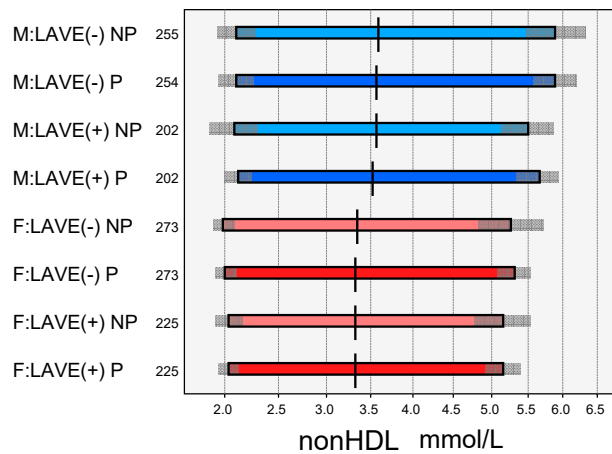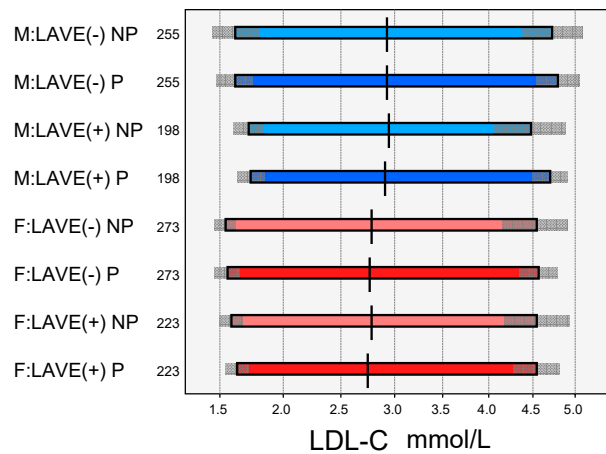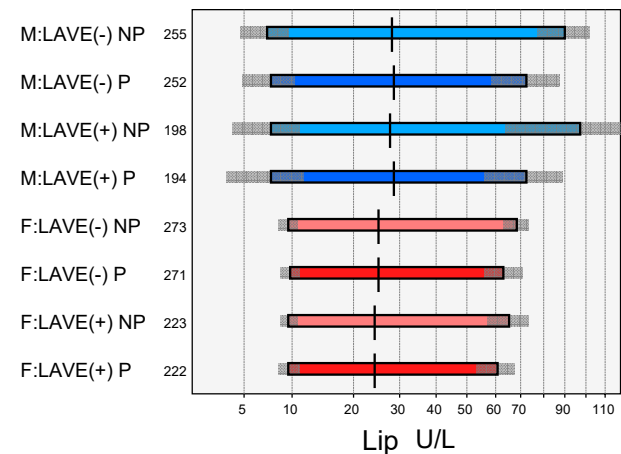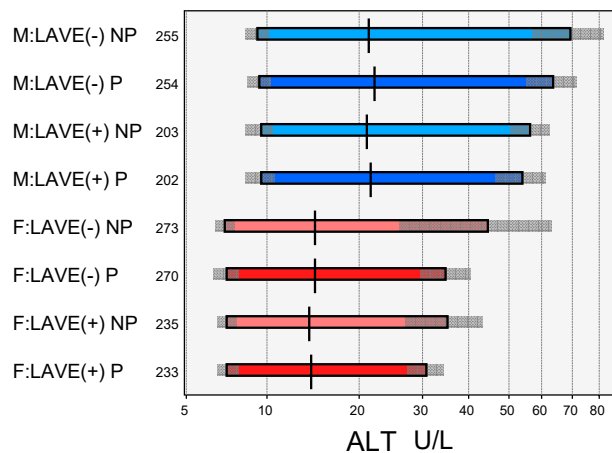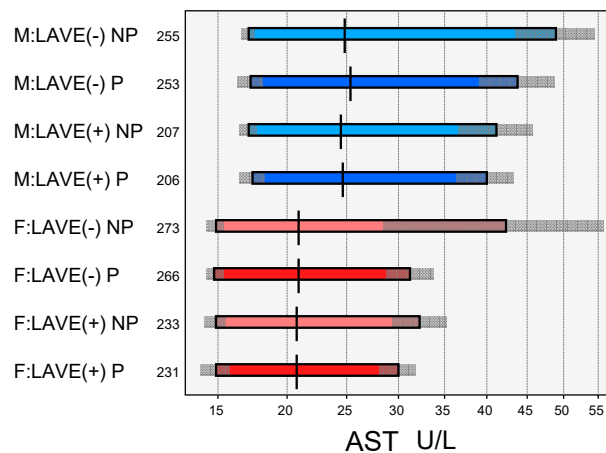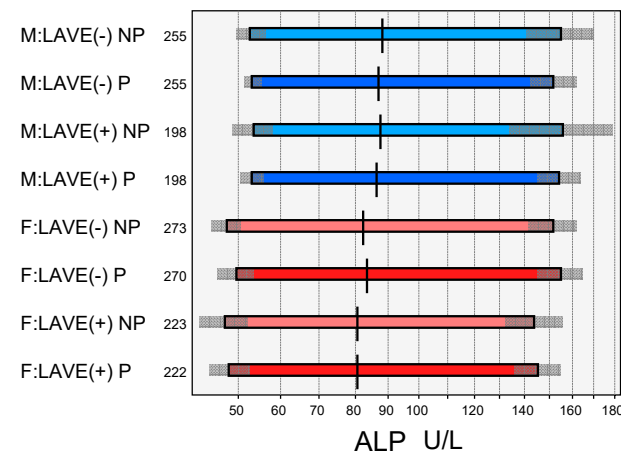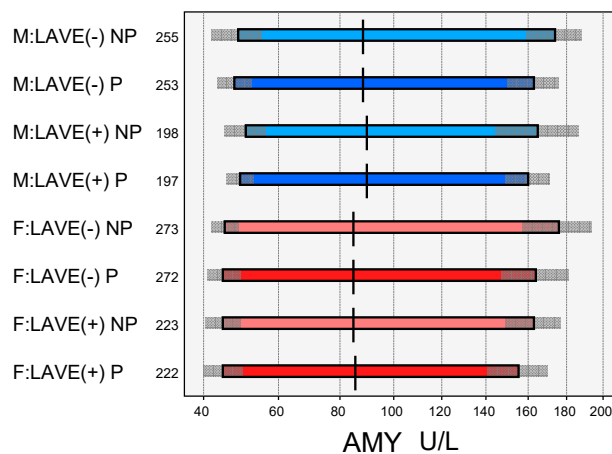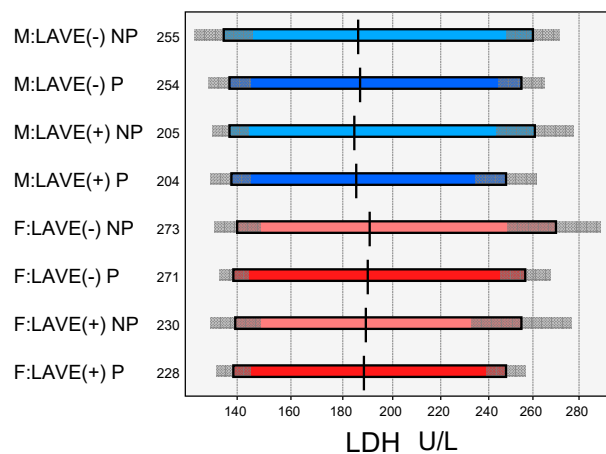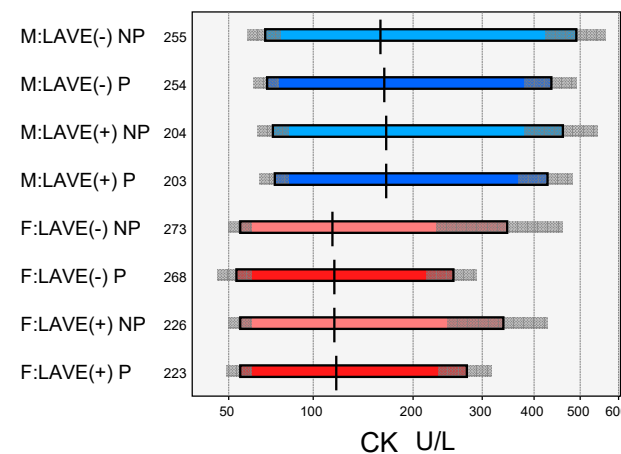

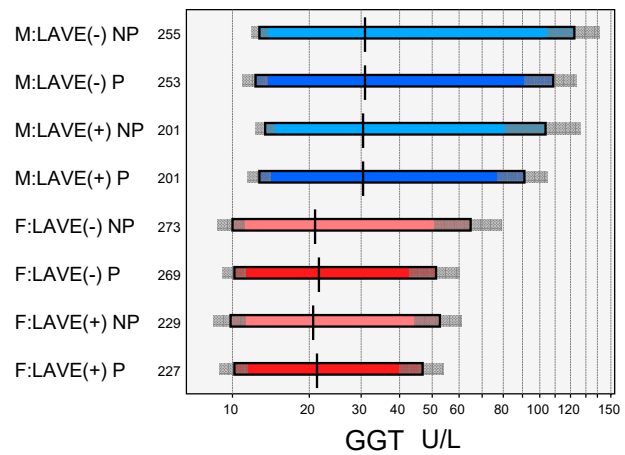

Supplement: S2 Fig — The RIs of all analytes were derived in four ways by parametric (P) or nonparametric (NP) method with/without the LAVE procedure, separately in males+Females (MF), males, and females. Each horizontal bar represents the range of the RI, and the vertical line in the center corresponds to the mid-point. The shades on both ends of the bar represent 90%CI for the limits of the RI predicted by the bootstrap method. (PDF) [file pone.0235234.s002.pdf]

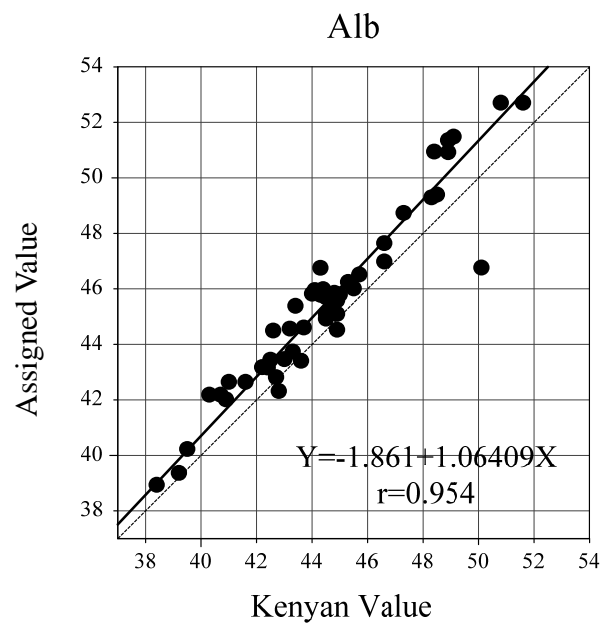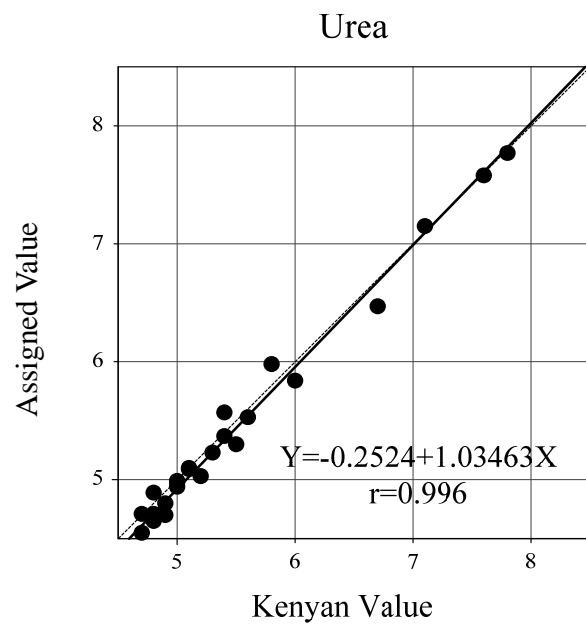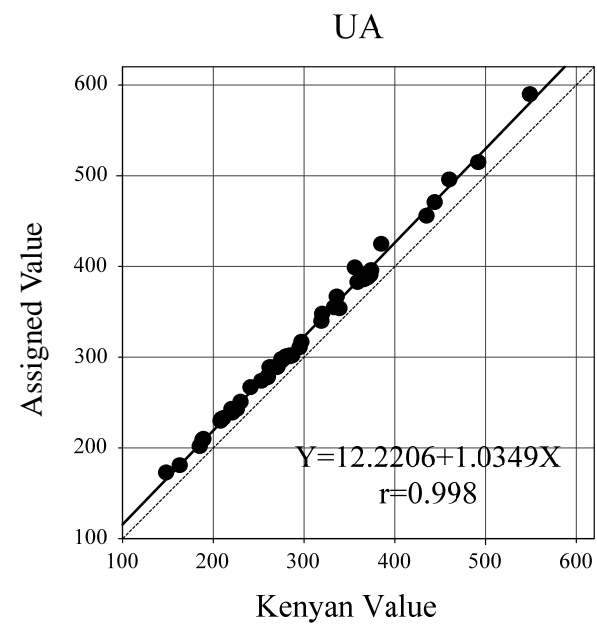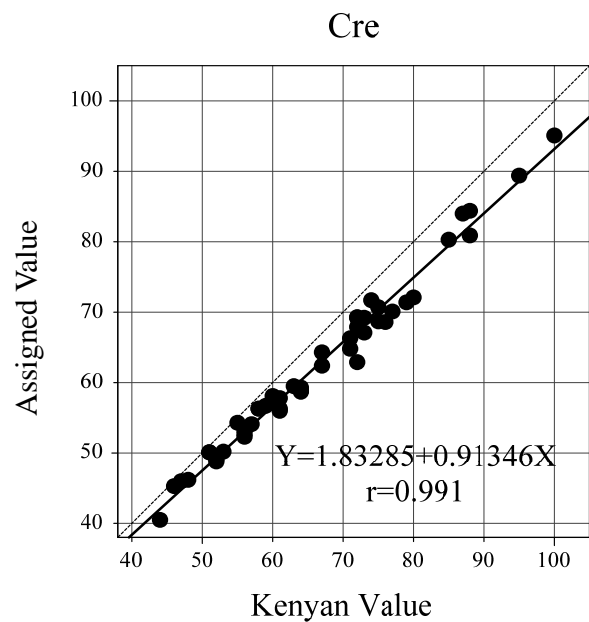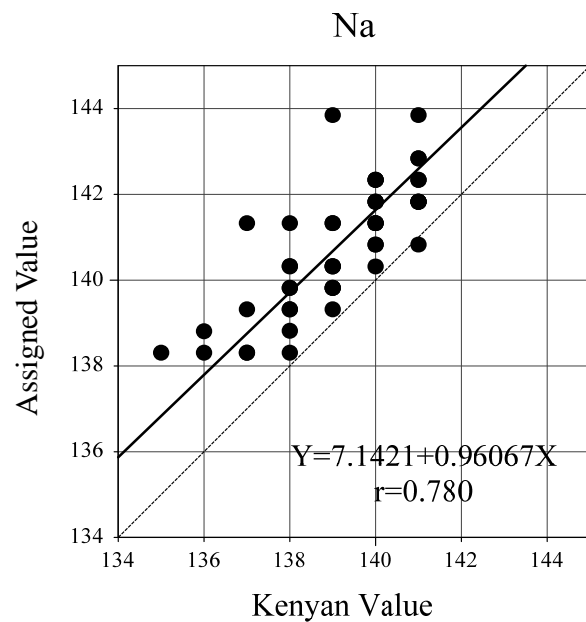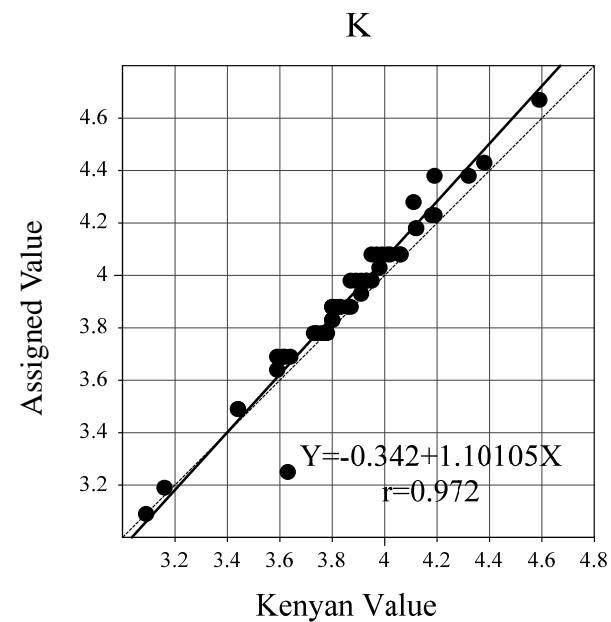

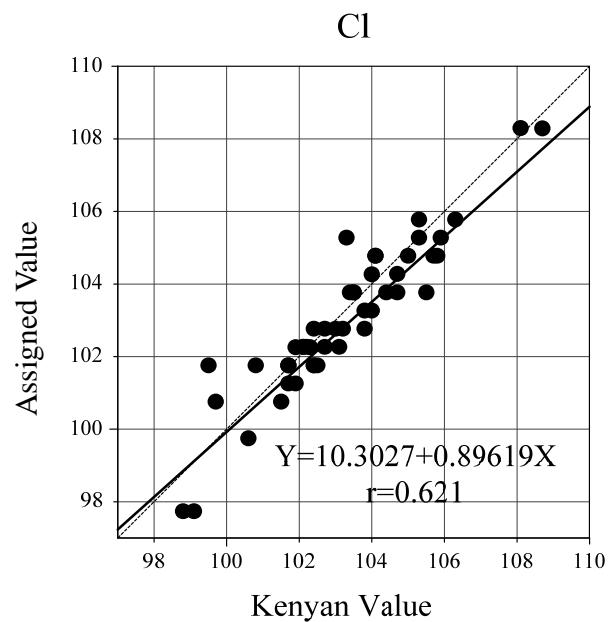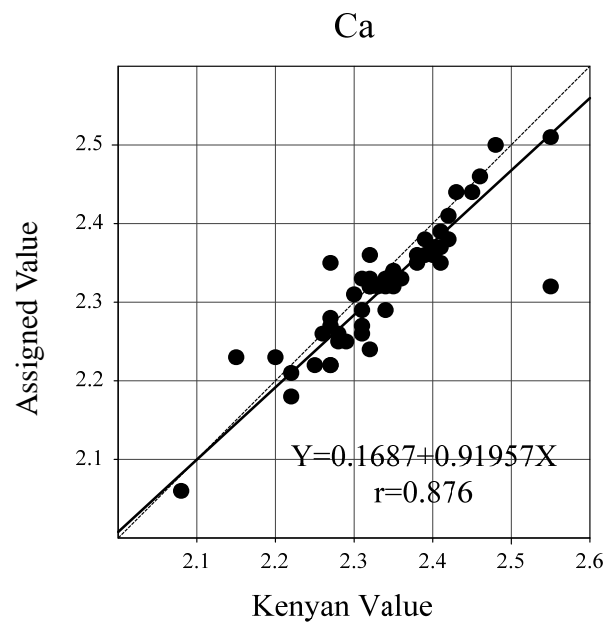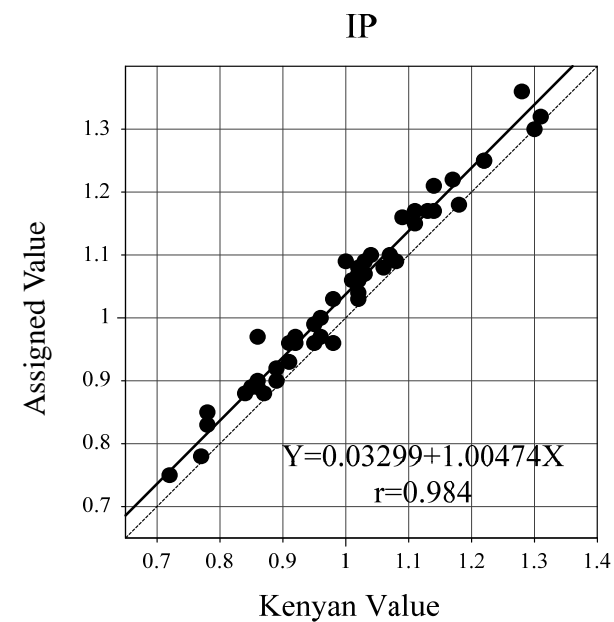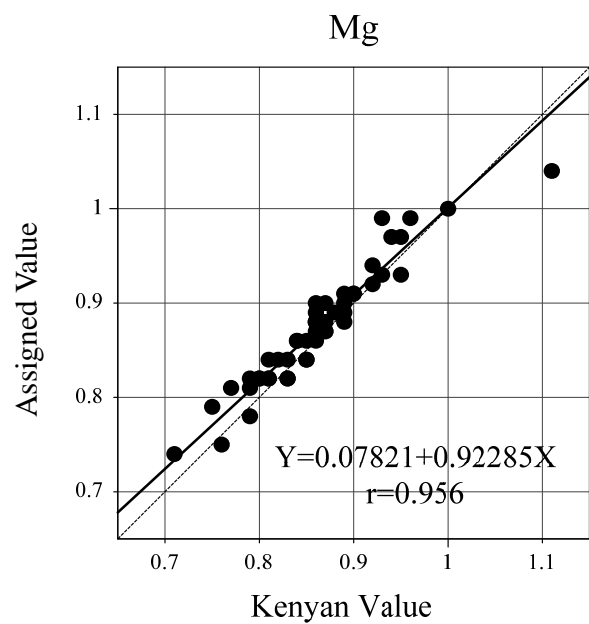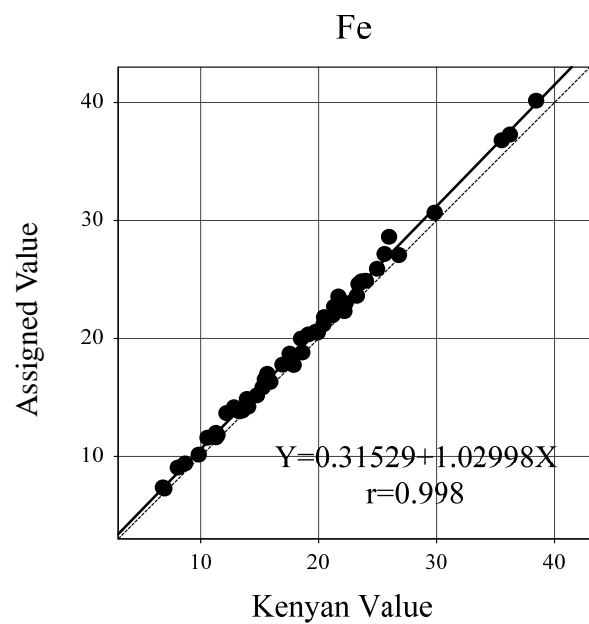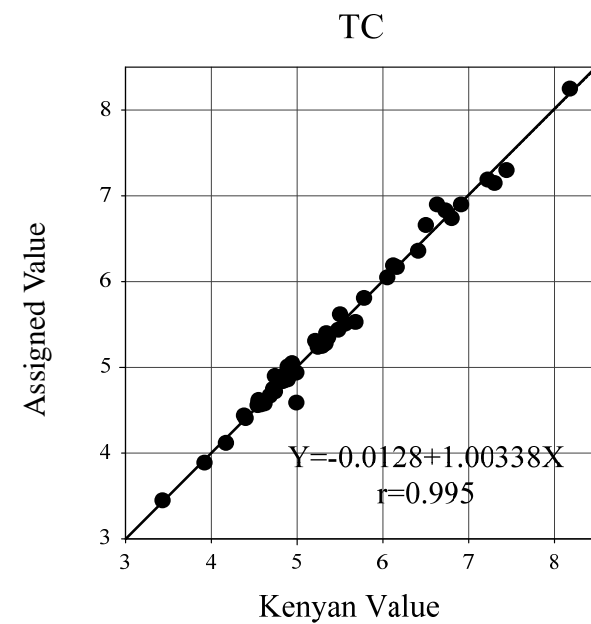

TG

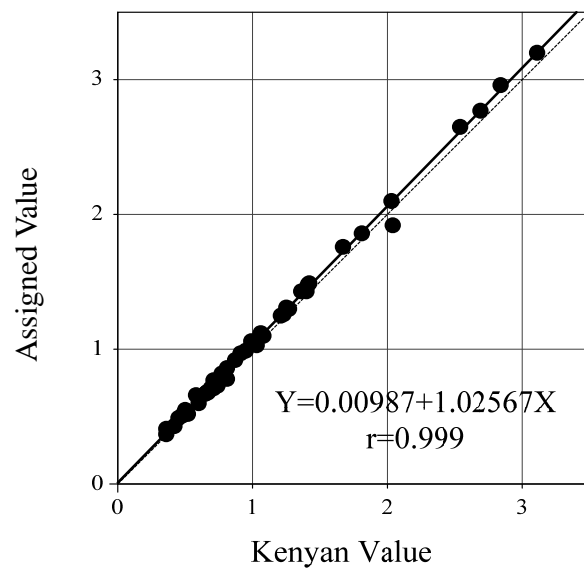

HDL-C

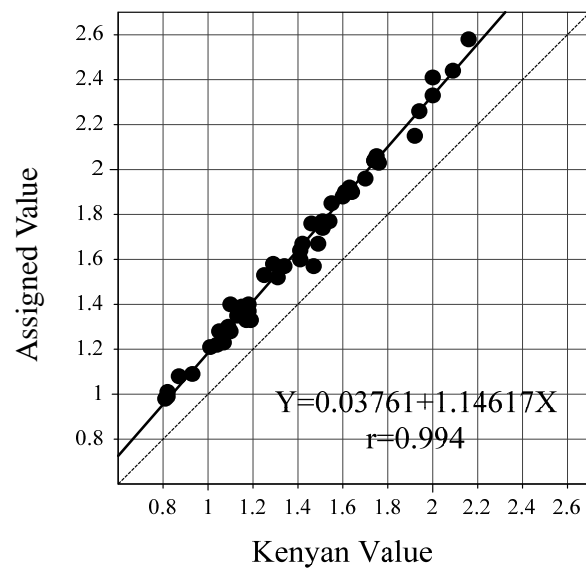

LDL-C

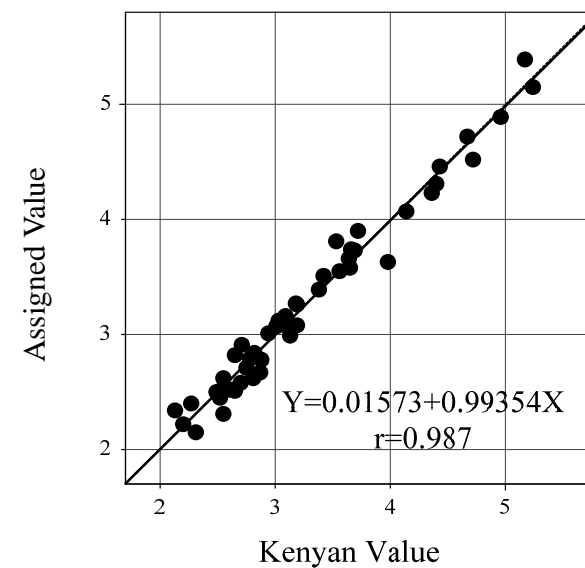

AST

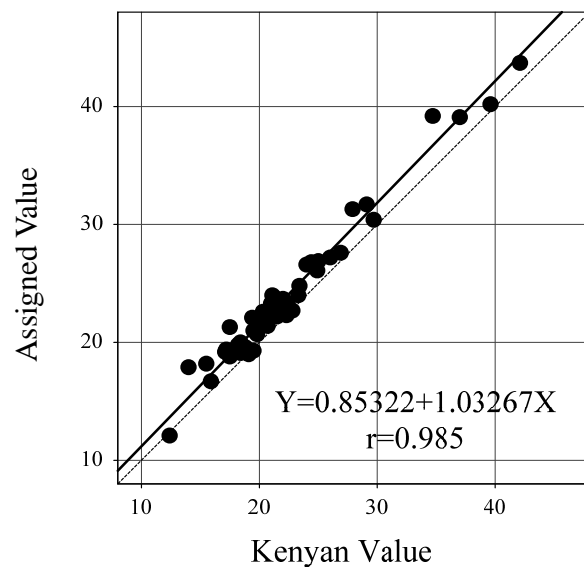

ALT

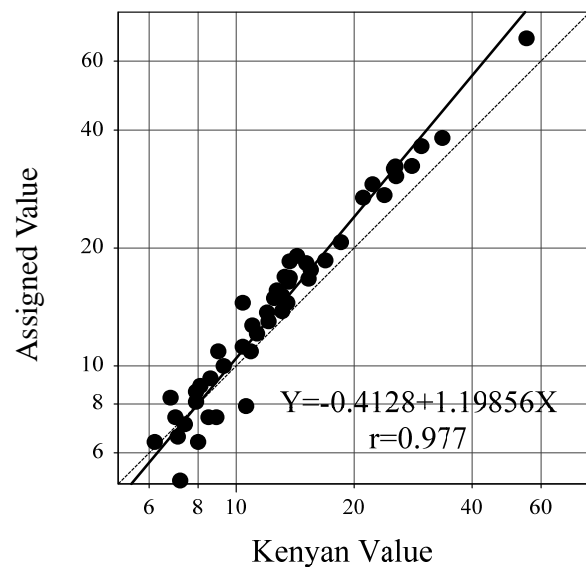

LDH

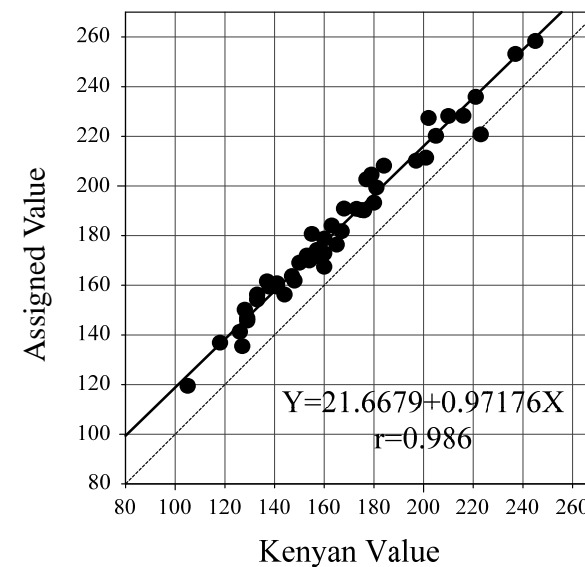

ALP

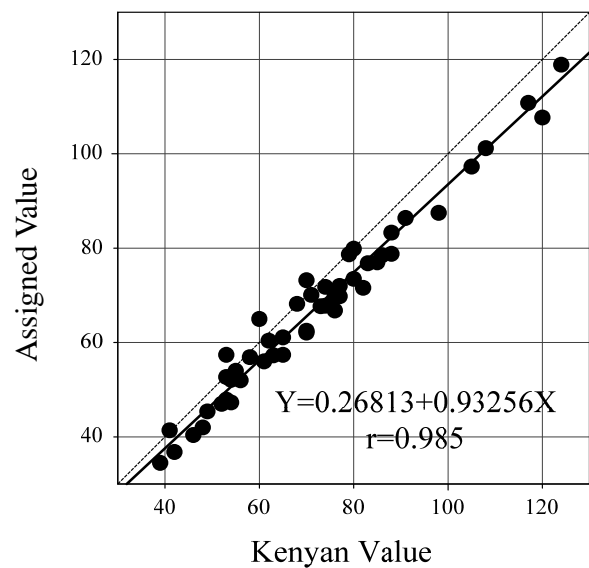

GGT

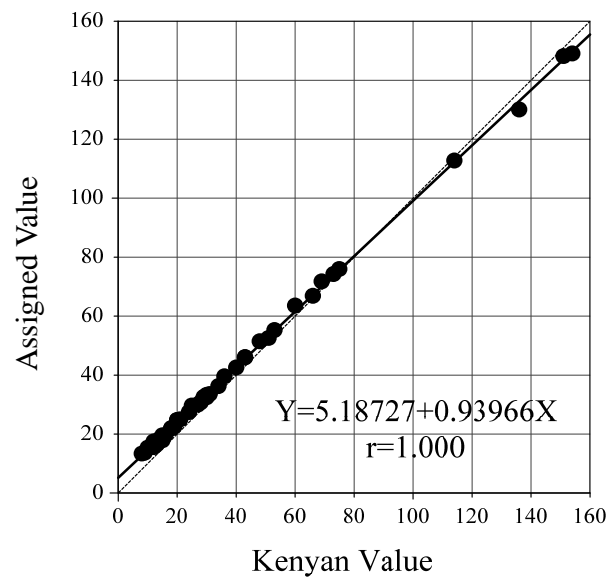

CK

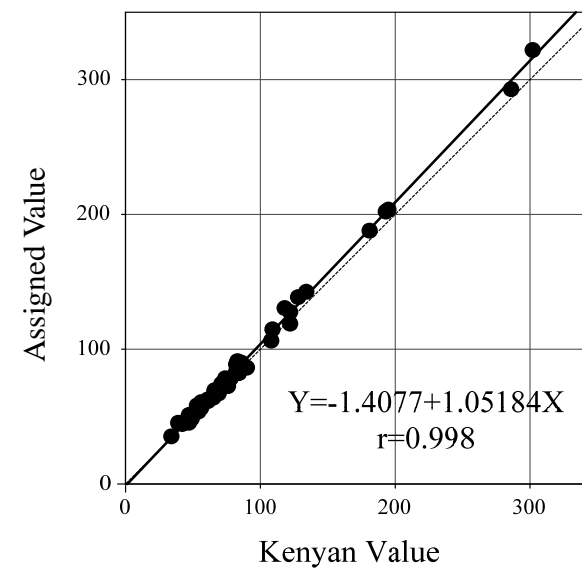

AMY

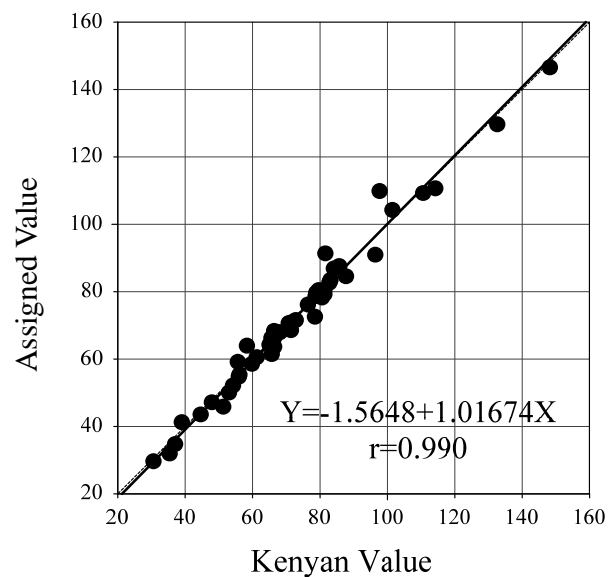

CRP

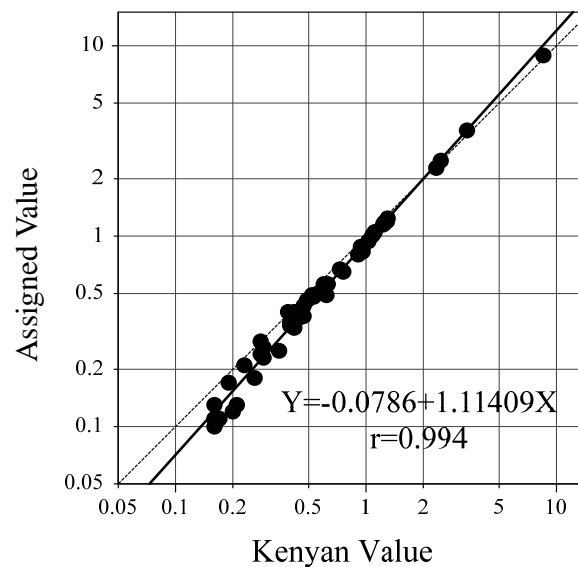

Tf

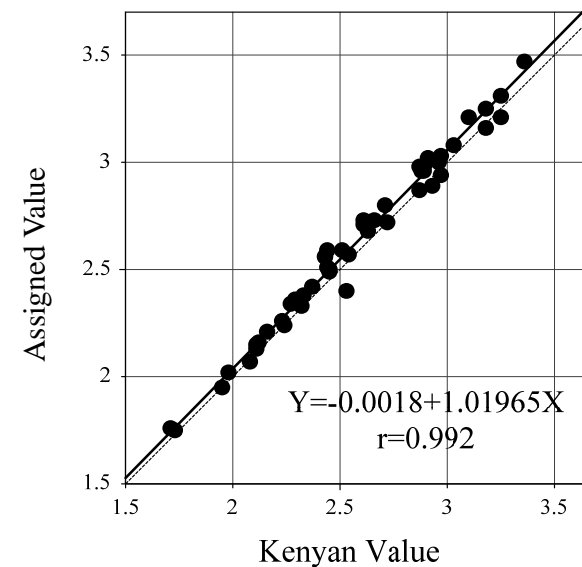

Supplement: S4 Fig — The panel of sera from 50 healthy volunteers, each of which were value assigned for 40 chemistry analytes were measured. Our measured values were plotted on Y-axis and assigned values on X-axis. Major axis linear regression was used as a structural relationship for the method comparison. The Y = X line is shown as a diagonal broken line. (PDF) [file pone.0235234.s004.pdf]
