# Supplementary material for: Determination of reference intervals for common chemistry and immunoassay tests for Kenyan adults based on an internationally harmonized protocol and up-to-date statistical methods
Source: PLoS One. 2020 Jul 9;15(7):e0235234. doi: 10.1371/journal.pone.0235234 (PMC7347104; doi:10.1371/journal.pone.0235234)

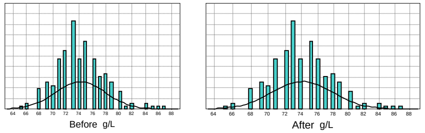

TP(g/L) M n=197
Para: 67.1 ~ 74.0 ~ 81.7
Nonpara: 67.2 ~ 73.8 ~ 82.4
Pow=0.888 TPos=62.31
Kurt=2.844 Skew=0.131

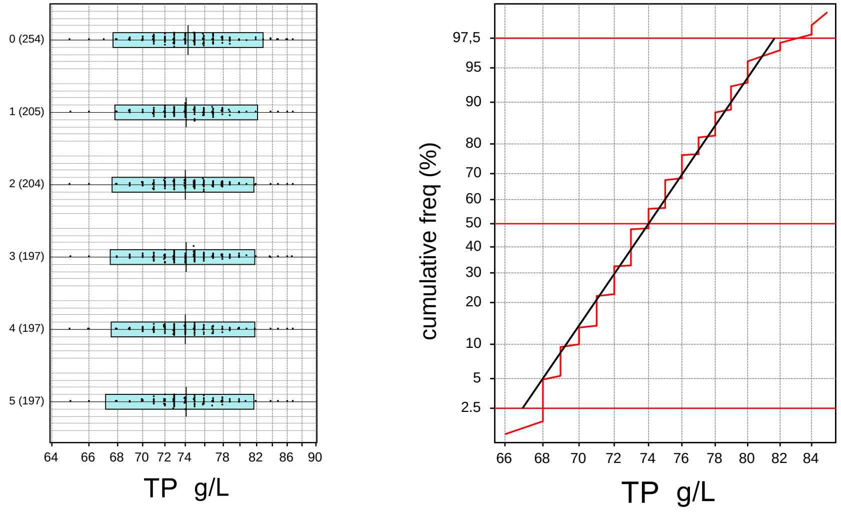

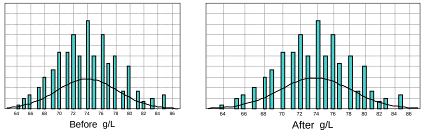

TP(g/L) F n=222
Para: 66.0 ~ 73.8 ~ 83.0
Nonpara: 65.9 ~ 73.9 ~ 83.7
Pow=0.779 TPos=60.383
Kurt=2.642 Skew=-0.032

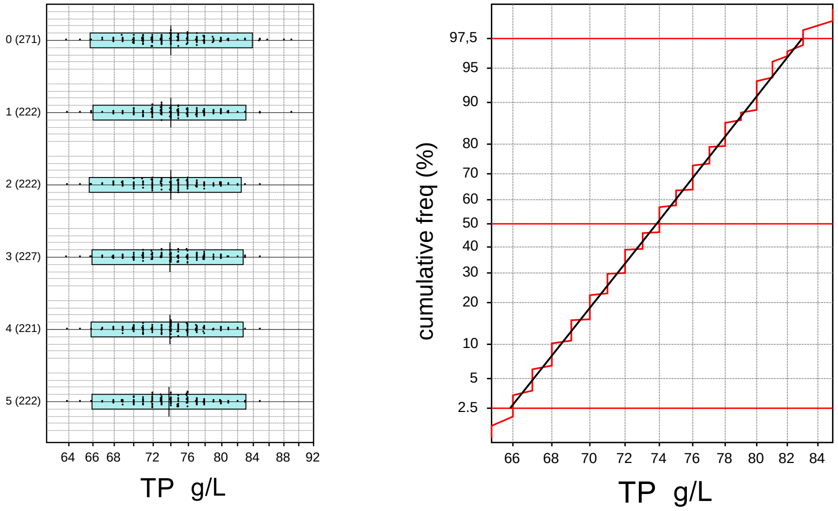

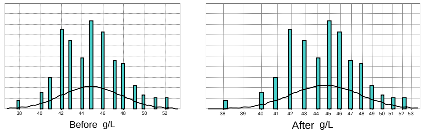

Alb(g/L) M n=202
Para: 39.84 ~ 44.64 ~ 51.17
Nonpara: 40.06 ~ 45.00 ~ 51.09
Pow=0.655 TPos=36.893
Kurt=2.751 Skew=-0.173

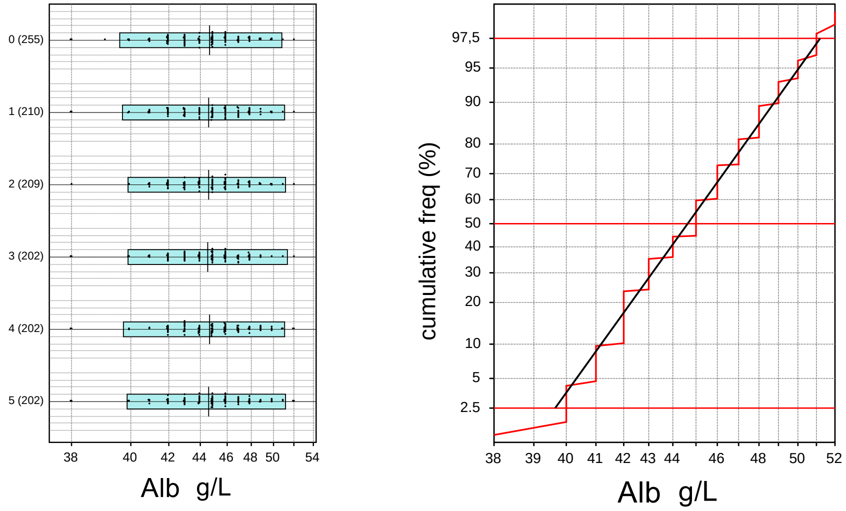

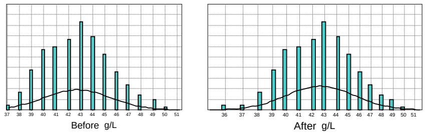

Alb(g/L) F n=226
Para: 37.80 ~ 42.65 ~ 48.46
Nonpara: 37.87 ~ 42.90 ~ 48.28
Pow=0.779 TPos=34.566
Kurt=2.579 Skew=-0.094

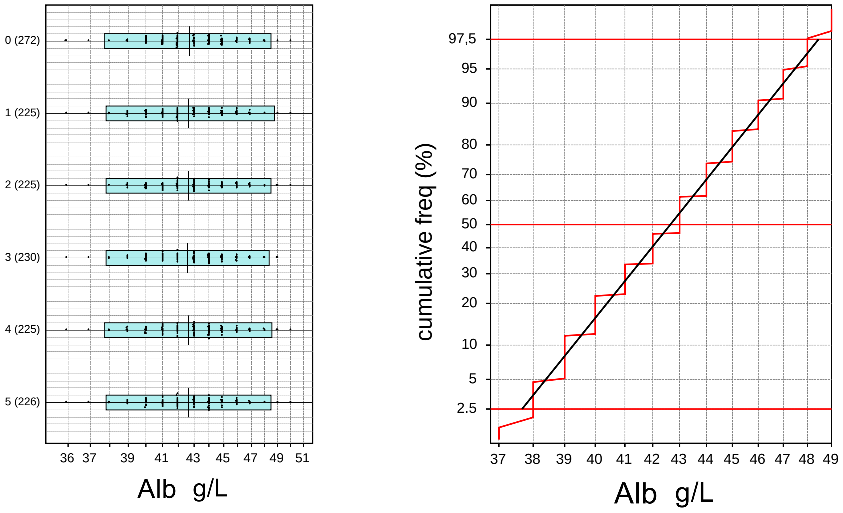

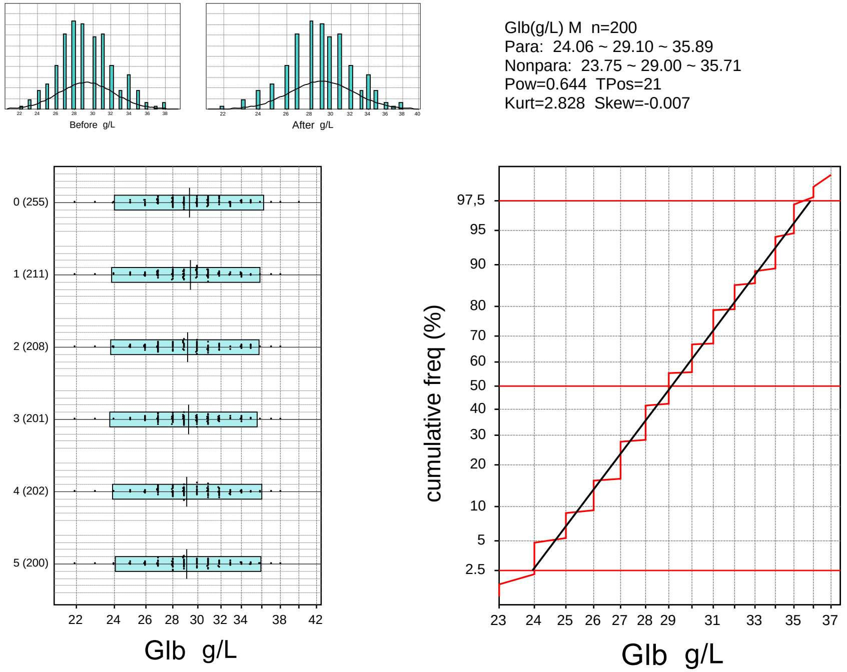
Glb(g/L) M n=200
Para: 24.06 ~ 29.10 ~ 35.89
Nonpara: 23.75 ~ 29.00 ~ 35.71
Pow=0.644 TPos=21
Kurt=2.828 Skew=-0.007
Before g/L
After g/L
0 (255)
1 (211)
2 (208)
3 (201)
4 (202)
5 (200)
Glb g/L
cumulative freq (%)
Glb g/L

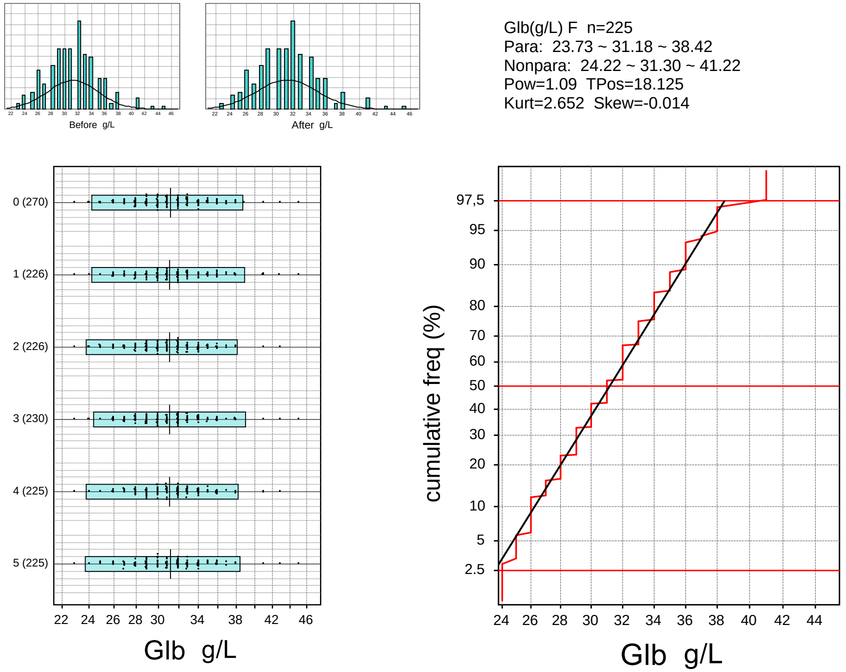
Glb(g/L) F n=225
Para: 23.73 ~ 31.18 ~ 38.42
Nonpara: 24.22 ~ 31.30 ~ 41.22
Pow=1.09 TPos=18.125
Kurt=2.652 Skew=-0.014
Before g/L
After g/L
0 (270)
1 (226)
2 (226)
3 (230)
4 (225)
5 (225)
Glb g/L
cumulative freq (%)
Glb g/L

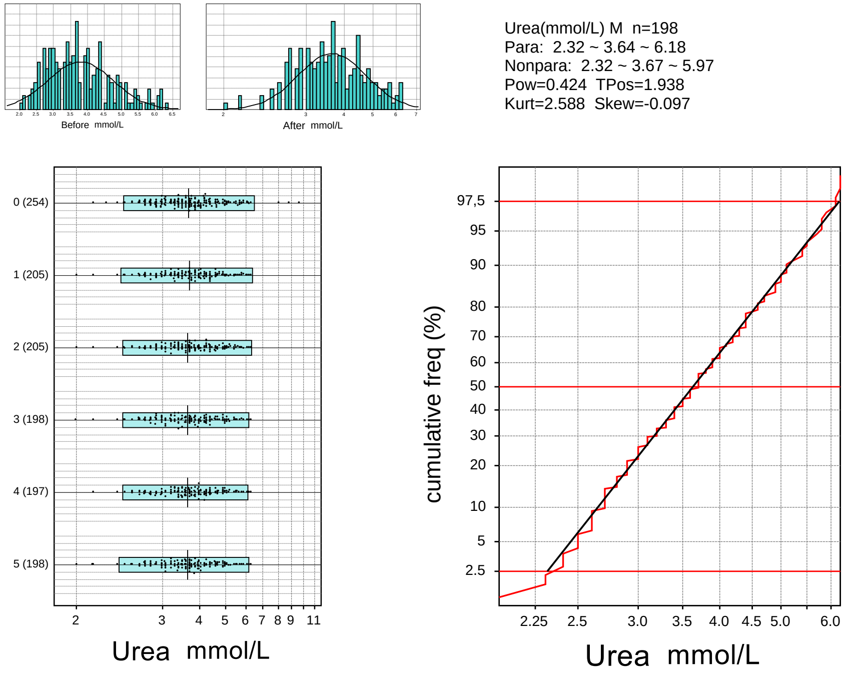
Urea(mmol/L) M n=198
Para: 2.32 ~ 3.64 ~ 6.18
Nonpara: 2.32 ~ 3.67 ~ 5.97
Pow=0.424 TPos=1.938
Kurt=2.588 Skew=-0.097
Before mmol/L
After mmol/L
0 (254)
1 (205)
2 (205)
3 (198)
4 (197)
5 (198)
Urea mmol/L
cumulative freq (%)
Urea mmol/L

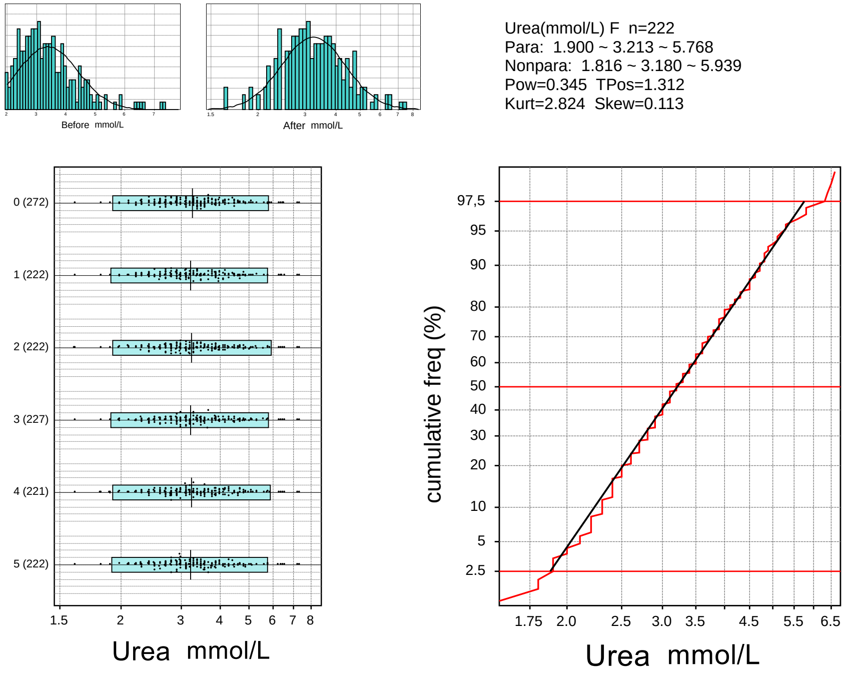
Urea(mmol/L) F n=222
Para: 1.900 ~ 3.213 ~ 5.768
Nonpara: 1.816 ~ 3.180 ~ 5.939
Pow=0.345 TPos=1.312
Kurt=2.824 Skew=0.113
Before mmol/L
After mmol/L
0 (272)
1 (222)
2 (222)
3 (227)
4 (221)
5 (222)
Urea mmol/L
cumulative freq (%)
Urea mmol/L

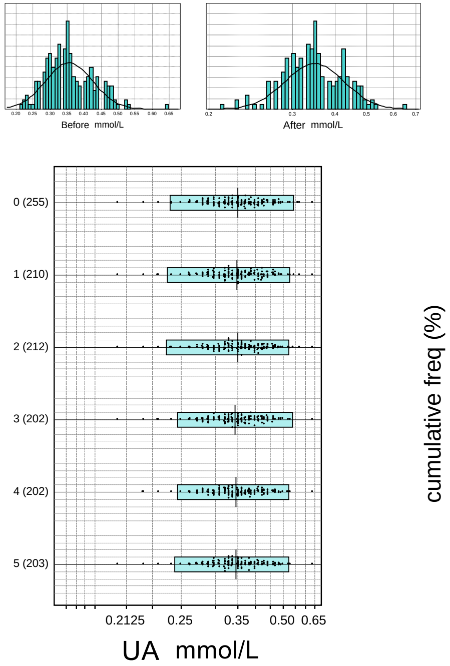

UA(mmol/L) M n=203
Para: 0.243 ~ 0.346 ~ 0.524
Nonpara: 0.233 ~ 0.348 ~ 0.496
Pow=0.406 TPos=0.186
Kurt=2.735 Skew=-0.068

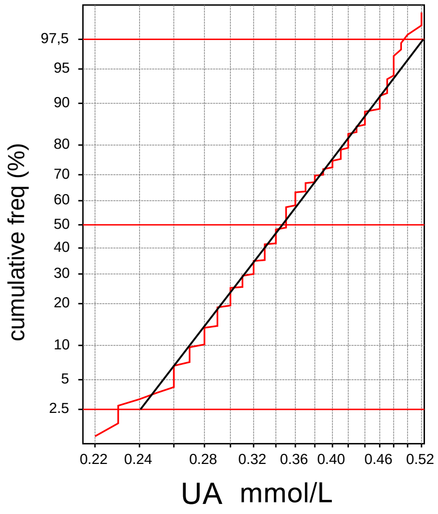

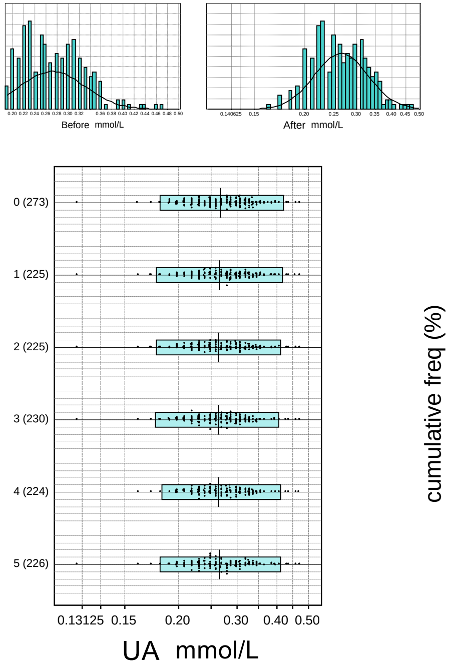

UA(mmol/L) F n=226
Para: 0.179 ~ 0.265 ~ 0.412
Nonpara: 0.178 ~ 0.267 ~ 0.402
Pow=0.436 TPos=0.138
Kurt=2.655 Skew=-0.102

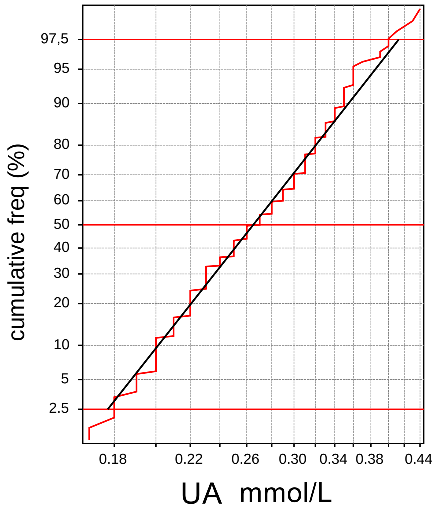

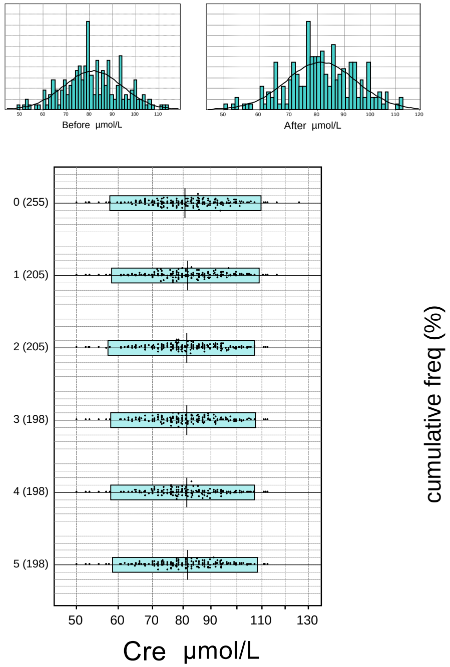

Cre(μmol/L) M n=198
Para: 58.4 ~ 81.5 ~ 108.1
Nonpara: 56.7 ~ 80.6 ~ 107.0
Pow=0.795 TPos=42.011
Kurt=2.586 Skew=0.056

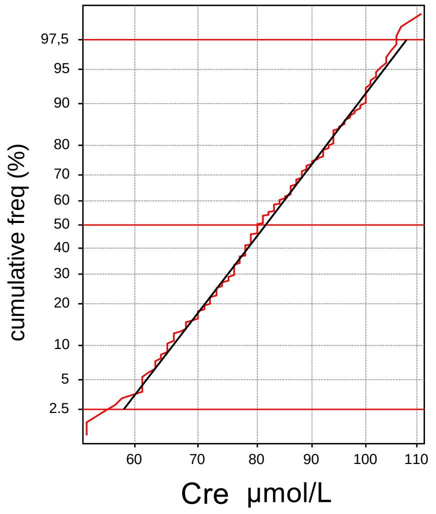

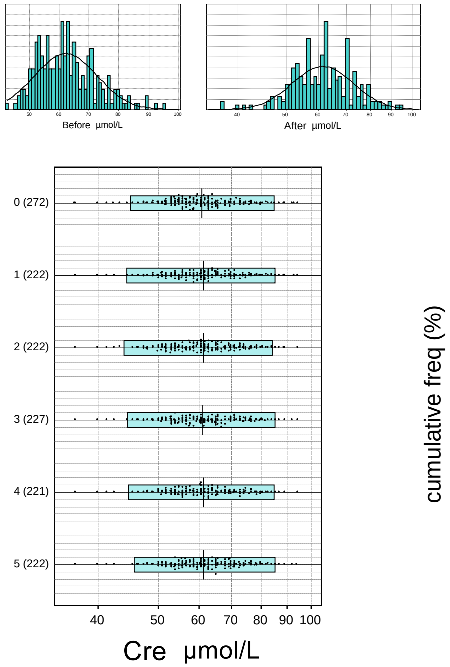

Cre(μmol/L) F n=222
Para: 45.15 ~ 61.45 ~ 85.21
Nonpara: 43.47 ~ 61.40 ~ 85.59
Pow=0.59 TPos=37.159
Kurt=2.78 Skew=0.06

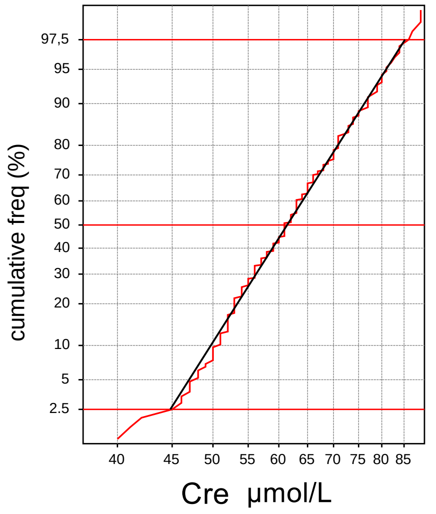

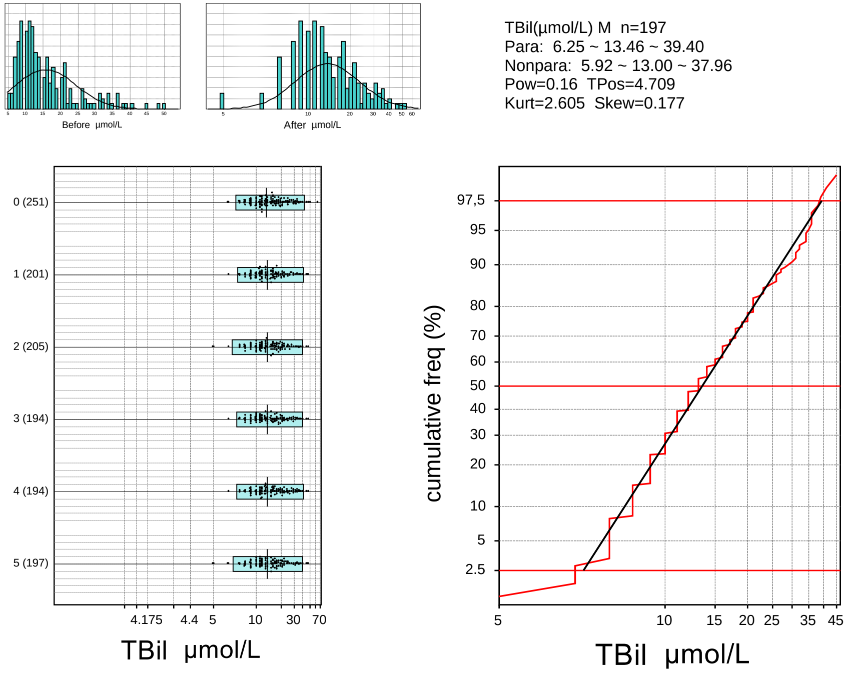
Before µmol/L
After µmol/L
TBil(µmol/L) M n=197
Para: 6.25 ~ 13.46 ~ 39.40
Nonpara: 5.92 ~ 13.00 ~ 37.96
Pow=0.16 TPos=4.709
Kurt=2.605 Skew=0.177
0 (251)
1 (201)
2 (205)
3 (194)
4 (194)
5 (197)
TBil µmol/L
cumulative freq (%)
TBil µmol/L

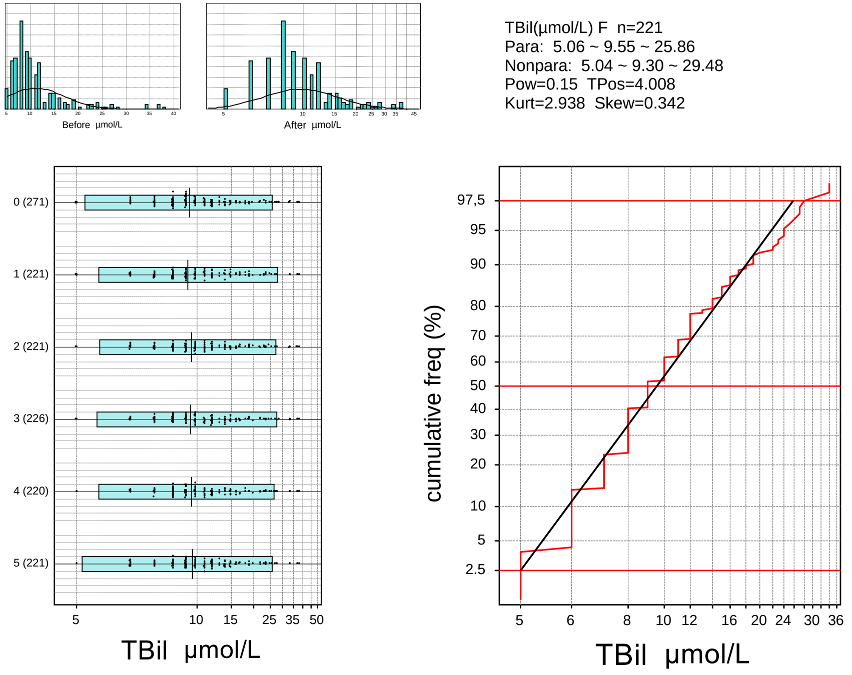
Before µmol/L
After µmol/L
TBil(µmol/L) F n=221
Para: 5.06 ~ 9.55 ~ 25.86
Nonpara: 5.04 ~ 9.30 ~ 29.48
Pow=0.15 TPos=4.008
Kurt=2.938 Skew=0.342
0 (271)
1 (221)
2 (221)
3 (226)
4 (220)
5 (221)
TBil µmol/L
cumulative freq (%)
TBil µmol/L

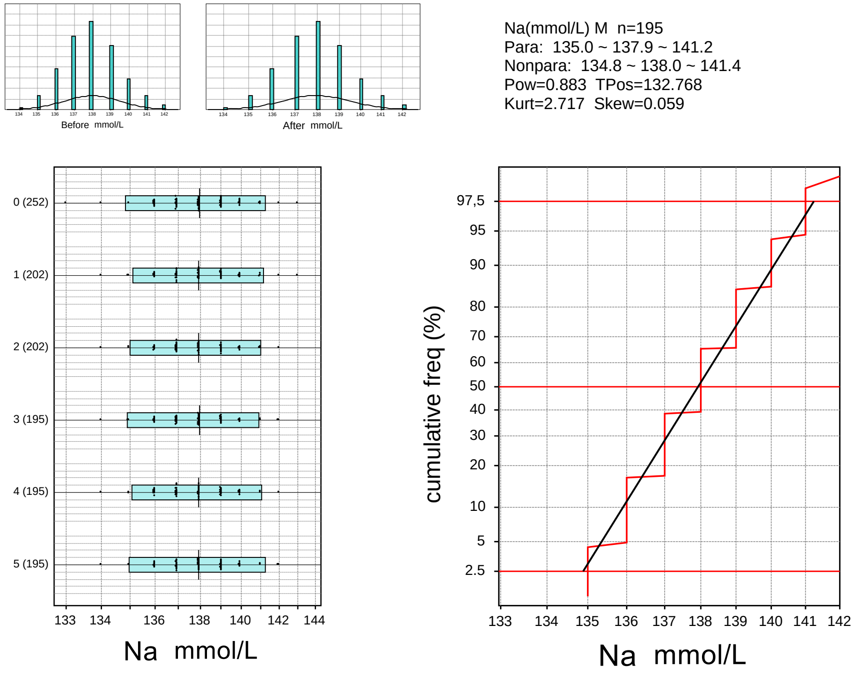
Before mmol/L
After mmol/L
Na(mmol/L) M n=195
Para: 135.0 ~ 137.9 ~ 141.2
Nonpara: 134.8 ~ 138.0 ~ 141.4
Pow=0.883 TPos=132.768
Kurt=2.717 Skew=0.059
0 (252)
1 (202)
2 (202)
3 (195)
4 (195)
5 (195)
Na mmol/L
cumulative freq (%)
Na mmol/L

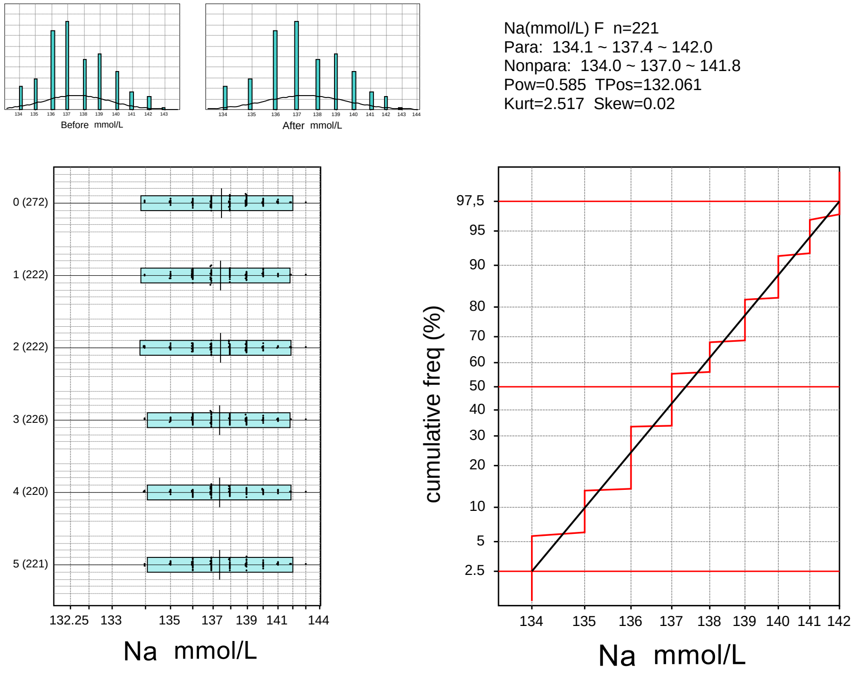
Before mmol/L
After mmol/L
Na(mmol/L) F n=221
Para: 134.1 ~ 137.4 ~ 142.0
Nonpara: 134.0 ~ 137.0 ~ 141.8
Pow=0.585 TPos=132.061
Kurt=2.517 Skew=0.02
0 (272)
1 (222)
2 (222)
3 (226)
4 (220)
5 (221)
Na mmol/L
cumulative freq (%)
Na mmol/L

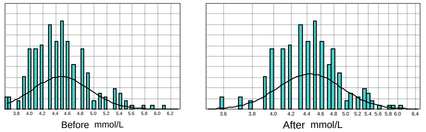

K(mmol/L) M n=198
Para: 3.76 ~ 4.43 ~ 5.47
Nonpara: 3.72 ~ 4.43 ~ 5.50
Pow=0.5 TPos=3.419
Kurt=2.907 Skew=0.011

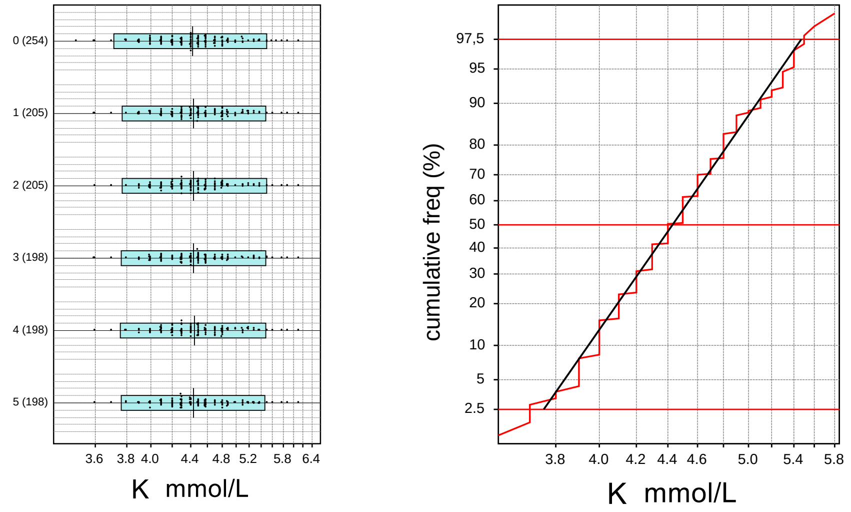

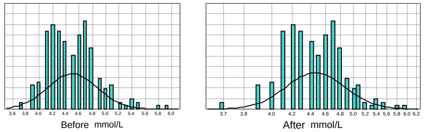

K(mmol/L) F n=222
Para: 3.89 ~ 4.46 ~ 5.36
Nonpara: 3.89 ~ 4.47 ~ 5.35
Pow=0.501 TPos=3.609
Kurt=2.788 Skew=-0.095

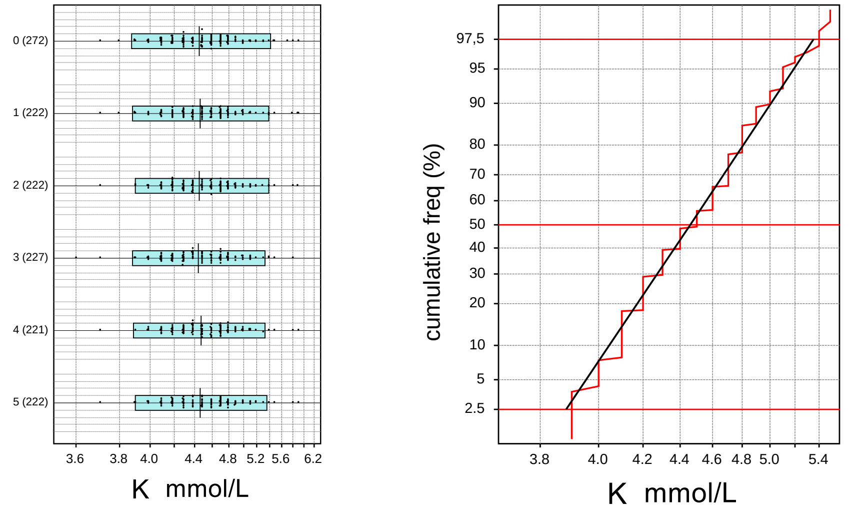

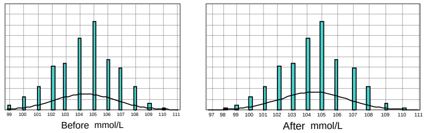

Cl(mmol/L) M n=198
Para: 99.9 ~ 104.4 ~ 108.7
Nonpara: 100.0 ~ 104.6 ~ 108.3
Pow=1.185 TPos=95.207
Kurt=2.479 Skew=-0.143

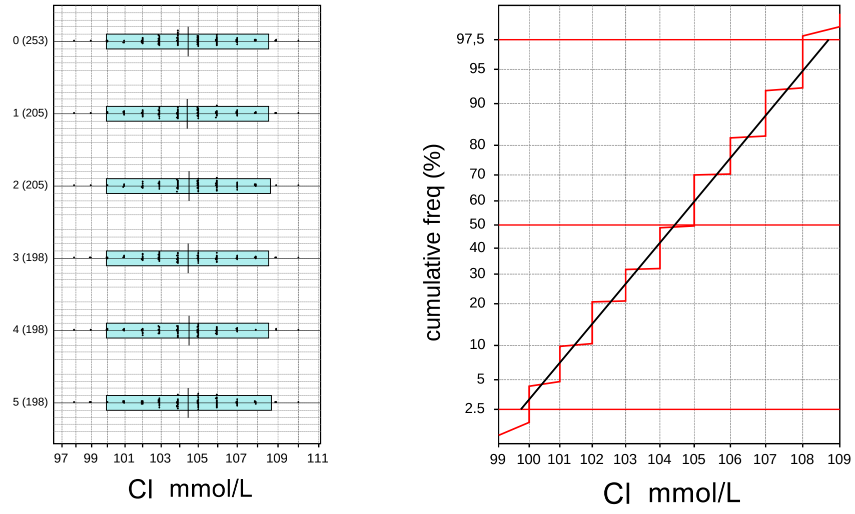

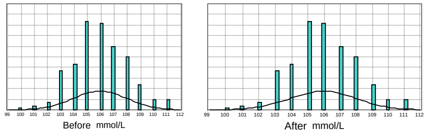

Cl(mmol/L) F n=221
Para: 101.9 ~ 105.9 ~ 110.0
Nonpara: 101.8 ~ 106.0 ~ 110.2
Pow=0.964 TPos=98.64
Kurt=2.523 Skew=0.019

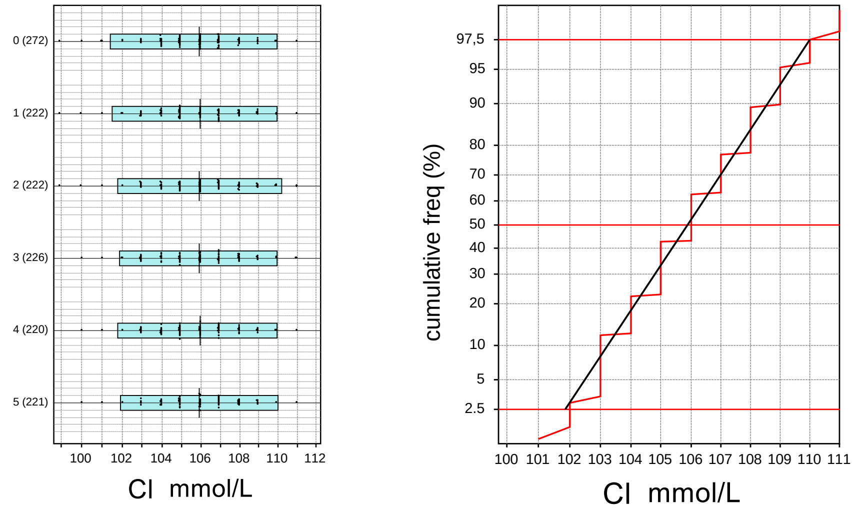

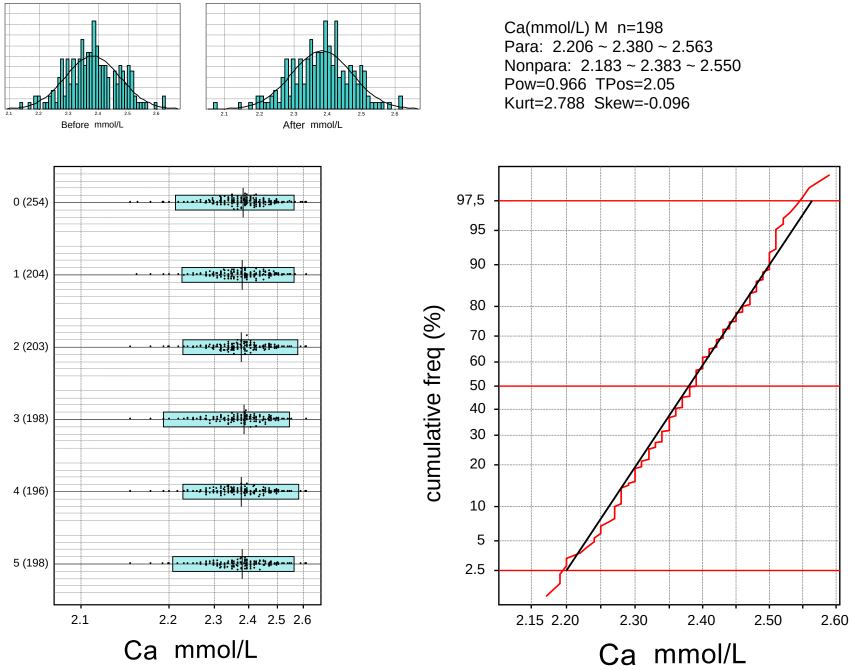
Before mmol/L
After mmol/L
Ca(mmol/L) M n=198
Para: 2.206 ~ 2.380 ~ 2.563
Nonpara: 2.183 ~ 2.383 ~ 2.550
Pow=0.966 TPos=2.05
Kurt=2.788 Skew=-0.096
0 (254)
1 (204)
2 (203)
3 (198)
4 (196)
5 (198)
Ca mmol/L
cumulative freq (%)
Ca mmol/L

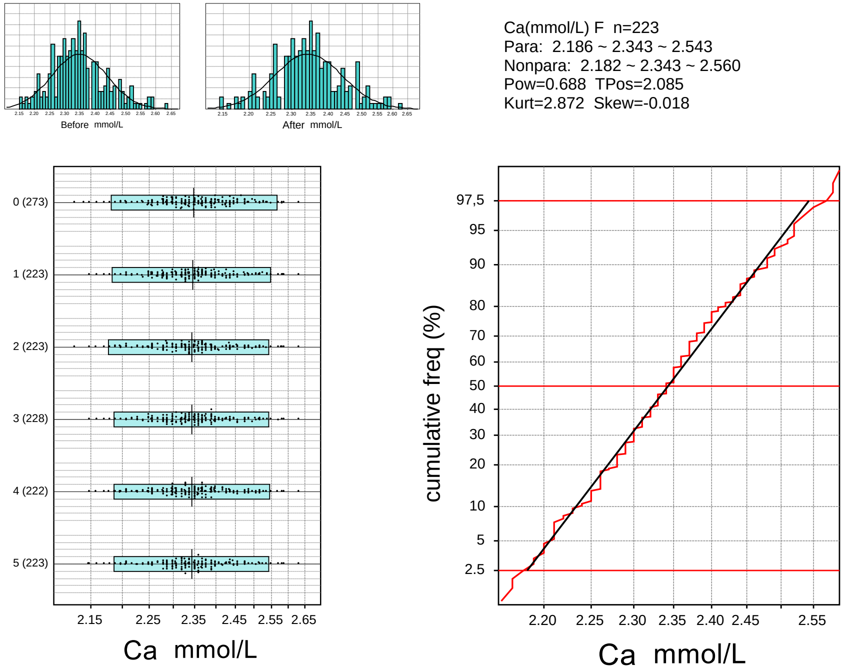
Before mmol/L
After mmol/L
Ca(mmol/L) F n=223
Para: 2.186 ~ 2.343 ~ 2.543
Nonpara: 2.182 ~ 2.343 ~ 2.560
Pow=0.688 TPos=2.085
Kurt=2.872 Skew=-0.018
0 (273)
1 (223)
2 (223)
3 (228)
4 (222)
5 (223)
Ca mmol/L
cumulative freq (%)
Ca mmol/L

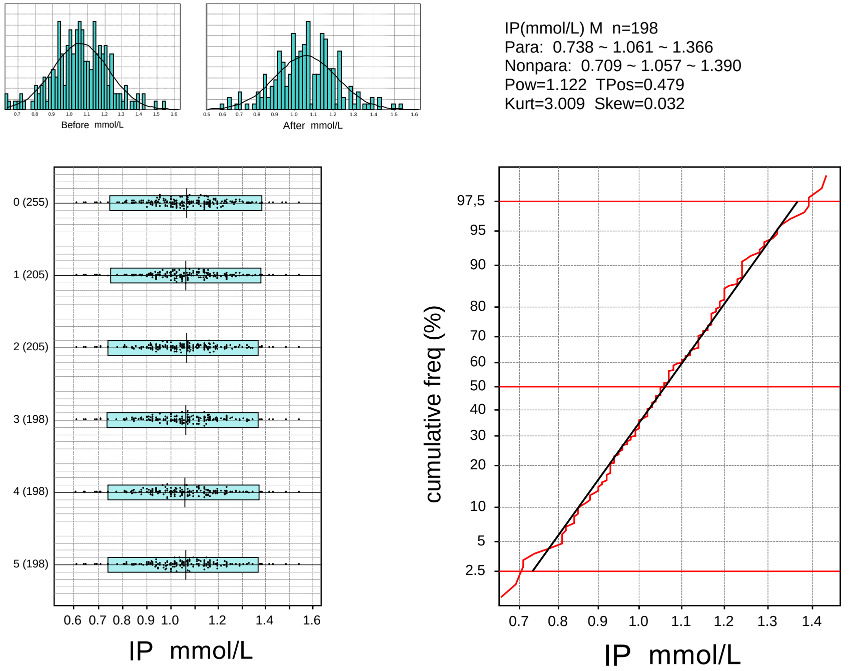
Before mmol/L
After mmol/L
IP(mmol/L) M n=198
Para: 0.738 ~ 1.061 ~ 1.366
Nonpara: 0.709 ~ 1.057 ~ 1.390
Pow=1.122 TPos=0.479
Kurt=3.009 Skew=0.032
0 (255)
1 (205)
2 (205)
3 (198)
4 (198)
5 (198)
IP mmol/L
cumulative freq (%)
IP mmol/L

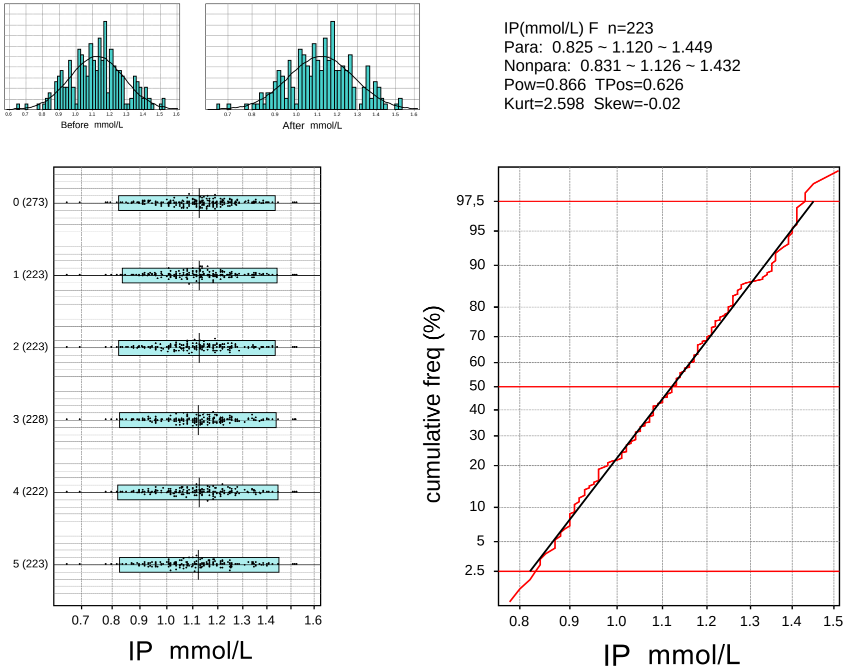
Before mmol/L
After mmol/L
IP(mmol/L) F n=223
Para: 0.825 ~ 1.120 ~ 1.449
Nonpara: 0.831 ~ 1.126 ~ 1.432
Pow=0.866 TPos=0.626
Kurt=2.598 Skew=-0.02
0 (273)
1 (223)
2 (223)
3 (228)
4 (222)
5 (223)
IP mmol/L
cumulative freq (%)
IP mmol/L

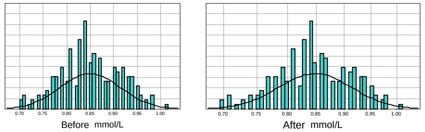

Mg(mmol/L) M n=198
Para: 0.733 ~ 0.850 ~ 0.981
Nonpara: 0.725 ~ 0.849 ~ 0.971
Pow=0.841 TPos=0.643
Kurt=2.534 Skew=-0.114

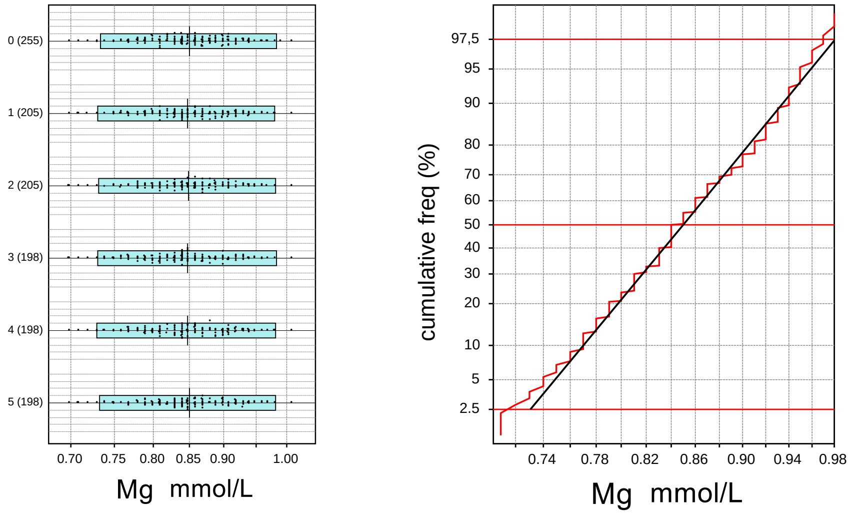

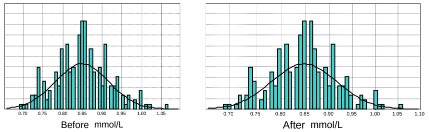

Mg(mmol/L) F n=223
Para: 0.723 ~ 0.848 ~ 0.984
Nonpara: 0.726 ~ 0.850 ~ 0.995
Pow=0.892 TPos=0.63
Kurt=2.726 Skew=-0.051

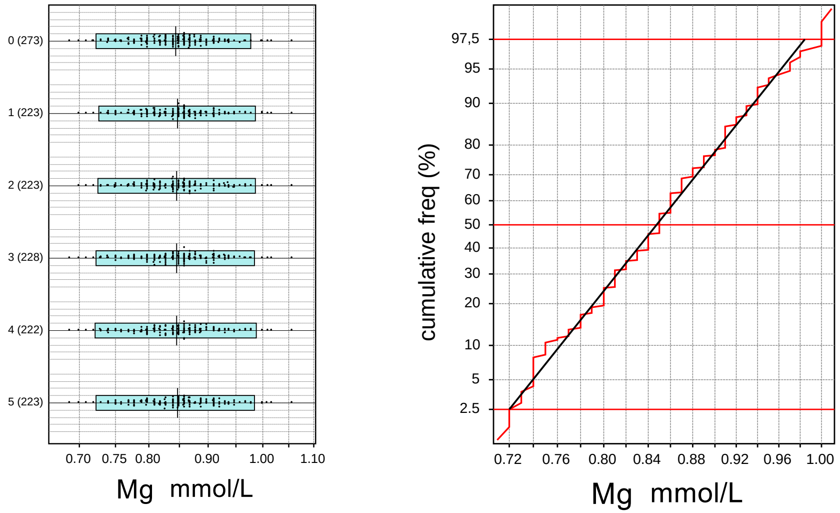

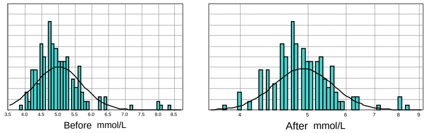

Glu(mmol/L) M n=193
Para: 4.06 ~ 4.89 ~ 6.52
Nonpara: 4.03 ~ 4.89 ~ 7.76
Pow=0.395 TPos=3.729
Kurt=2.811 Skew=-0.037

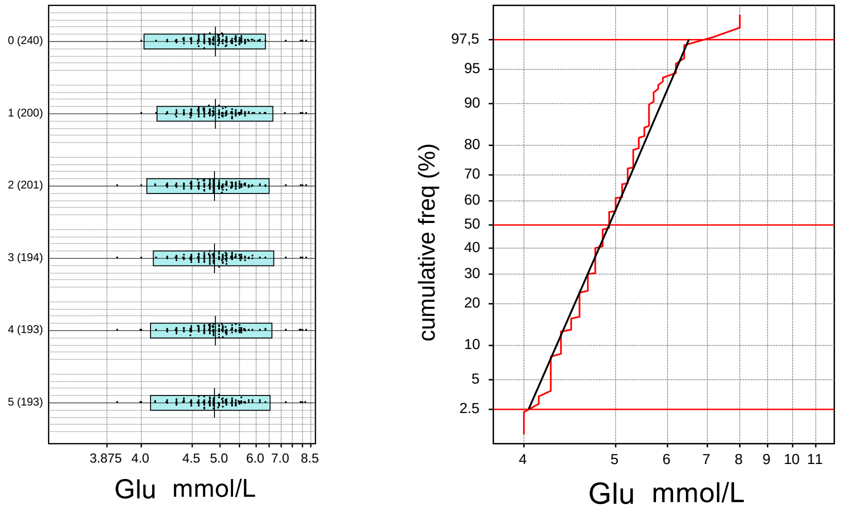

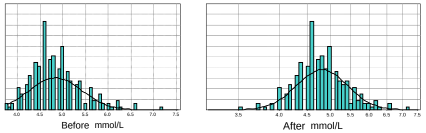

Glu(mmol/L) F n=228
Para: 3.89 ~ 4.80 ~ 6.09
Nonpara: 3.86 ~ 4.80 ~ 7.91
Pow=0.575 TPos=3.343
Kurt=2.826 Skew=0.127

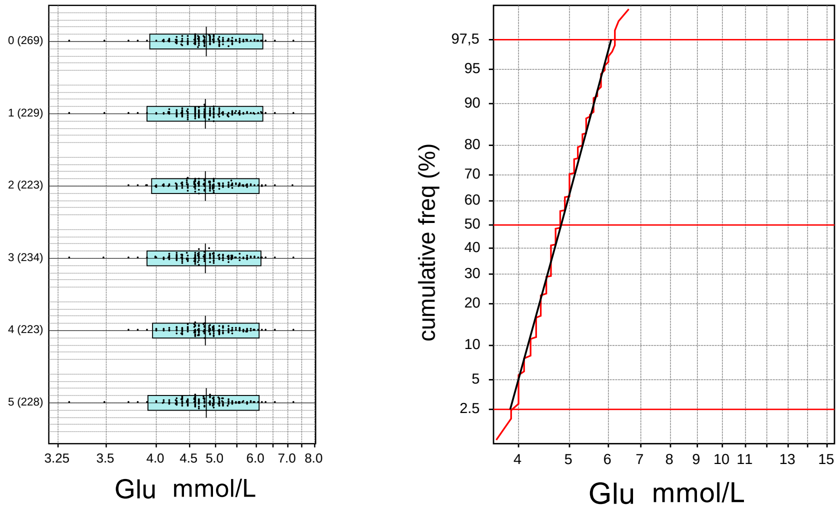

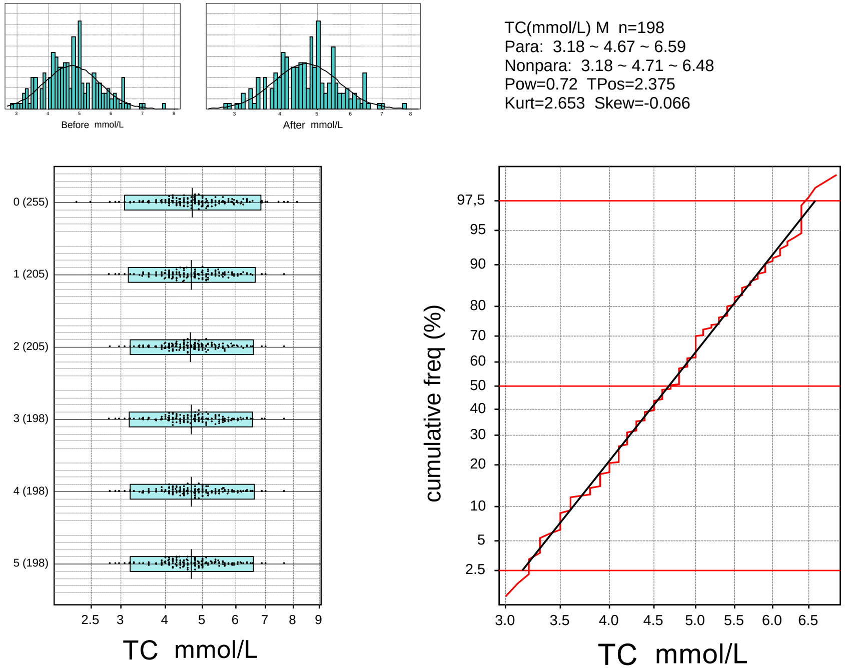

TC(mmol/L) M n=198
Para: 3.18 ~ 4.67 ~ 6.59
Nonpara: 3.18 ~ 4.71 ~ 6.48
Pow=0.72 TPos=2.375
Kurt=2.653 Skew=-0.066

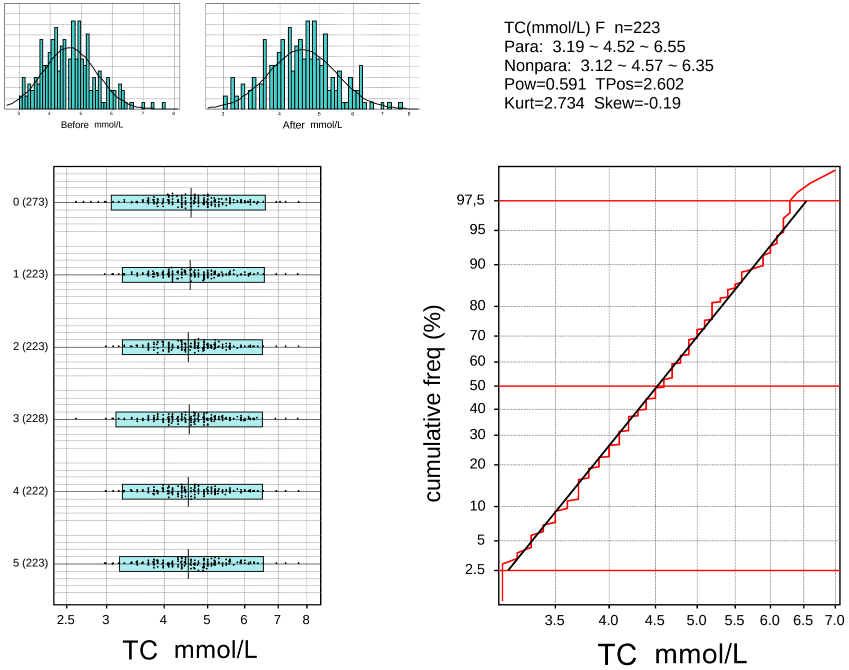

TC(mmol/L) F n=223
Para: 3.19 ~ 4.52 ~ 6.55
Nonpara: 3.12 ~ 4.57 ~ 6.35
Pow=0.591 TPos=2.602
Kurt=2.734 Skew=-0.19

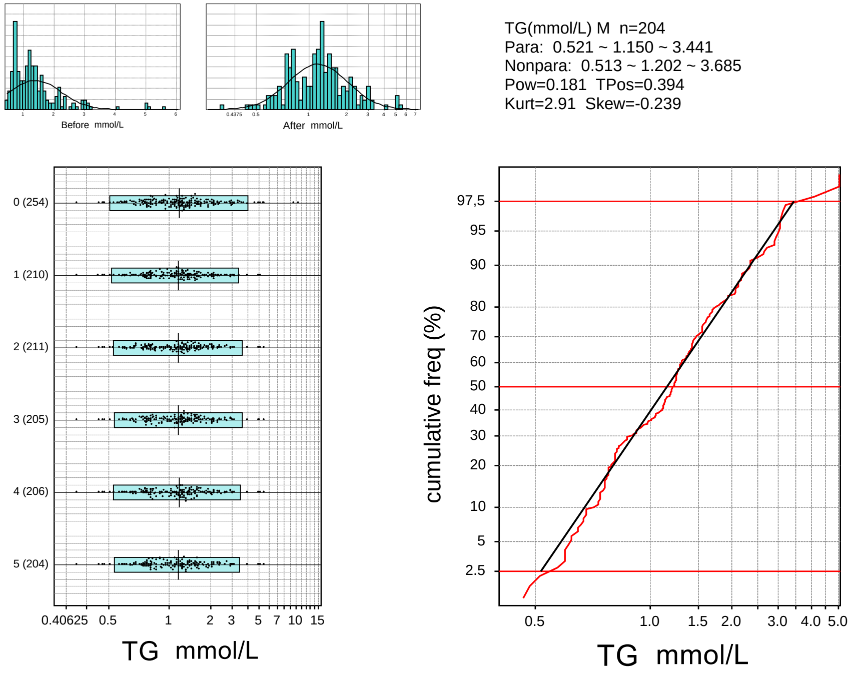

TG(mmol/L) M n=204
Para: 0.521 ~ 1.150 ~ 3.441
Nonpara: 0.513 ~ 1.202 ~ 3.685
Pow=0.181 TPos=0.394
Kurt=2.91 Skew=-0.239

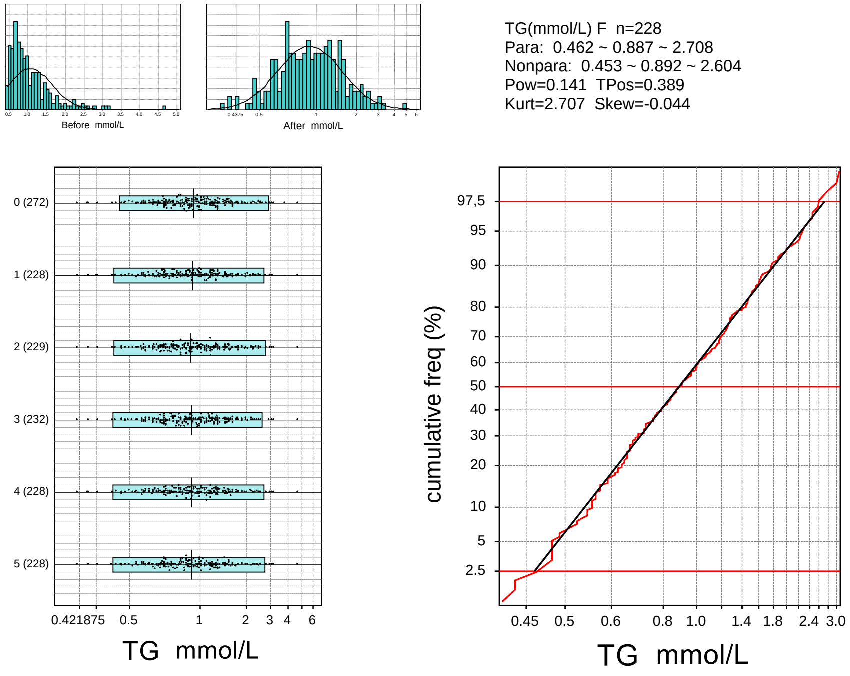

TG(mmol/L) F n=228
Para: 0.462 ~ 0.887 ~ 2.708
Nonpara: 0.453 ~ 0.892 ~ 2.604
Pow=0.141 TPos=0.389
Kurt=2.707 Skew=-0.044

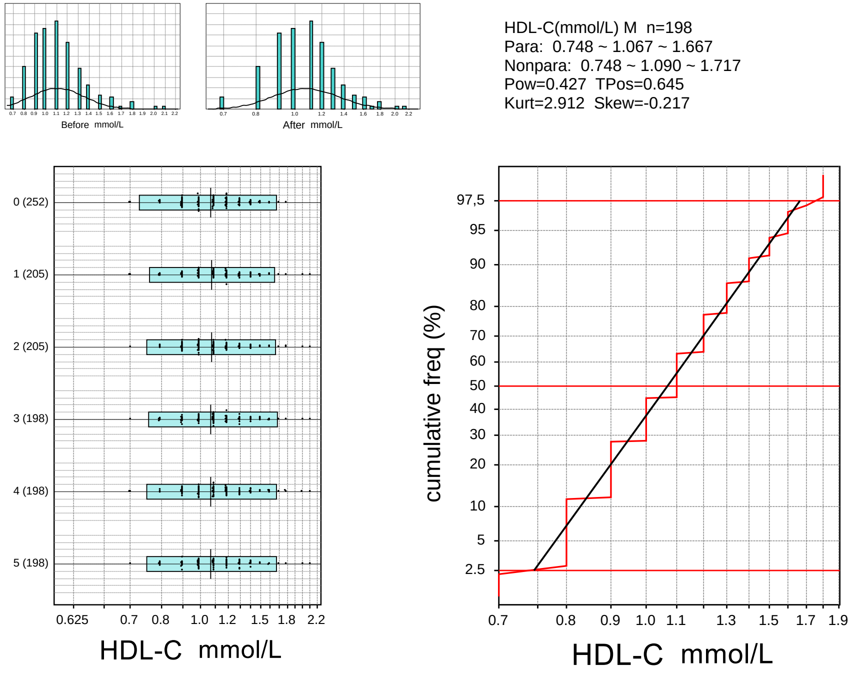
HDL-C(mmol/L) M n=198
Para: 0.748 ~ 1.067 ~ 1.667
Nonpara: 0.748 ~ 1.090 ~ 1.717
Pow=0.427 TPos=0.645
Kurt=2.912 Skew=-0.217
Before mmol/L
After mmol/L
0 (252)
1 (205)
2 (205)
3 (198)
4 (198)
5 (198)
HDL-C mmol/L
cumulative freq (%)
HDL-C mmol/L

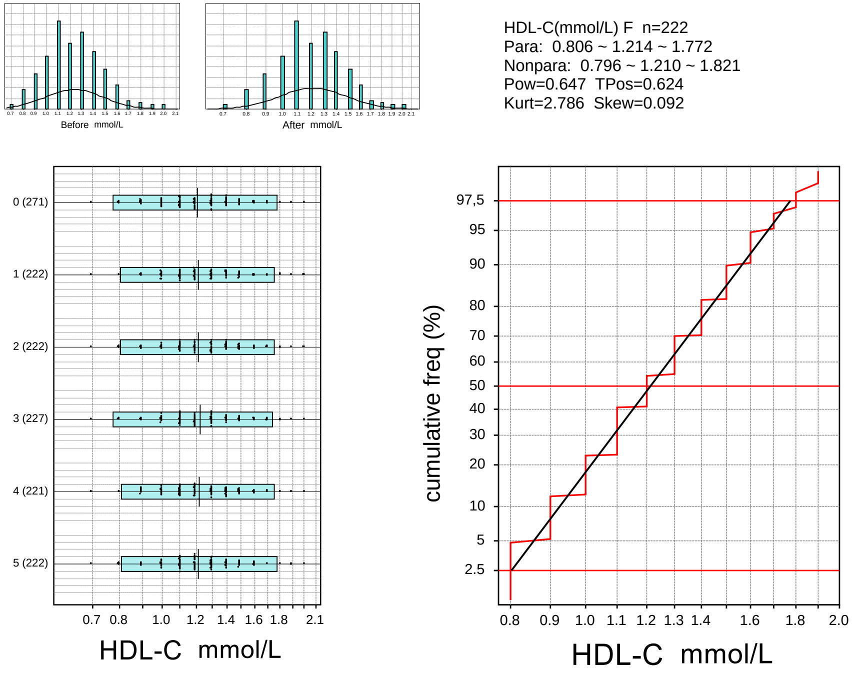
HDL-C(mmol/L) F n=222
Para: 0.806 ~ 1.214 ~ 1.772
Nonpara: 0.796 ~ 1.210 ~ 1.821
Pow=0.647 TPos=0.624
Kurt=2.786 Skew=0.092
Before mmol/L
After mmol/L
0 (271)
1 (222)
2 (222)
3 (227)
4 (221)
5 (222)
HDL-C mmol/L
cumulative freq (%)
HDL-C mmol/L

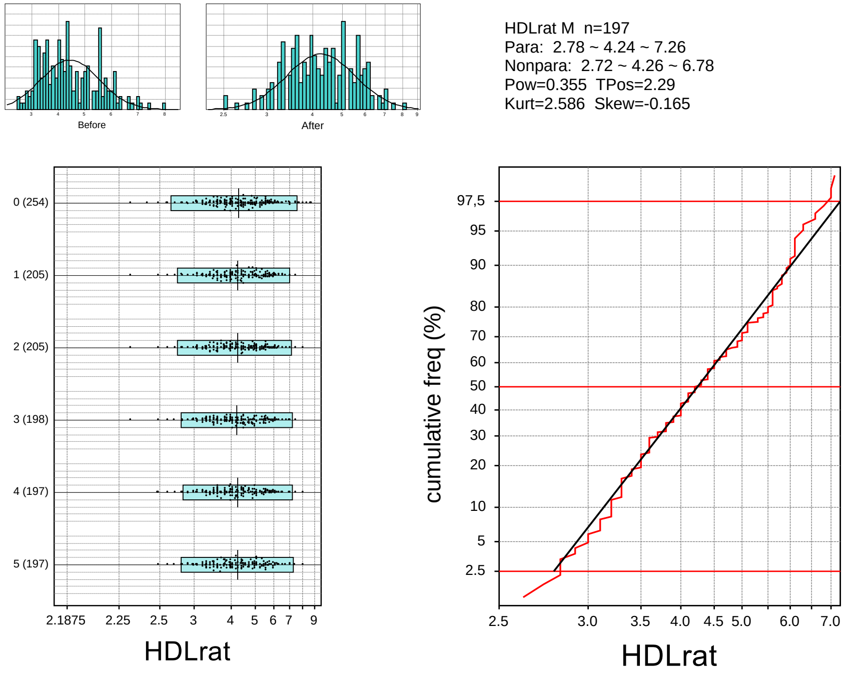
HDLrat M n=197
Para: 2.78 ~ 4.24 ~ 7.26
Nonpara: 2.72 ~ 4.26 ~ 6.78
Pow=0.355 TPos=2.29
Kurt=2.586 Skew=-0.165
Before
After
0 (254)
1 (205)
2 (205)
3 (198)
4 (197)
5 (197)
HDLrat
cumulative freq (%)
HDLrat

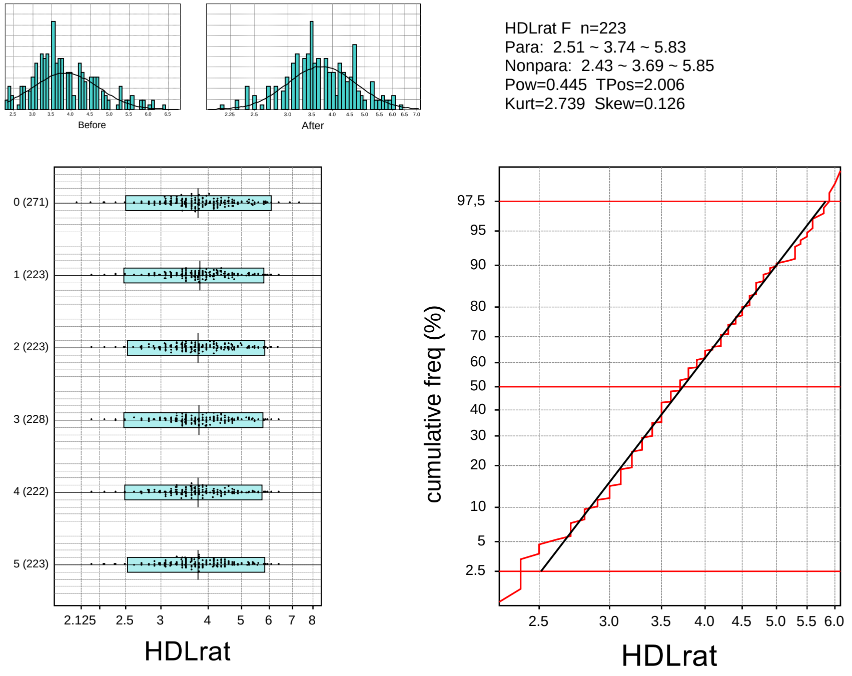
HDLrat F n=223
Para: 2.51 ~ 3.74 ~ 5.83
Nonpara: 2.43 ~ 3.69 ~ 5.85
Pow=0.445 TPos=2.006
Kurt=2.739 Skew=0.126
Before
After
0 (271)
1 (223)
2 (223)
3 (228)
4 (222)
5 (223)
HDLrat
cumulative freq (%)
HDLrat

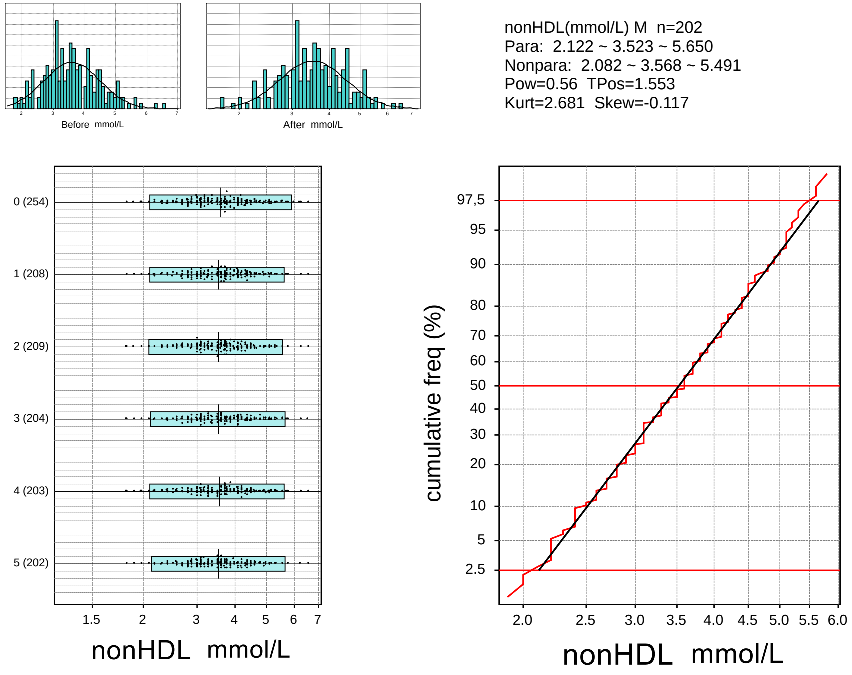
nonHDL(mmol/L) M n=202
Para: 2.122 ~ 3.523 ~ 5.650
Nonpara: 2.082 ~ 3.568 ~ 5.491
Pow=0.56 TPos=1.553
Kurt=2.681 Skew=-0.117
Before mmol/L
After mmol/L
0 (254)
1 (208)
2 (209)
3 (204)
4 (203)
5 (202)
nonHDL mmol/L
cumulative freq (%)
nonHDL mmol/L

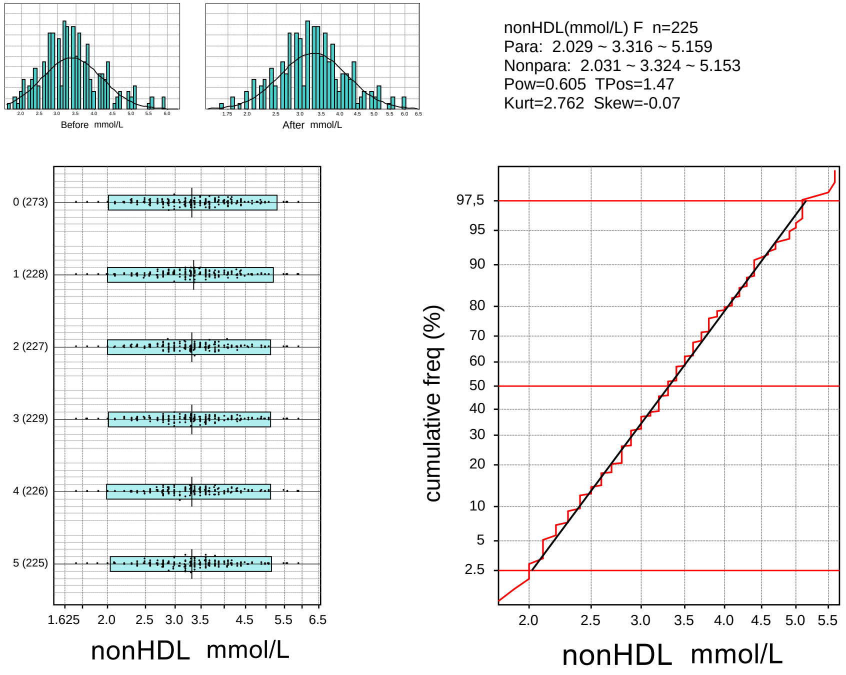
nonHDL(mmol/L) F n=225
Para: 2.029 ~ 3.316 ~ 5.159
Nonpara: 2.031 ~ 3.324 ~ 5.153
Pow=0.605 TPos=1.47
Kurt=2.762 Skew=-0.07
Before mmol/L
After mmol/L
0 (273)
1 (228)
2 (227)
3 (229)
4 (226)
5 (225)
nonHDL mmol/L
cumulative freq (%)
nonHDL mmol/L

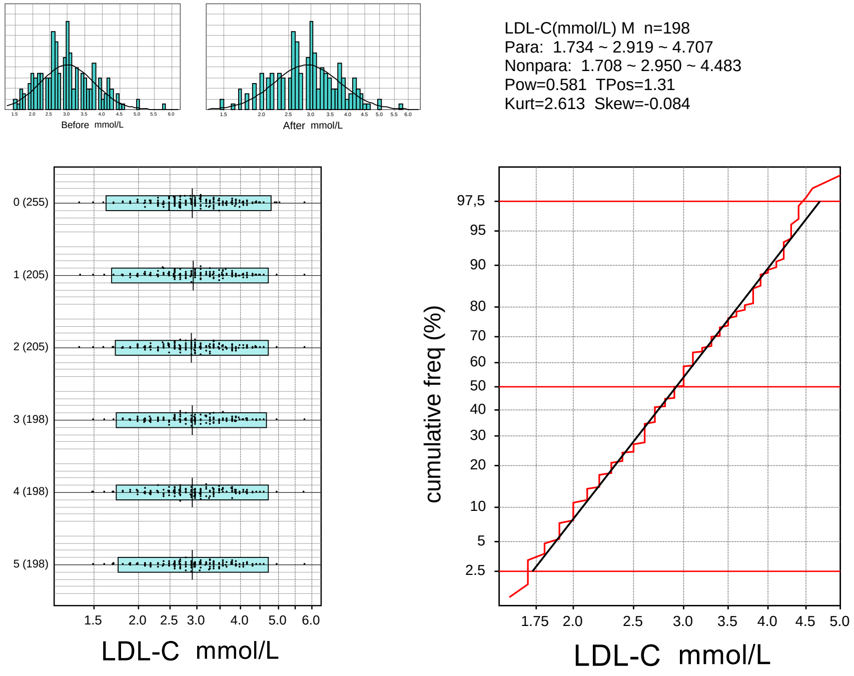
LDL-C(mmol/L) M n=198
Para: 1.734 ~ 2.919 ~ 4.707
Nonpara: 1.708 ~ 2.950 ~ 4.483
Pow=0.581 TPos=1.31
Kurt=2.613 Skew=-0.084
Before mmol/L
After mmol/L
0 (255)
1 (205)
2 (205)
3 (198)
4 (198)
5 (198)
LDL-C mmol/L
cumulative freq (%)
LDL-C mmol/L

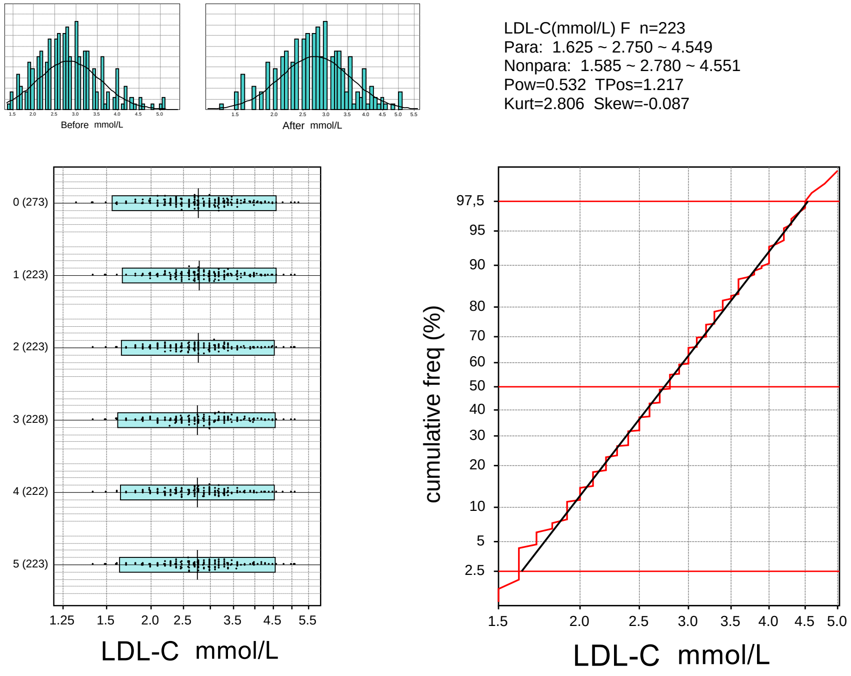
LDL-C(mmol/L) F n=223
Para: 1.625 ~ 2.750 ~ 4.549
Nonpara: 1.585 ~ 2.780 ~ 4.551
Pow=0.532 TPos=1.217
Kurt=2.806 Skew=-0.087
Before mmol/L
After mmol/L
0 (273)
1 (223)
2 (223)
3 (228)
4 (222)
5 (223)
LDL-C mmol/L
cumulative freq (%)
LDL-C mmol/L

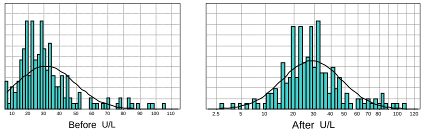

Lip(U/L) M n=195
Para: 7.57 ~ 28.49 ~ 72.33
Nonpara: 7.53 ~ 27.70 ~ 97.21
Pow=0.416 TPos=1.988
Kurt=3.039 Skew=0.159

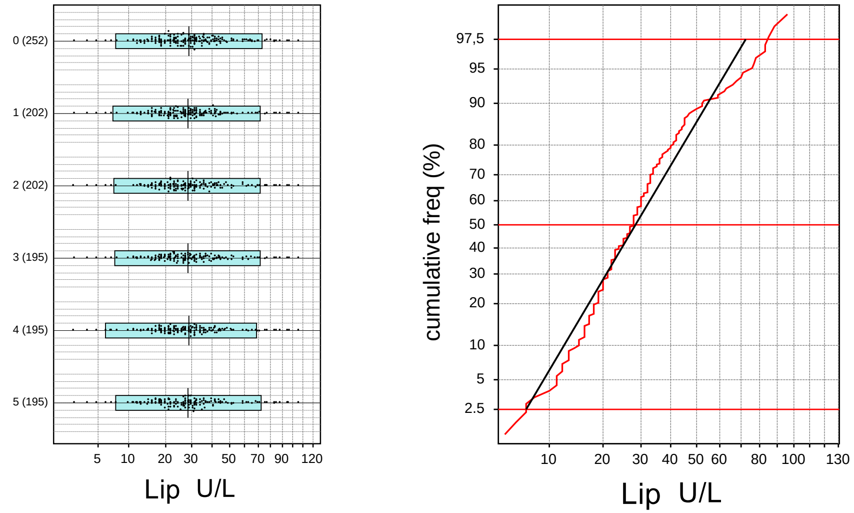

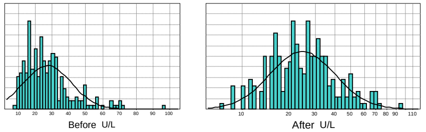

Lip(U/L) F n=222
Para: 9.63 ~ 24.24 ~ 60.48
Nonpara: 9.65 ~ 24.30 ~ 65.26
Pow=0.283 TPos=5.531
Kurt=2.797 Skew=0.002

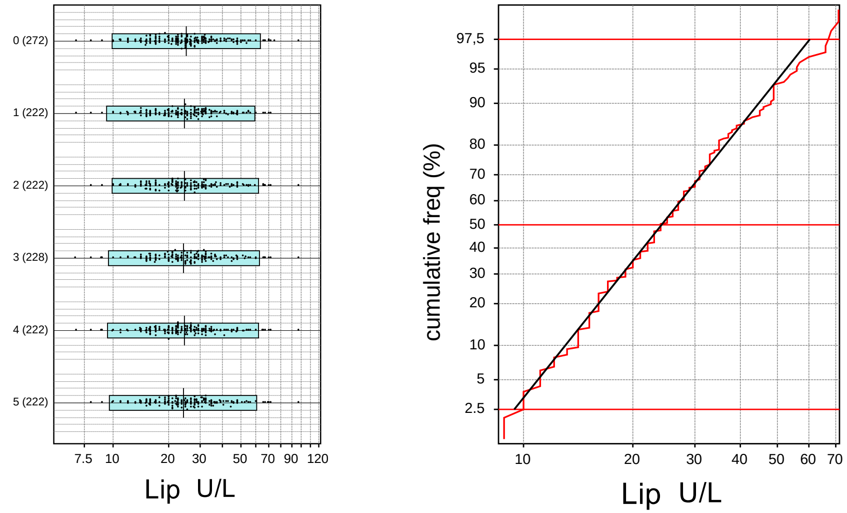

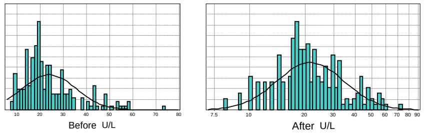

ALT(U/L) M n=202
Para: 9.59 ~ 21.57 ~ 53.77
Nonpara: 9.52 ~ 20.90 ~ 56.15
Pow=0.234 TPos=6.165
Kurt=2.755 Skew=0.266

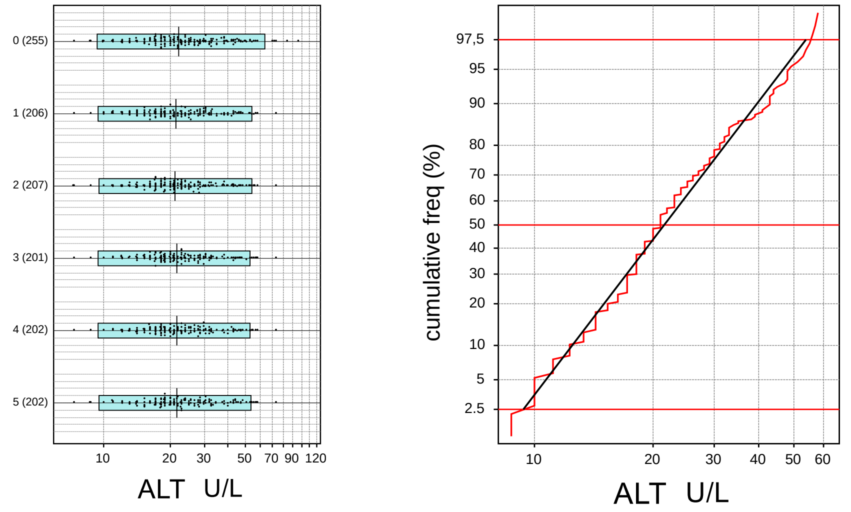

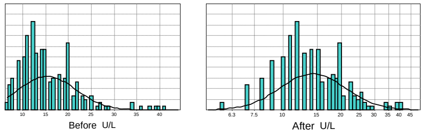

ALT(U/L) F n=233
Para: 7.20 ~ 14.14 ~ 30.73
Nonpara: 7.18 ~ 14.00 ~ 34.93
Pow=0.279 TPos=4.991
Kurt=2.89 Skew=0.192

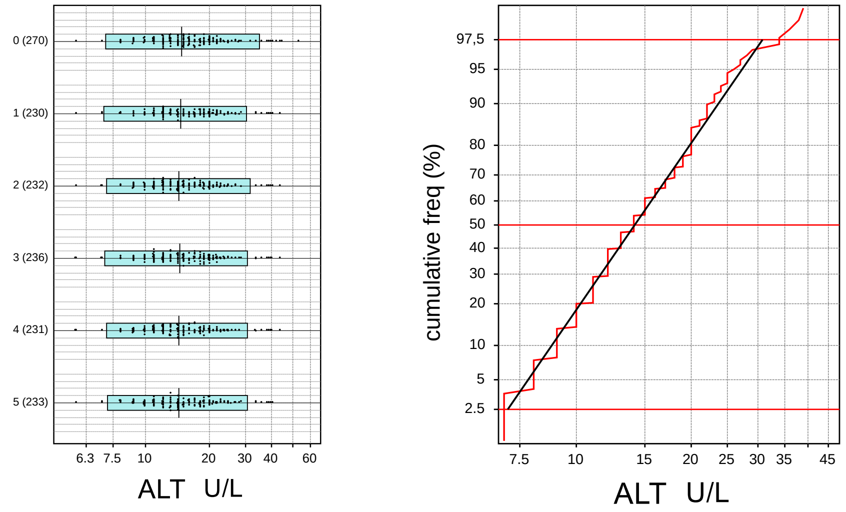

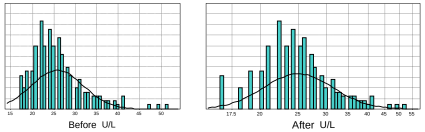

AST(U/L) M n=206
Para: 17.39 ~ 24.76 ~ 39.82
Nonpara: 17.09 ~ 24.60 ~ 41.16
Pow=0.361 TPos=14.75
Kurt=2.841 Skew=0.077

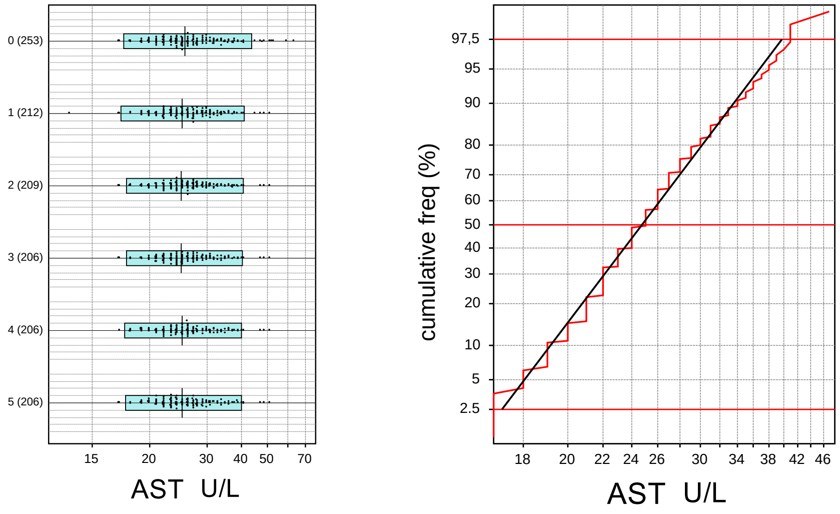

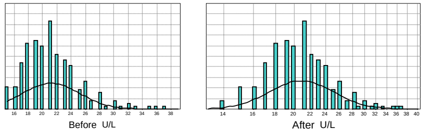

AST(U/L) F n=231
Para: 14.89 ~ 20.75 ~ 29.99
Nonpara: 14.87 ~ 20.80 ~ 32.28
Pow=0.527 TPos=12.394
Kurt=2.851 Skew=-0.003

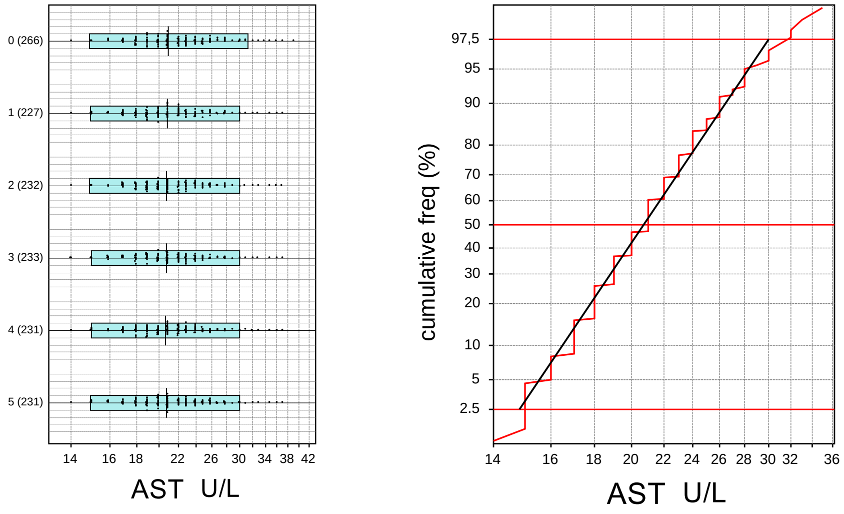

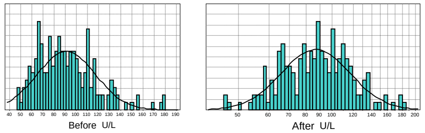

ALP(U/L) M n=198
Para: 52.9 ~ 86.9 ~ 154.7
Nonpara: 53.2 ~ 87.9 ~ 156.5
Pow=0.41 TPos=43.238
Kurt=2.828 Skew=-0.074

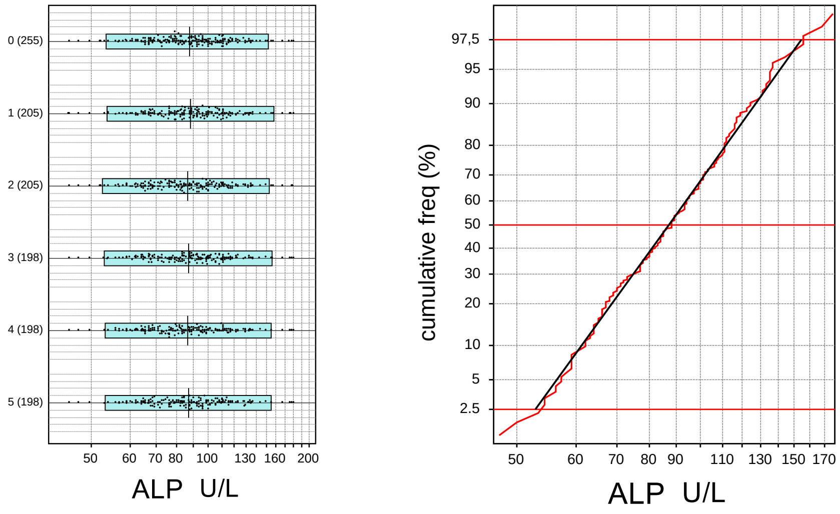

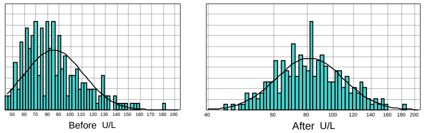

ALP(U/L) F n=221
Para: 47.9 ~ 80.7 ~ 145.7
Nonpara: 46.9 ~ 81.0 ~ 144.0
Pow=0.382 TPos=36.188
Kurt=2.629 Skew=-0.003

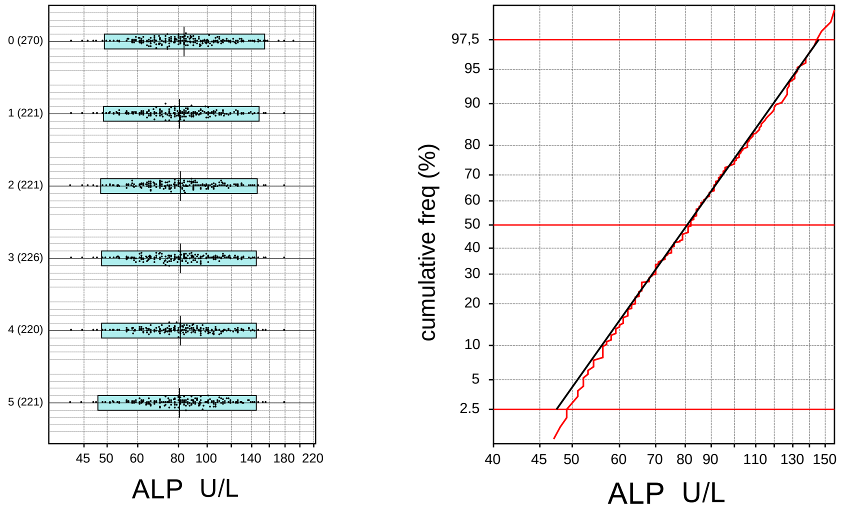

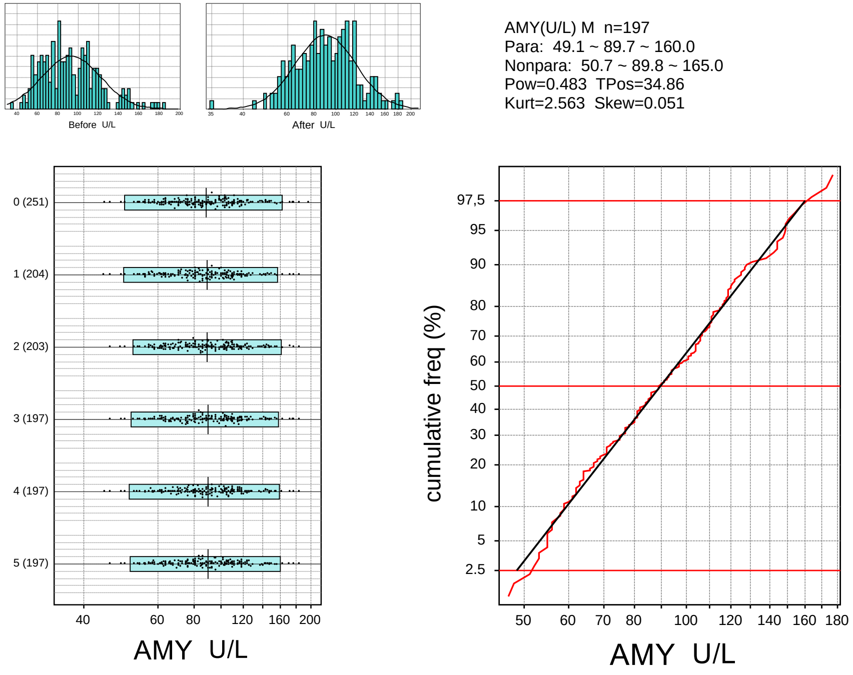
Before U/L
After U/L
AMY(U/L) M n=197
Para: 49.1 ~ 89.7 ~ 160.0
Nonpara: 50.7 ~ 89.8 ~ 165.0
Pow=0.483 TPos=34.86
Kurt=2.563 Skew=0.051
0 (251)
1 (204)
2 (203)
3 (197)
4 (197)
5 (197)
AMY U/L
cumulative freq (%)
AMY U/L

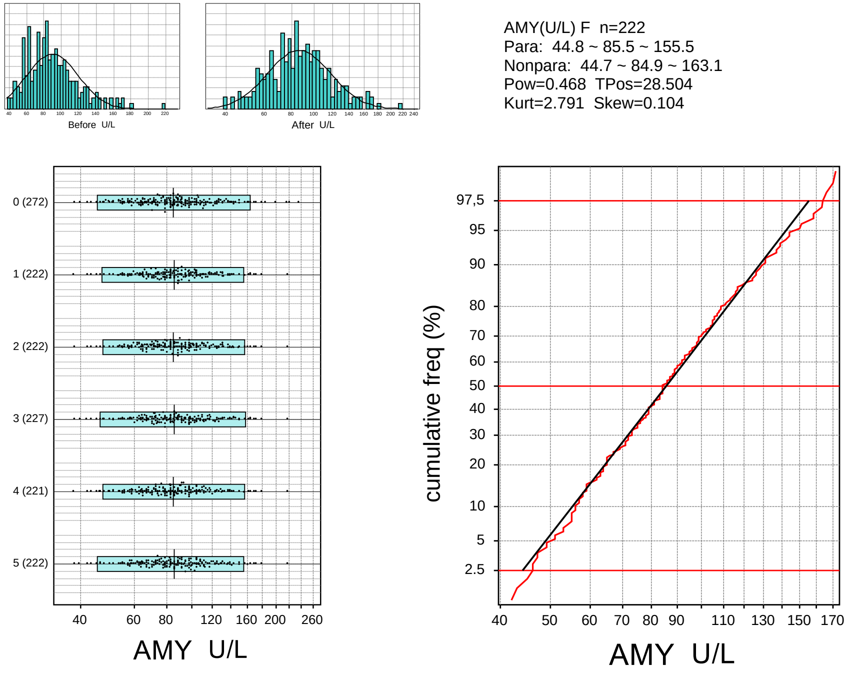
Before U/L
After U/L
AMY(U/L) F n=222
Para: 44.8 ~ 85.5 ~ 155.5
Nonpara: 44.7 ~ 84.9 ~ 163.1
Pow=0.468 TPos=28.504
Kurt=2.791 Skew=0.104
0 (272)
1 (222)
2 (222)
3 (227)
4 (221)
5 (222)
AMY U/L
cumulative freq (%)
AMY U/L

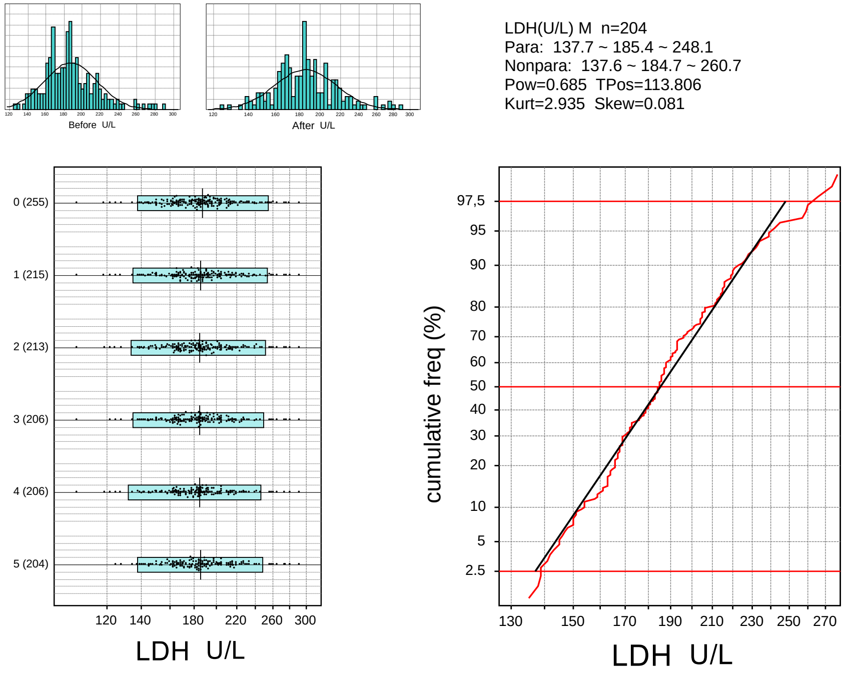
Before U/L
After U/L
LDH(U/L) M n=204
Para: 137.7 ~ 185.4 ~ 248.1
Nonpara: 137.6 ~ 184.7 ~ 260.7
Pow=0.685 TPos=113.806
Kurt=2.935 Skew=0.081
0 (255)
1 (215)
2 (213)
3 (206)
4 (206)
5 (204)
LDH U/L
cumulative freq (%)
LDH U/L

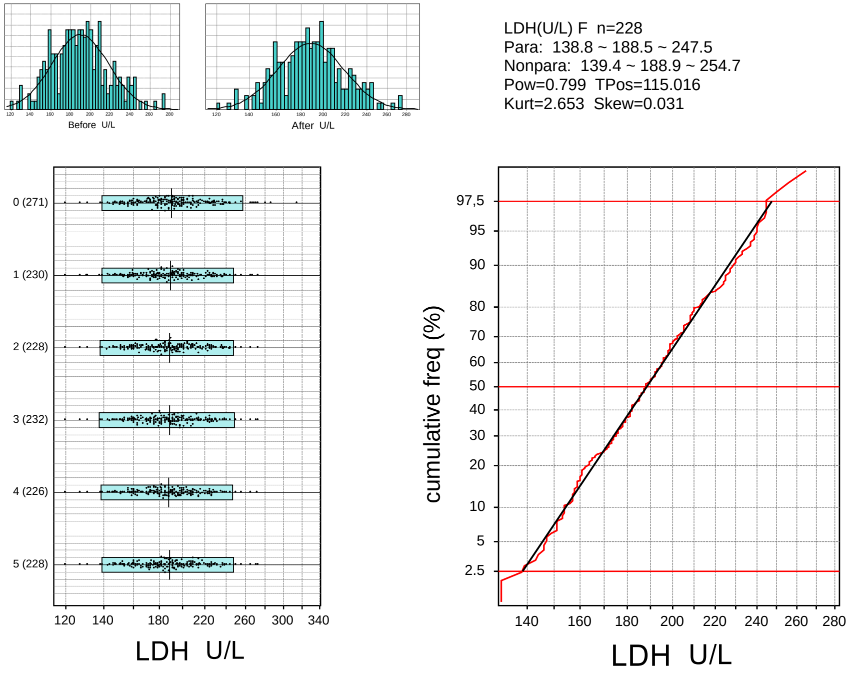
Before U/L
After U/L
LDH(U/L) F n=228
Para: 138.8 ~ 188.5 ~ 247.5
Nonpara: 139.4 ~ 188.9 ~ 254.7
Pow=0.799 TPos=115.016
Kurt=2.653 Skew=0.031
0 (271)
1 (230)
2 (228)
3 (232)
4 (226)
5 (228)
LDH U/L
cumulative freq (%)
LDH U/L

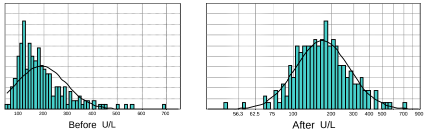

CK(U/L) M n=204
Para: 74.0 ~ 168.6 ~ 426.0
Nonpara: 73.6 ~ 167.5 ~ 460.7
Pow=0.274 TPos=53.065
Kurt=2.748 Skew=0.081

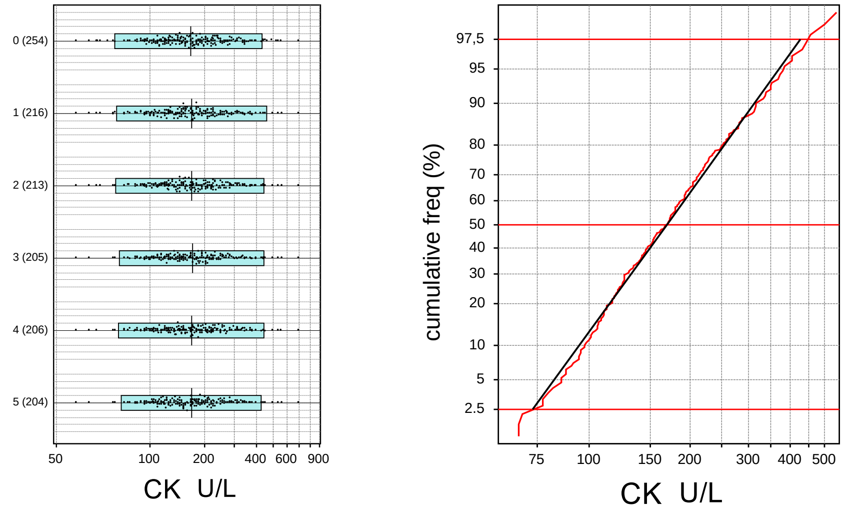

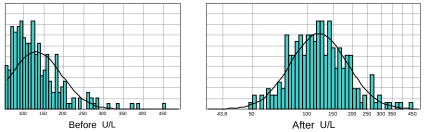

CK(U/L) F n=223
Para: 55.1 ~ 118.8 ~ 274.6
Nonpara: 55.7 ~ 118.0 ~ 336.8
Pow=0.361 TPos=43.399
Kurt=2.829 Skew=0.118

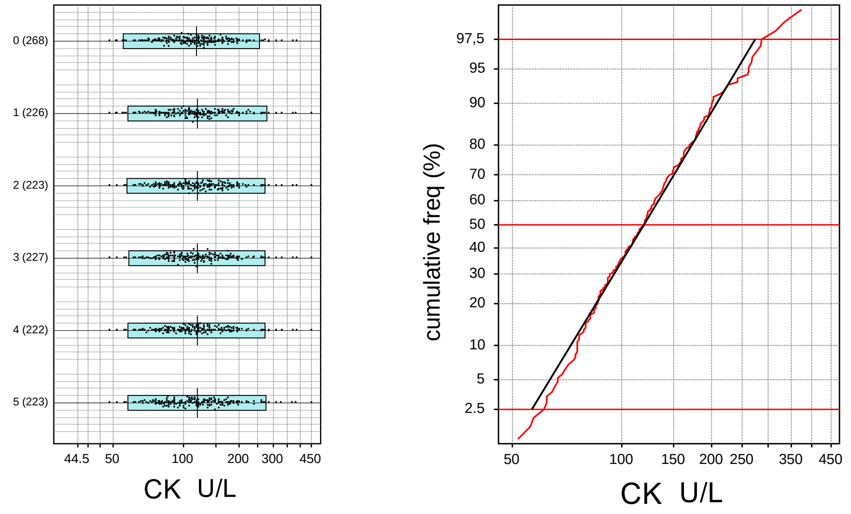

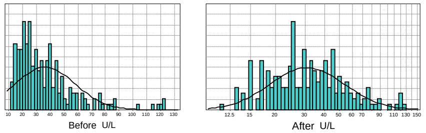

GGT(U/L) M n=200
Para: 12.87 ~ 30.65 ~ 91.30
Nonpara: 13.57 ~ 30.40 ~ 103.46
Pow=0.209 TPos=9.62
Kurt=2.688 Skew=0.096

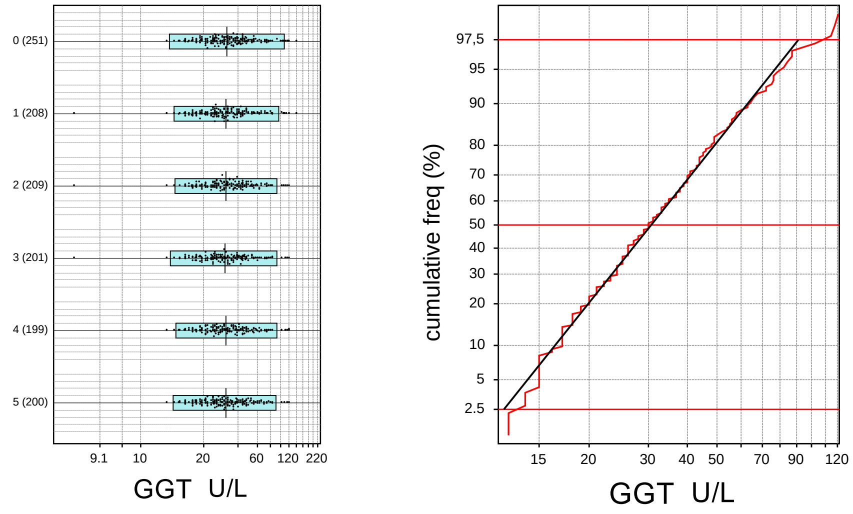

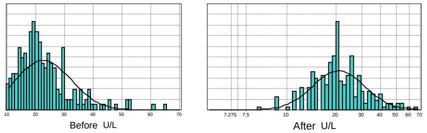

GGT(U/L) F n=227
Para: 10.14 ~ 21.08 ~ 47.20
Nonpara: 9.81 ~ 20.50 ~ 52.88
Pow=0.316 TPos=7.203
Kurt=2.906 Skew=0.173

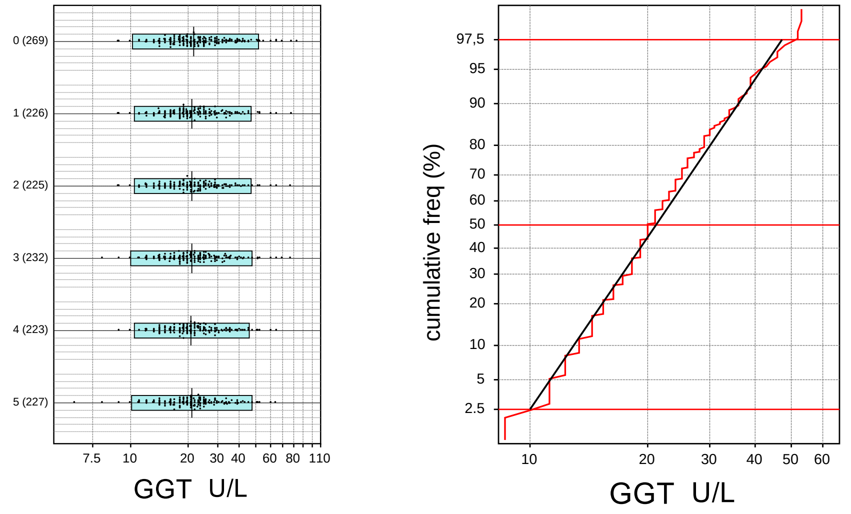

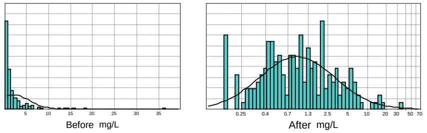

CRP(mg/L) M n=203
Para: 0.20 ~ 0.92 ~ 11.94
Nonpara: 0.20 ~ 0.92 ~ 11.64
Pow=-0.011 TPos=0.139
Kurt=2.701 Skew=-0.085

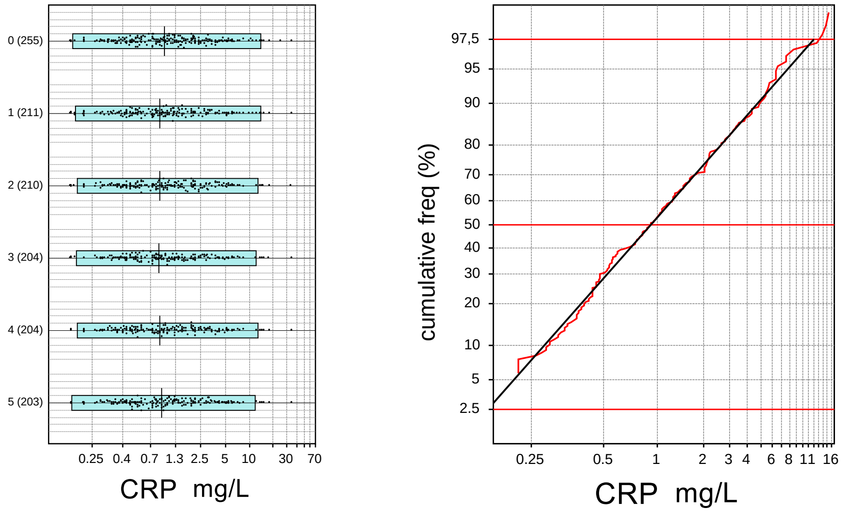

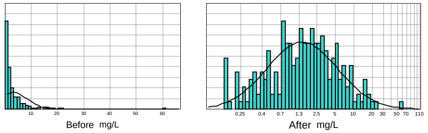

CRP(mg/L) F n=226
Para: 0.22 ~ 1.48 ~ 20.11
Nonpara: 0.21 ~ 1.60 ~ 16.47
Pow=0.031 TPos=0.153
Kurt=2.603 Skew=-0.208

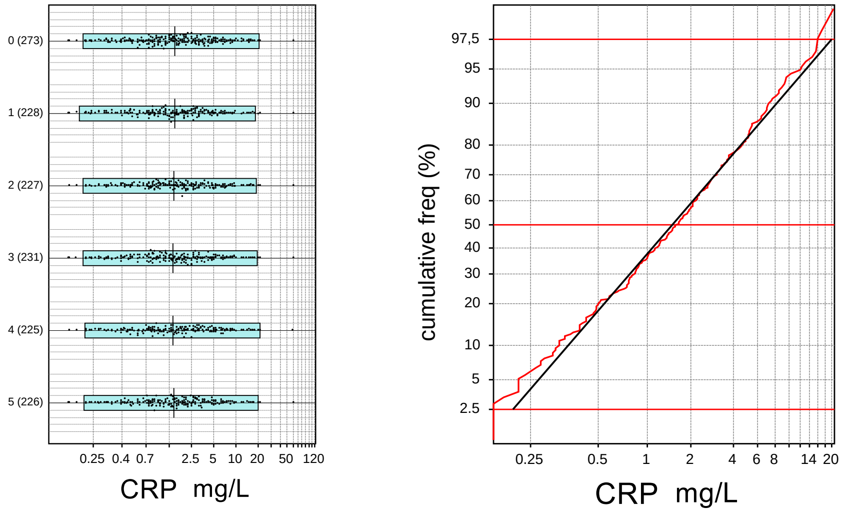

Supplement: S3 Fig — The accuracy of Gaussian transformation by Box-Cox formula can be assessed from theoretical Gaussian curves in two histograms shown on left top (before and after the transformation) of each panel. Accuracy can be also seen from the linearity in probability paper plot on the right. The limits of the RI by nonparametric method corresponds to the points where red zigzag line intersect with horizontal 2.5 and 97.5% red lines of cumulative frequencies. (PDF) [file pone.0235234.s003.pdf]
