# Supplementary material for: Determination of reference intervals for common chemistry and immunoassay tests for Kenyan adults based on an internationally harmonized protocol and up-to-date statistical methods
Source: PLoS One. 2020 Jul 9;15(7):e0235234. doi: 10.1371/journal.pone.0235234 (PMC7347104; doi:10.1371/journal.pone.0235234)
Supplement: S1 Table — (PDF) [file pone.0235234.s005.pdf]

S1 Table. Reference intervals and determination of method, sex and age bias

Item	Units	RU	SDR sex	SDR age		LAVE criteria	LAVE	Age	RIs by parametric method												Item	LAVE effect on LL			LAVE effect on UL			btw-sex diff		btw-age diff		btw-age diff		Decision
				M	F				M + F				M				F					M+F	M	F	M+F	M	F			M		F		
									n	LL	Me	UL	n	LL	Me	UL	n	LL	Me	UL		BR_{LL}			BR_{UL}			BR_{LL}	BR_{UL}	BR_{LL}	BR_{UL}	BR_{LL}	BR_{UL}	
**TP**	**g/L**	1	0.00	0.38	0.35	2	(-)	All	526	67	74	83	254	68	74	83	271	66	74	84	**TP**							**0.49**	-0.25					No partition by sex/age, LAVE-
						2	(+)		414	67	74	82	195	68	74	82	215	66	74	82		0.00	0.00	0.00	0.26	0.28	**0.49**	**0.52**	0.00					
**Alb**	**g/L**	1	**0.52**	**0.55**	**0.52**	2	(-)	All	527	38	44	50	255	40	45	51	272	38	43	48	**Alb**							**0.65**	**0.98**					Partition by age, LAVE- for < 45 and LAVE + > 45
						2	(+)		423	39	44	50	197	41	45	50	216	38	43	49		-0.36	**-0.39**	0.00	0.00	0.00	-0.36	**1.07**	**0.71**					
						2	(-)	~45	349	39	44	50	176	40	45	51	173	38	43	49								**0.71**	**0.71**	0.00	**0.71**	0.00	**0.78**	
						2	(+)		292	39	44	50	148	40	45	51	145	38	43	49		0.00	0.00	0.00	0.00	0.00	0.00	**0.71**	**0.71**	0.00	**1.57**	0.00	**1.07**	
						2	(-)	45~	183	38	43	48	84	40	44	49	99	38	42	47								**0.78**	**0.78**					
						2	(+)		150	39	43	47	66	40	43	47	83	38	42	46		**-0.49**	0.00	0.00	**0.49**	**1.12**	**0.49**	**0.98**	**0.49**					
**Glb**	**g/L**	1	0.34	0.00	0.00	2	(-)	All	524	24	30	38	255	24	29	36	270	24	31	39	**Glb**							0.00	**-0.84**					No partition by sex/age. LAVE-
						2	(+)		435	24	30	37	204	24	29	36	221	24	31	37		0.00	0.00	0.00	0.30	0.00	**0.60**	0.00	-0.30					
**Urea**	**mmol/L**	0.1	0.30	0.22	**0.41**	1	(-)	All	530	2.0	3.5	6.1	258	2.4	3.7	6.6	272	1.9	3.2	5.8	**Urea**							**0.48**	**0.76**					Partition by age for female. LAVE-
						1	(+)		395	2.0	3.4	6.0	198	2.3	3.6	6.3	218	1.9	3.2	5.9		0.00	0.10	0.00	0.10	0.29	-0.10	**0.39**	**0.39**					
						1	(-)	~45	349	2.0	3.3	5.8	175	2.4	3.6	6.5	174	1.8	3.1	5.3								**0.62**	**1.24**	0.00	**-0.47**	-0.10	**-0.90**	
						1	(+)		284	2.0	3.3	5.6	145	2.4	3.5	6.3	145	1.8	3.0	5.2		0.00	0.00	0.00	0.22	0.20	0.12	**0.65**	**1.20**	0.10	0.00	0.00	**-1.18**	
						1	(-)	45~	182	2.1	3.7	6.6	84	2.4	3.9	7.0	99	1.9	3.6	6.2								**0.44**	**0.70**					
						1	(+)		149	2.1	3.7	6.4	66	2.3	3.8	6.3	83	1.8	3.6	6.4		0.00	0.10	0.09	0.18	**0.69**	-0.17	**0.46**	-0.09					
**UA**	**mmol/L**	1	**0.94**	0.39	0.31	1	(-)	All	532	183	308	489	259	238	350	532	272	178	268	417	**UA**							**0.77**	**1.47**					Partition by sex. LAVE-
						1	(+)		407	184	297	459	204	243	349	507	221	178	264	403		-0.01	-0.07	0.00	**0.43**	0.37	0.24	**0.93**	**1.48**					
**Cre**	**μmol/L**	1	**1.21**	0.27	0.14	1	(-)	All	531	47	70	108	260	58	80	109	271	45	61	86	**Cre**							**0.84**	**1.48**					Partition by sex. Use LAVE-
						1	(+)		394	46	69	105	198	58	81	108	218	44	61	84		0.07	0.00	0.10	0.20	0.08	0.20	**0.93**	**1.59**					
**TBil**	**μmol/L**	1	**0.48**	0.21	0.30	1	(-)	All	530	5	11	35	257	6	13	43	271	5	9	27	**TBil**							0.13	**2.09**					Partition by sex. Use LAVE-
						1	(+)		394	5	11	35	197	6	13	40	217	5	10	26		0.00	0.00	0.00	0.00	0.35	0.19	0.13	**1.83**					
**Na**	**mmol/L**	1	0.00	0.09	**0.49**	1	(-)	All	531	134	138	142	258	135	138	141	272	134	137	142	**Na**							**0.49**	**-0.49**					No partiion by sex/age. with actual bias by age in females ≤2RU. LAVE -
						1	(+)		394	134	138	142	197	135	138	141	218	134	137	142		0.00	0.00	0.00	0.00	0.00	0.00	**0.49**	**-0.49**					
						1	(-)	~45	348	134	137	141	175	135	138	141	173	134	137	141								**0.52**	-0.03	**0.54**	-0.29	-0.05	**-0.65**	
						1	(+)		283	134	138	141	144	135	138	141	144	134	137	141		-0.15	-0.06	-0.12	-0.06	0.06	0.03	**0.46**	-0.05	0.33	-0.11	0.05	**-0.65**	
						1	(-)	45~	183	134	138	142	84	134	138	142	99	134	138	143								0.00	**-0.46**					
						1	(+)		149	134	138	142	66	134	138	141	83	134	138	143		0.05	-0.24	0.00	0.08	0.21	0.02	0.20	**-0.61**					
**K**	**mmol/L**	0.1	0.00	0.20	0.22	1	(-)	All	531	3.4	3.9	4.8	259	3.3	3.9	4.9	273	3.5	4.0	4.8	**K**							**-0.49**	0.24					No partiion by sex/age. LAVE-
						1	(+)		394	3.4	3.9	4.8	198	3.4	3.9	4.9	218	3.5	4.0	4.8		0.00	-0.23	0.00	0.00	0.00	0.00	-0.24	0.24					
**Cl**	**mmol/L**	1	**0.45**	**0.41**	0.21	1	(-)	All	531	100	105	110	258	100	104	109	272	101	106	110	**Cl**							**-0.39**	**-0.39**					No partiion by sex/age. LAVE- with actual bias by sex, age ≤2RU
						1	(+)		395	101	105	110	198	100	104	109	218	102	106	110		**-0.44**	0.00	**-0.49**	0.00	0.00	0.00	**-0.87**	**-0.44**					
						1	(-)	~45	349	100	105	109	173	101	105.7	110	134	100	104	108								0.10	**0.61**	**-0.62**	-0.34	**-0.71**	**-1.22**	
						1	(+)		183	101	106	110	99	102	106.2	111	65	101	106	109		-0.25	-0.13	-0.18	**-0.54**	**-0.52**	**-0.42**	0.22	0.37	-0.35	**-0.50**	**-0.60**	**-1.19**	
						1	(-)	45~	175	100	104	108	266	101	104.9	109	134	102	106	110								-0.03	**-0.38**					
						1	(+)		83	100	105	110	137	101	106	110	79	102	106	111		-0.13	-0.35	0.17	**-0.67**	**-0.52**	**-0.51**	-0.07	**-0.40**					
**Ca**	**mmol/L**	0.01	0.15	0.31	0.26	1	(-)	All	531	2.19	2.36	2.57	259	2.21	2.38	2.56	273	2.18	2.35	2.57	**Ca**							0.31	-0.10					No partiion by sex/age. LAVE-
						1	(+)		395	2.20	2.36	2.55	197	2.21	2.38	2.57	219	2.19	2.34	2.54		-0.11	0.00	-0.11	0.22	-0.11	0.34	0.22	0.34					
**IP**	**mmol/L**	0.01	0.21	0.22	0.16	1	(-)	All	533	0.78	1.10	1.42	260	0.75	1.07	1.38	273	0.82	1.12	1.44	**IP**							**-0.43**	-0.37					No partiion by sex/age. LAVE-
						1	(+)		396	0.77	1.09	1.43	198	0.74	1.06	1.36	219	0.82	1.12	1.44		0.06	0.06	0.00	-0.06	0.13	0.00	**-0.48**	**-0.48**					
**Mg**	**mmol/L**	0.01	0.00	0.37	**0.54**	1	(-)	All	533	0.73	0.85	0.98	260	0.73	0.85	0.98	273	0.72	0.85	0.98	**Mg**							0.16	0.00					Partition by age for female. LAVE-
						1	(+)		396	0.72	0.85	0.98	198	0.73	0.85	0.98	219	0.72	0.85	0.98		0.15	0.00	0.00	0.00	0.00	0.00	0.15	0.00					
						1	(-)	~45	350	0.72	0.83	0.95	176	0.72	0.83	0.97	174	0.71	0.83	0.93								0.10	**0.61**	**-0.62**	-0.34	**-0.71**	**-1.22**	
						1	(+)		285	0.72	0.83	0.95	145	0.72	0.83	0.96	145	0.72	0.83	0.94		0.00	-0.17	-0.05	0.00	0.17	-0.08	0.22	0.37	-0.35	**-0.50**	**-0.60**	**-1.19**	
						1	(-)	45~	183	0.76	0.88	1.00	84	0.76	0.88	0.99	99	0.76	0.88	1.01								-0.03	**-0.38**					
						1	(+)		149	0.76	0.88	1.01	66	0.75	0.88	0.99	83	0.76	0.88	1.02		0.00	0.11	0.06	-0.16	0.00	-0.04	-0.07	**-0.40**					
**Glu**	**mmol/L**	0.1	0.00	**0.62**	**0.59**	1	(-)	All	512	3.9	4.9	6.2	247	4.0	4.9	6.3	268	3.9	4.8	6.1	**Glu**							0.17	0.34					Partition by age, LAVE-
						1	(+)		395	3.9	4.8	6.1	195	4.1	4.9	6.4	224	3.9	4.8	6.1		0.00	-0.17	0.00	0.18	-0.17	0.00	0.36	**0.53**					
						1	(-)	~45	345	3.9	4.7	5.8	173	3.9	4.8	5.8	172	3.8	4.6	5.7								0.21	0.21	**-1.02**	**-3.75**	**-0.89**	**-1.78**	
						1	(+)		287	3.9	4.7	5.7	146	3.9	4.7	5.8	151	3.8	4.6	5.8		0.00	0.00	0.00	0.22	0.00	-0.20	0.22	0.00	**-1.02**	**-4.94**	**-0.89**	**-1.43**	
						1	(-)	45~	172	4.4	5.2	7.3	77	4.5	5.3	8.0	97	4.3	5.2	6.7								0.27	**1.76**					
						1	(+)		144	4.4	5.2	7.1	62	4.5	5.2	8.7	83	4.3	5.2	6.6		0.00	0.00	0.00	0.29	**-0.65**	0.17	0.29	**3.05**					
**TC**	**mmol/L**	0.1	0.00	0.37	**0.41**	1	(-)	All	532	3.1	4.6	6.7	260	3.1	4.7	6.9	273	3.1	4.6	6.6	**TC**							0.00	0.33					Partition by age for female. LAVE -
						1	(+)		396	3.2	4.5	6.6	198	3.2	4.7	6.7	219	3.3	4.6	6.6		-0.12	-0.11	-0.24	0.12	0.22	0.00	-0.12	0.12					
						1	(-)	~45	350	3.1	4.5	6.4	176	3.1	4.5	6.6	174	3.1	4.4	6.2								0.00	**0.48**	0.00	**-0.83**	-0.11	**-1.01**	
						1	(+)		285	3.1	4.4	6.1	145	3.2	4.5	6.3	145	3.1	4.3	6.1		0.00	-0.13	0.00	**0.39**	**0.38**	0.13	0.13	0.26	-0.34	**-1.01**	**-0.48**	**-1.07**	
						1	(-)	45~	183	3.2	5.0	7.2	84	3.1	5.1	7.4	99	3.2	4.9	7.1								-0.10	0.29					
						1	(+)		149	3.5	4.9	6.9	66	3.5	5.0	7.2	83	3.5	4.8	7.0		-0.35	**-0.42**	-0.34	0.35	0.21	0.11	0.00	0.23					
**TG**	**mmol/L**	0.01	0.23	**0.55**	**0.59**	1	(-)	All	528	0.49	1.03	3.49	258	0.50	1.17	4.14	272	0.47	0.91	2.85	**TG**							0.04	**1.69**					Partition by age, LAVE+
						1	(+)		403	0.49	0.96	2.95	204	0.52	1.19	3.51	225	0.47	0.89	2.70		0.00	-0.03	0.00	**0.86**	**0.83**	0.26	0.08	**1.29**					
						1	(-)	~45	346	0.49	0.90	2.99	175	0.51	1.07	3.29	173	0.46	0.78	2.29								0.08	**1.57**	-0.05	**-2.53**	-0.15	**-1.94**	
						1	(+)		289	0.47	0.86	2.60	149	0.50	1.02	2.88	146	0.45	0.75	2.03		0.04	0.02	0.02	**0.72**	**0.68**	**0.65**	0.09	**1.56**	-0.04	**-1.30**	-0.18	**-2.13**	
						1	(-)	45~	181	0.55	1.32	4.26	83	0.56	1.49	5.64	99	0.55	1.18	3.47								0.01	**2.29**					
						1	(+)		154	0.55	1.25	3.70	71	0.53	1.41	3.87	84	0.55	1.17	3.24		0.00	0.04	0.00	**0.70**	**2.08**	0.34	-0.02	**0.78**					
**HDL-C**	**mmol/L**	0.1	0.31	0.00	0.00	1	(-)	All	530	0.7	1.1	1.7	258	0.7	1.1	1.7	271	0.8	1.2	1.8	**HDL-C**							**-0.39**	**-0.39**					No partition by age or sex.

									RIs by parametric method													LAVE effect on LL			LAVE effect on UL			btw-sex diff		btw-age diff		btw-age diff		Decision
				M	F				M + F				M				F					M+F	M	F	M+F	M	F			M		F		
Item	Units	RU	SDR sex	SDR age		LAVE criteria	LAVE	Age	n	LL	Me	UL	n	LL	Me	UL	n	LL	Me	UL	Item	BR_{LL}			BR_{UL}			BR_{LL}	BR_{UL}	BR_{LL}	BR_{UL}	BR_{LL}	BR_{UL}	
HDL-C	mmol/L	0.1	0.31	0.00	0.00	1	(+)	All	395	0.8	1.2	1.8	198	0.7	1.1	1.7	218	0.8	1.2	1.8	HDL-C	**-0.39**	0.00	0.00	**-0.39**	0.00	0.00	**-0.39**	**-0.39**					LAVE- with its effect of ≤2RU
HDLrat		0.1	0.34	0.38	**0.45**	1	(-)	All	530	2.5	4.0	7.0	259	**2.6**	4.3	**7.6**	271	2.5	3.8	6.1	HDLrat							0.09	**1.31**					Partition by age (female). LAVE-
						1	(+)		396	2.6	3.9	6.3	198	2.8	4.3	7.2	219	2.5	3.8	5.8		-0.11	-0.18	0.00	**0.74**	0.36	0.36	0.32	**1.48**					
						1	(-)	~45	348	2.5	3.9	6.6	175	2.7	4.2	7.2	174	**2.4**	3.6	**5.9**								0.29	**1.24**	0.16	**-0.47**	-0.33	**-0.87**	
						1	(+)		285	2.6	3.8	6.1	144	2.8	4.1	6.7	145	2.4	3.6	5.7		-0.11	-0.10	0.00	**0.56**	**0.50**	0.24	**0.45**	**1.12**	0.27	**-0.53**	-0.36	**-0.83**	
						1	(-)	45~	182	2.7	4.3	7.5	83	2.5	4.7	7.8	98	**2.7**	4.0	**6.7**								-0.16	**0.90**					
						1	(+)		149	2.7	4.2	7.4	66	2.5	4.6	7.3	82	2.7	4.0	6.4		0.00	0.00	0.00	0.08	**0.41**	0.32	-0.17	**0.75**					
nonHDL	mmol/L	0.1	0.00	0.32	0.35	1	(-)	All	532	**2.0**	3.4	**5.6**	260	2.1	3.5	5.9	273	2.0	3.3	5.3	nonHDL							0.11	**0.65**					No partition by sex or age. LAVE-
						1	(+)		409	2.0	3.4	5.3	203	2.1	3.5	5.6	223	2.0	3.3	5.2		0.00	0.00	0.00	0.36	0.34	0.12	0.12	**0.48**					
LDL-C	mmol/L	0.1	0.00	0.35	**0.43**	1	(-)	All	533	1.5	2.8	4.7	260	**1.6**	2.9	**4.8**	273	1.6	2.8	4.6	LDL-C							0.00	0.25					Partition by age for female. LAVE-.
						1	(+)		396	1.7	2.8	4.5	198	1.7	2.9	4.8	219	1.6	2.8	4.5		-0.28	-0.13	0.00	0.28	0.00	0.14	0.14	**0.42**					
						1	(-)	~45	350	1.5	2.7	4.5	176	1.6	2.8	4.7	174	**1.5**	2.6	**4.2**								0.13	**0.65**	-0.12	-0.37	-0.26	**-0.91**	
						1	(+)		285	1.6	2.7	4.3	145	1.7	2.8	4.6	145	1.5	2.6	4.1		-0.15	-0.14	0.00	0.29	0.14	0.15	0.29	**0.73**	-0.25	**-0.76**	**-0.54**	**-1.08**	
						1	(-)	45~	183	1.7	3.1	5.0	84	1.7	3.2	5.0	99	**1.7**	3.0	**4.9**								0.00	0.12					
						1	(+)		149	1.9	3.1	5.0	66	1.9	3.2	5.2	83	1.9	3.0	4.9		-0.25	-0.24	-0.26	0.00	-0.24	0.00	0.00	**0.38**					
Lip	U/L	1	0.06	0.16	0.14	1	(-)	All	527	**9**	27	**68**	257	8	29	75	272	10	25	63	Lip							-0.13	**0.80**					No partition by sex or age. LAVE-
						1	(+)		392	9	26	64	194	8	29	71	218	9	24	60		0.00	0.00	0.08	0.29	0.25	0.23	-0.07	**0.78**					
ALT	IU/L	1	**0.59**	0.20	**0.51**	1	(-)	All	528	8	18	55	259	10	22	64	269	7	15	35	ALT							0.25	**2.42**					Partition by sex, LAVE+. No partiition by age
						1	(+)		412	8	16	39	202	**10**	22	**55**	229	**7**	14	**30**		0.00	0.00	0.00	**2.02**	**0.78**	**0.85**	**0.38**	**3.16**					
						1	(-)	~45	347	7	16	51	176	9	21	61	173	7	13	33								0.18	**2.49**	-0.22	**-0.65**	0.00	**-0.42**	
						1	(+)		283	8	15	38	144	9	20	47	144	7	13	29		-0.13	0.00	0.00	**1.70**	**1.44**	**0.71**	0.26	**2.35**	-0.35	**-1.31**	-0.17	0.00	
						1	(-)	45~	181	10	20	60	84	12	25	70	96	7	18	36								**0.39**	**2.67**					
						1	(+)		148	10	19	48	66	13	25	62	81	8	17	29		0.00	-0.08	-0.19	**1.24**	**0.64**	**1.31**	**0.52**	**3.40**					
AST	IU/L	1	**0.49**	0.00	0.35	1	(-)	All	525	16	23	39	258	17	25	45	266	15	21	32	AST							0.34	**2.22**					Partition by sex. LAVE+
						1	(+)		409	16	22	33	206	**17**	25	**40**	227	**15**	21	**29**		0.00	0.00	0.00	**1.38**	**0.85**	**0.84**	**0.46**	**2.54**					
ALP	IU/L	1	0.00	0.17	**0.69**	1	(-)	All	531	50	85	153	260	**53**	88	**153**	270	49	83	156	ALP							0.15	-0.11					Partition by age for females. LAVE-.
						1	(+)		394	50	84	151	198	54	87	153	218	48	81	148		0.00	-0.04	0.04	0.08	0.00	0.31	0.23	0.19					
						1	(-)	~45	347	50	81	142	176	53	86	153	172	**47**	77	**130**								0.26	**0.98**	0.04	0.08	-0.29	**-1.61**	
						1	(+)		282	50	80	142	145	54	87	156	144	47	75	129		0.00	-0.04	0.00	0.00	-0.12	0.05	0.30	**1.15**	0.16	0.00	-0.24	**-1.41**	
						1	(-)	45~	183	54	95	163	84	52	92	151	99	**55**	100	**174**								-0.11	**-0.83**					
						1	(+)		149	53	94	160	66	50	90	156	83	53	96	165		0.04	0.07	0.07	0.11	-0.18	0.32	-0.11	-0.33					
AMY	IU/L	1	0.02	0.00	0.00	1	(-)	All	530	**47**	87	**164**	258	48	87	163	272	45	85	160	AMY							0.10	0.10					No partiion by sex or age. LAVE-
						1	(+)		394	47	87	154	197	49	90	161	218	44	85	154		0.00	-0.04	0.04	0.37	0.07	0.21	0.18	0.26					
LDH	IU/L	1	0.00	0.00	0.28	1	(-)	All	530	**138**	189	**257**	259	136	187	255	271	139	190	259	LDH							-0.10	-0.13					No partiion by sex or age. LAVE-
						1	(+)		409	136	187	249	207	139	186	250	225	138	189	250		0.07	-0.11	0.04	0.28	0.18	0.32	0.03	0.00					
CK	IU/L	1	**0.52**	0.00	0.08	1	(-)	All	529	61	139	380	259	**72**	167	**460**	268	**53**	119	**260**	CK							0.23	**2.46**					Partition by sex. LAVE-
						1	(+)		406	58	135	332	203	76	172	440	219	54	119	277		0.04	-0.04	-0.02	**0.69**	0.22	-0.30	0.31	**2.33**					
GGT	IU/L	1	**0.54**	0.29	0.13	1	(-)	All	524	12	25	88	257	12	31	105	269	**10**	21	**50**	GGT							0.10	**2.84**					Partition by sex. LAVE+ for males. LAVE- females
						1	(+)		405	11	24	65	199	**13**	31	**90**	224	10	21	47		0.07	-0.05	0.00	**1.67**	**0.76**	0.32	0.22	**3.12**					
CRP	mg/L	0.01	0.11	0.35	0.17	1	(-)	All	528	0.22	1.22	18.86	255	0.20	1.01	14.35	273	0.23	1.54	20.2	CRP							-0.01	**-1.23**					No partiion by sex or age. LAVE+
						1	(+)		425	**0.21**	1.10	**14.67**	200	0.20	0.97	12.97	216	0.23	1.41	13.9		0.00	0.00	0.00	**1.14**	**0.42**	**1.82**	-0.01	-0.24					
Fe	µmol/L	0.1	0.39	0.10	0.04	3	(-)	All	526	4.3	15.8	29.0	253	7.7	17.5	29.9	273	3.9	13.7	29.3	Iron							**0.60**	0.10					No partiion by sex or age. LAVE+
						3	(+)		412	**8.8**	16.1	**28.9**	207	9.8	17.4	28.7	207	7.5	14.6	29.3		**-0.88**	**-0.44**	**-0.65**	0.02	0.25	0.00	**0.45**	-0.12					
Ferr	µg/L	**1**	**0.81**	0.20	**0.44**	3	(-)	All	526	1	63	404	253	**10**	120	**475**	272	2	31	184	Ferr							0.08	**2.83**					Partition by sex and age for female. LAVE-
						3	(+)		404	9	74	413	202	20	124	457	206	7	38	193		-0.08	-0.09	-0.11	-0.09	0.16	-0.19							
						3	(-)	~45	348	1	58	358	174	9	121	366	173	**2**	24	**150**								0.08	**2.37**	0.03	**-2.54**	0.04	**-1.55**	
						3	(+)		281	6	71	366	147	18	123	371	141	5	28	150		-0.05	-0.10	-0.08	-0.09	-0.06	0.00			0.02	**-2.50**	-0.15	**-1.73**	
						3	(-)	45~	177	1	71	450	78	6	122	667	99	**0**	48	**222**								0.05	**3.89**					
						3	(+)		134	13	81	452	65	16	131	650	72	12	62	232		-0.11	-0.06	-0.21	-0.02	0.11	-0.18							
Tf	g/L	0.1	**0.44**	0.15	0.18	3	(-)	All	525	1.9	2.5	3.7	254	**1.9**	2.4	**3.2**	273	2.0	2.7	3.9	TF							-0.22	**-1.52**					Partition by sex. LAVE+ for female
						3	(+)		408	1.9	2.5	3.3	203	1.8	2.4	3.1	206	**2.0**	2.6	**3.5**		0.00	0.30	0.00	**1.12**	0.30	**1.05**							
TfSat	%	1	**0.50**	0.00	0.00	3	(-)	All	524	5	25	46	252	11	29	48	273	4	20	44	TFSat							**0.67**	**0.38**					Partition by sex. LAVE+
						3	(+)		416	13	26	47	208	**15**	28	**49**	211	**10**	22	**44**		**-0.92**	**-0.46**	**-0.69**	-0.12	-0.12	0.00							
IgG	g/L	0.1	0.11	0.18	0.00	2	(-)	All	525	**11.1**	14.7	**20.0**	254	11.1	14.6	19.4	270	10.9	15.0	20.5	IgG							0.07	**-0.52**					No partiion by sex or age. LAVE-
						2	(+)		428	11.1	14.7	19.4	201	11.5	14.7	19.3	218	11.2	14.8	19.5		-0.04	-0.21	-0.10	0.29	0.05	**0.51**							
IgA	g/L	0.01	0.00	0.15	0.30	2	(-)	All	526	**1.05**	2.32	**4.63**	254	1.02	2.37	4.72	273	1.03	2.28	4.54	IgA							-0.01	0.20					No partiion by sex or age. LAVE-
						2	(+)		423	1.02	2.35	4.42	198	0.99	2.40	4.44	218	1.02	2.23	4.35		0.03	0.03	0.01	0.24	0.32	0.22							
IgM	g/L	0.01	**0.50**	0.16	0.07	2	(-)	All	526	0.41	1.02	2.58	254	**0.38**	0.85	**2.29**	272	**0.51**	1.21	**2.77**	IgM							-0.23	**-0.87**					Partition by sex. LAVE-
						2	(+)		417	0.41	1.02	2.60	197	0.40	0.85	2.37	217	0.51	1.20	2.68		0.00	-0.04	0.00	-0.04	-0.16	0.16							
FT3	pmol/L		0.12	0.00	0.09	4	(-)	All	467	**3.9**	4.8	**6.3**	235	4.0	4.9	6.2	233	3.8	4.8	6.4	FT3							0.33	-0.33					No partiion by sex or age
FT4	pmol/L		0.00	0.34	0.38	4	(-)	All	468	**7.8**	10.6	**14.1**	235	7.7	10.7	14.3	234	7.8	10.5	13.8	FT4							-0.06	0.31					No partiion by sex or age
TSH	mIU/L		0.00	0.00	0.00	4	(-)	All	461	**0.61**	1.60	**4.86**	231	0.63	1.63	5.10	232	0.51	1.59	4.53	TSH							0.11	**0.53**					No partiion by sex or age
PSA	µg/L			0.39			(-)	All					243	**0.29**	0.77	**2.92**					PSA													No partiion by age

									RIs by parametric method													LAVE effect on LL			LAVE effect on UL			btw-sex diff		btw-age diff		btw-age diff		Decision
				M	F				M + F				M				F					M+F	M	F	M+F	M	F			M		F		
Item	Units	RU	SDR sex	SDR age		LAVE criteria	LAVE	Age	n	LL	Me	UL	n	LL	Me	UL	n	LL	Me	UL	Item	BR_{LL}			BR_{UL}			BR_{LL}	BR_{UL}	BR_{LL}	BR_{UL}	BR_{LL}	BR_{UL}	

***Reference tests used for LAVE**

1. Alb, Glb, UA, Glu, TG, nonHDL, AST, ALT, LDH, GGT, CK, CRP
2. TP, Alb, Glb, IgG, IgA, IgM, CRP, WBC, PLT
3. Ferr, Fe, Tf, TfSat, Hb, Hct, MCV
4. Individuals positive with anti-thyroid antibodies were excluded.

Note:

1. SDRsex and SDRage: Bold interface represents value >0.4
2. Reporting unit (RU) represents a unit of value rounded up for reporting test results. If number of digit below decimal point is 2, 1, or 0, RU is 0.01, 0.1, or 1, respectively.
   If the width of RI (UL-LL) is comprised of more than 50 RU, we compute (UL-LL)×0.02 and round it up for use as RU.
   For example, if RI = 20~180, we first compute (180-20) x 0.02 = 3.3 and round it up to 5 for use as RU. Similarly, if RU=0.1 and RI=1.8~13.4, (13.4-1.8) x 0.02 =0.232 was rounded up as 0.2 for RU.
   RU was referred to when between subgroup difference at LL or UL expressed as BiasLL or BiasUL do not match with actual differences, which should be ≥3RU for partitioning.
3. Red bold font represents proposed RIs
4. BRLL and BRUL : Bold interface represents values < -0.375 or > 0.375
