# Supplementary material for: Determination of reference intervals for common chemistry and immunoassay tests for Kenyan adults based on an internationally harmonized protocol and up-to-date statistical methods
Source: PLoS One. 2020 Jul 9;15(7):e0235234. doi: 10.1371/journal.pone.0235234 (PMC7347104; doi:10.1371/journal.pone.0235234)
Supplement: S2 Table — (PDF) [file pone.0235234.s006.pdf]

S2 Table. Comparison of reference intervals

		PRESENT STUDY KENYA							BECKMAN AU (20)							SAUDI ARABIA (10)						
			M+F		M		F			M+F		M		F			M+F		M		F	
Test	Units	Age	LL	UL	LL	UL	LL	UL	Age	LL	UL	LL	UL	LL	UL	Age	LL	UL	LL	UL	LL	UL
TP	g/L	18-65	67	83					NA	66	83					18-65	66	83				
Alb	g/L	18-65							NA	35	52					18-65	39	50				
		18-44			40	51	38	49														
		45-65			40	47	38	46														
Urea	mmol/L	18-65			2.4	6.6			NA	2.8	7.2					18-65			2.8	7.3	2.1	6.4
		18-44					1.8	5.3														
		45-65					1.9	6.2														
UA	µmol/L	18-65			243	507	178	417	NA			208.3	428.4	154.7	357	18-65			223	444	148	321
Cre	µmol/L	18-65			58	109	45	86	NA			59	104	45	84	18-65			66	111	50	74
TBil	µmol/L	18-65			6	43	5	27	NA	5	21					18-65			3.6	22.4	2.2	15.5
Na	mmol/L	18-65	134	142					NA	136	146					18-65	135	144				
K	mmol/L	18-65	3.4	4.8					NA	3.5	5.1					18-65	3.7	4.9				
Cl	mmol/L	18-65	100	110					NA	101	109					18-65	101	111				
Ca	mmol/L	18-65	2.19	2.57					NA	2.2	2.65					18-65	2.11	2.56				
IP	mmol/L	18-65	0.78	1.42					NA	0.81	1.45					18-65	0.81	1.44				
Mg	mmol/L	18-65			0.73	0.98			NA			0.73	1.06	0.77	1.03	18-65	0.71	0.96				
		18-44					0.71	0.93														
		45-65					0.76	1.01														
Glu	mmol/L	18-65							NA	4.1	5.9					18-65	4	5.9				
		18-44	3.9	5.8																		
		45-65	4.4	7.3																		
TC	mmol/L	18-65							NA		5.2					18-65	3.5	6.36				
		18-45	3.10	6.10																		
		46-65	3.20	7.20																		
TG	mmol/L	18-65							NA		1.7					18-65			0.5	3.58	0.39	1.6
		18-45			0.50	2.88	0.45	2.03														
		46-65			0.53	3.87	0.55	3.24														
HDL-C	mmol/L	18-65	0.70	1.70					NA	1.03	1.55					18-65			0.74	1.76	0.98	2.19
LDL-C	mmol/L	18-65			1.60	4.80			NA		2.60					18-65	1.8	4.34				
		18-44					1.50	4.20														
		45-65					1.70	4.90														
Lip	U/L	18-65			8	75	10	63	NA		67											
ALT	IU/L	18-65			10	55	7	30	NA				50		35	18-65			7	39	5	18
AST	IU/L	18-65			17	40	15	29	NA				50		35	18-65			11	28	10	24
ALP	IU/L	18-65			53	153			NA	30	120						39	114				
		18-44					47	130														
		45-65					55	174														
AMY	IU/L	18-65	47	164					NA	22	80					18-65	31	117				
LDH	IU/L	18-65	138	257					NA				248		247	18-65	10	238				
CK	IU/L	18-65			72	460	53	260	NA				171		145	18-65			54	266	27	138
GGT	IU/L	18-65			13	90	10	47	NA				55		38	18-65			11	65	7	21
Fe	µmol/L	18-65	8.8	28.9					NA			12.5	32.2	10.7	32.2	18-65			7.9	29.6	3.7	26
Ferr	µg/L	18-65			20	457			NA			20	250	10	120							
		18-44					5	150														
		45-65					12	232														
Tf	g/L	18-65			1.9	3.2	2.0	3.5	NA	2	3.6					18-65			2	3.2	2.1	3.9
TfSat	%	18-65	13	47																		
CRP	mg/L	18-65	0.21	14.7					NA		1					18-65	0.2	11.8				
IgA	g/L	18-65	1.05	4.63					NA	0.7	4											
IgG	g/L	18-65	11.1	20.0					NA	7	16											
IgM	g/L	18-65			0.38	2.29	0.51	2.77	NA	0.4	2.3											
FT3	pmol/L	18-65	3.9	6.3					NA	3.8	6											
FT4	pmol/L	18-65	7.8	14.1					NA	7.86	14.41											
TSH	mIU/L	18-65	0.61	4.86					NA	0.38	5.33											
PSA	µg/L	18-65			0.25	2.95																

S2 Table. Comparison of reference intervals

		PRESENT STUDY KENYA							TURKEY (11)							INDIA (13)						
			M+F		M		F			M+F		M		F			M+F		M		F	
Test	Units	Age	LL	UL	LL	UL	LL	UL	Age	LL	UL	LL	UL	LL	UL	Age	LL	UL	LL	UL	LL	UL
TP	g/L	18-65	67	83					20-79	66	82					18-65	68	86				
Alb	g/L	18-65							20-79	41	49					18-65					36	47
		18-44			40	51	38	49														
		45-65			40	47	38	46														
Urea	mmol/L	18-65			2.4	6.6										18-65			2.2	6.0		
		18-44					1.8	5.3	20-49			2.95	7.20	2.21	6.12	18-45					1.9	5.1
		45-65					1.9	6.2	50-79					2.85	7.96	46-65					2.4	6.7
UA	µmol/L	18-65			243	507	178	417	20-79			226	458	166	345	18-65			248	509	159	404
Cre	µmol/L	18-65			58	109	45	86	20-79			59	92	50	71	18-65			58	95	35	74
TBil	µmol/L	18-65			6	43	5	27	20-79			3.8	24.1	2.7	15.9	18-65			6.2	23.7	4	17.3
Na	mmol/L	18-65	134	142					20-79	137	144					18-65	135	146				
K	mmol/L	18-65	3.4	4.8					20-79	3.7	4.9					18-65	3.8	5				
Cl	mmol/L	18-65	100	110					20-79	99	107					18-65	102	113				
Ca	mmol/L	18-65	2.19	2.57					20-79	2.15	2.47					18-65	2.1	2.44				
IP	mmol/L	18-65	0.78	1.42					20-79	0.8	1.4					18-65	0.8	1.43				
Mg	mmol/L	18-65			0.73	0.98			20-79	0.77	1.06					18-65	0.77	1.07				
		18-44					0.71	0.93														
		45-65					0.76	1.01														
Glu	mmol/L	18-65							20-79	3.96	5.88					18-65						
		18-44	3.9	5.8												18-45	4.1	5.5				
		45-65	4.4	7.3												46-65	4.3	6.0				
TC	mmol/L	18-65														18-65					2.9	6.6
		18-45	3.10	6.10					20-49	3.22	6.45	3.20	6.42	3.20	6.38	18-45			3.1	6.2		
		46-65	3.20	7.20					50-79					3.93	7.92	46-65			2.5	6.7		
TG	mmol/L	18-65														18-65			0.6	2.7	0.5	2.1
		18-45			0.50	2.88	0.45	2.03	20-49			0.53	3.39	0.46	2.52	18-45						
		46-65			0.53	3.87	0.55	3.24	50-79					0.64	3.55	46-65						
HDL-C	mmol/L	18-65	0.70	1.70								0.85	1.52	0.95	1.56	18-65			0.7	1.5	0.8	1.8
LDL-C	mmol/L	18-65			1.60	4.80										18-65	1.7	4.4				
		18-44					1.50	4.20	20-49	1.47	3.92	1.60	4.01	1.32	3.92	18-45						
		45-65					1.70	4.90	50-79					1.78	4.91	46-65						
Lip	U/L	18-65			8	75	10	63								18-65						
ALT	IU/L	18-65			10	55	7	30				9	57	7	28	18-65			15	74	10	37
AST	IU/L	18-65			17	40	15	29				13	30	11	25	18-65			20	53	17	39
ALP	IU/L	18-65			53	153										18-65			41	111		
		18-44					47	130	20-49	38	112	43	116	34	97	18-45					35	100
		45-65					55	174	50-79					47	133	46-65					43	117
AMY	IU/L	18-65	47	164						34	119					18-65	36	135				
LDH	IU/L	18-65	138	257						126	220					18-65	104	206				
CK	IU/L	18-65			72	460	53	260				48	227	34	131	18-65			48	304	36	184
GGT	IU/L	18-65			13	90	10	47				11	69	7	33	18-65			14	62	11	40
Fe	µmol/L	18-65	8.8	28.9								5.9	31.6	3.5	27.8	18-65			7	33	4	26
Ferr	µg/L	18-65			20	457			18-79					4.7	136	18-65						
		18-44					5	150	18-44			13	276	4.3	91							
		45-65					12	232	45-79					5.9	175							
Tf	g/L	18-65			1.9	3.2	2.0	3.5	18-79	1.8	3.3	1.8	3.3	1.9	3.5	18-65	2.2	4				
TfSat	%	18-65	13	47												18-65						
CRP	mg/L	18-65	0.21	14.7												18-65			0.33	7.34	0.35	11.9
IgA	g/L	18-65	1.05	4.63												18-65	0.94	4.35				
IgG	g/L	18-65	11.1	20.0												18-65	9.1	20.4				
IgM	g/L	18-65			0.38	2.29	0.51	2.77								18-65			0.4	2.54	0.51	3.1
FT3	pmol/L	18-65	3.9	6.3												18-65						
FT4	pmol/L	18-65	7.8	14.1												18-65						
TSH	mIU/L	18-65	0.61	4.86												18-65						
PSA	µg/L	18-65			0.25	2.95										18-65						

S2 Table. Comparison of reference intervals

		PRESENT STUDY KENYA							US-MGH (2)							KENYA-KERICHO (16)						
			M+F		M		F			M+F		M		F			M+F		M		F	
Test	Units	Age	LL	UL	LL	UL	LL	UL	Age	LL	UL	LL	UL	LL	UL	Age	LL	UL	LL	UL	LL	UL
TP	g/L	18-65	67	83					NA	55	80											
Alb	g/L	18-65							NA	35	55					18-55	35.8	48.1	36.9	48.5	34.4	47.5
		18-44			40	51	38	49														
		45-65			40	47	38	46														
Urea	mmol/L	18-65			2.4	6.6			NA	3.6	7.1					18-55	1.4	4.6	1.5	4.6	1.4	4.6
		18-44					1.8	5.3	NA													
		45-65					1.9	6.2	NA													
UA	µmol/L	18-65			243	507	178	417	NA			150	480	90	360							
Cre	µmol/L	18-65			58	109	45	86	NA		133					18-55	55	102	62	106	51	91
TBil	µmol/L	18-65			6	43	5	27	NA	5.1	17					18-55	4.9	39.9	5.6	41.9	4.4	26.8
Na	mmol/L	18-65	134	142					NA	136	145					18-55	141	153	142	152	140	155
K	mmol/L	18-65	3.4	4.8					NA	3.5	5.0					18-55	3.9	5.8	3.9	5.8	3.8	5.8
Cl	mmol/L	18-65	100	110					NA	98	106					18-55	100.5	112	100	111	101	113
Ca	mmol/L	18-65	2.19	2.57					NA	2.2	2.6											
IP	mmol/L	18-65	0.78	1.42					NA	1.0	1.4											
Mg	mmol/L	18-65			0.73	0.98			NA	0.8	1.2											
		18-44					0.71	0.93														
		45-65					0.76	1.01														
Glu	mmol/L	18-65							NA	4.2	6.4											
		18-44	3.9	5.8					NA													
		45-65	4.4	7.3					NA													
TC	mmol/L	18-65							NA							18-55	2.6	5.7	2.5	5.5	2.6	5.9
		18-45	3.10	6.10					NA													
		46-65	3.20	7.20					NA													
TG	mmol/L	18-65							NA		1.8					18-55	0.4	2.6	0.4	2.7	0.4	2.5
		18-45			0.50	2.88	0.45	2.03	NA													
		46-65			0.53	3.87	0.55	3.24	NA													
HDL-C	mmol/L	18-65	0.70	1.70					NA	1.03												
LDL-C	mmol/L	18-65			1.60	4.80			NA													
		18-44					1.50	4.20	NA													
		45-65					1.70	4.90	NA													
Lip	U/L	18-65			8	75	10	63	NA	0	160											
ALT	IU/L	18-65			10	55	7	30	NA	0	35					18-55	9.6	52	10.8	53.9	8.6	47
AST	IU/L	18-65			17	40	15	29	NA	0	35					18-55	13.8	42.3	14.9	45.3	13.1	38.1
ALP	IU/L	18-65			53	153			NA	30	120											
		18-44					47	130	NA													
		45-65					55	174	NA													
AMY	IU/L	18-65	47	164					NA	60	180					18-55	38.3	163.0	40.0	171.3	36.0	147.8
LDH	IU/L	18-65	138	257					NA	100	190					18-55	126.0	263.9	124.0	259.0	131.5	295.4
CK	IU/L	18-65			72	460	53	260	NA			60	400	40	150							
GGT	IU/L	18-65			13	90	10	47	NA	1	94											
Fe	µmol/L	18-65	8.8	28.9					NA	9	27											
Ferr	µg/L	18-65			20	457			NA			30	300	10	200							
		18-44					5	150														
		45-65					12	232														
Tf	g/L	18-65			1.9	3.2	2.0	3.5	NA	2.3	3.9											
TfSat	%	18-65	13	47					NA	20	45											
CRP	mg/L	18-65	0.21	14.7					NA	0.02	8											
IgA	g/L	18-65	1.05	4.63					NA	0.6	3.09											
IgG	g/L	18-65	11.1	20.0					NA	6.14	12.95											
IgM	g/L	18-65			0.38	2.29	0.51	2.77	NA	0.53	3.34											
FT3	pmol/L	18-65	3.9	6.3					NA	0.22	6.78											
FT4	pmol/L	18-65	7.8	14.1					NA	10.3	35											
TSH	mIU/L	18-65	0.61	4.86					NA	0.5	4.7											
PSA	µg/L	18-65			0.25	2.95			NA	0	4											

S2 Table. Comparison of reference intervals

		PRESENT STUDY KENYA							KENYA-NAIROBI, KILIFI (19)						
			M+F		M		F			M+F		M		F	
Test	Units	Age	LL	UL	LL	UL	LL	UL	Age	LL	UL	LL	UL	LL	UL
TP	g/L	18-65	67	83					18-60	58	88	59	88	58	87
Alb	g/L	18-65							18-60	35	52	36	52	35	51
		18-44			40	51	38	49							
		45-65			40	47	38	46							
Urea	mmol/L	18-65			2.4	6.6									
		18-44					1.8	5.3							
		45-65					1.9	6.2							
UA	µmol/L	18-65			243	507	178	417							
Cre	µmol/L	18-65			58	109	45	86	18-60	47	109	52	114	43	99
TBil	µmol/L	18-65			6	43	5	27	18-60	2.9	37	3.6	41.9	2.7	31.5
Na	mmol/L	18-65	134	142											
K	mmol/L	18-65	3.4	4.8											
Cl	mmol/L	18-65	100	110											
Ca	mmol/L	18-65	2.19	2.57											
IP	mmol/L	18-65	0.78	1.42											
Mg	mmol/L	18-65			0.73	0.98									
		18-44					0.71	0.93							
		45-65					0.76	1.01							
Glu	mmol/L	18-65													
		18-44	3.9	5.8											
		45-65	4.4	7.3											
TC	mmol/L	18-65													
		18-45	3.10	6.10											
		46-65	3.20	7.20											
TG	mmol/L	18-65													
		18-45			0.50	2.88	0.45	2.03							
		46-65			0.53	3.87	0.55	3.24							
HDL-C	mmol/L	18-65	0.70	1.70											
LDL-C	mmol/L	18-65			1.60	4.80									
		18-44					1.50	4.20							
		45-65					1.70	4.90							
Lip	U/L	18-65			8	75	10	63							
ALT	IU/L	18-65			10	55	7	30	18-60	8	61	9	67	8	48
AST	IU/L	18-65			17	40	15	29	18-60	14	60	15	71	13	43
ALP	IU/L	18-65			53	153			18-60	48	164	48	138	46	179
		18-44					47	130							
		45-65					55	174							
AMY	IU/L	18-65	47	164					18-60	35	159	37	167	33	140
LDH	IU/L	18-65	138	257					18-60	213	678	209	691	217	652
CK	IU/L	18-65			72	460	53	260	18-60	53	552	60	709	49	354
GGT	IU/L	18-65			13	90	10	47							
Fe	µmol/L	18-65	8.8	28.9											
Ferr	µg/L	18-65			20	457									
		18-44					5	150							
		45-65					12	232							
Tf	g/L	18-65			1.9	3.2	2.0	3.5							
TfSat	%	18-65	13	47											
CRP	mg/L	18-65	0.21	14.7											
IgA	g/L	18-65	1.05	4.63											
IgG	g/L	18-65	11.1	20.0					18-60	7.74	28.33	7.56	28.04	7.92	28.95
IgM	g/L	18-65			0.38	2.29	0.51	2.77							
FT3	pmol/L	18-65	3.9	6.3											
FT4	pmol/L	18-65	7.8	14.1											
TSH	mIU/L	18-65	0.61	4.86											
PSA	µg/L	18-65			0.25	2.95									
